# Structural transformation of methasterone with *Cunninghamella blakesleeana* and *Macrophomina phaseolina*†

**DOI:** 10.1039/d2ra01396g

**Published:** 2022-03-25

**Authors:** Muhammad Aamer, Mahwish Siddiqui, Almas Jabeen, Rimsha Irshad, Farooq-Ahmad Khan, Atia-tul-Wahab, M. Iqbal Choudhary, Yan Wang

**Affiliations:** H. E. J. Research Institute of Chemistry, International Center for Chemical and Biological Sciences, University of Karachi Karachi-75270 Pakistan yan.wang@iccs.edu iqbal.choudhary@iccs.edu; Dr Panjwani Center for Molecular Medicine and Drug Research, International Center for Chemical and Biological Sciences, University of Karachi Karachi 75270 Pakistan; Third World Center (TWC) for Chemical Sciences, International Center for Chemical & Biological Sciences, University of Karachi 75270 Pakistan; Department of Biochemistry, Faculty of Science, King Abdulaziz University Jeddah-21589 Saudi Arabia

## Abstract

An anabolic-androgenic synthetic steroidal drug, methasterone (1) was transformed by two fungi, *Cunninghamella blakesleeana* and *Macrophimina phaseclina*. A total of six transformed products, 6β,7β,17β-trihydroxy-2α,17α-dimethyl-5α-androstane-3-one (2), 6β,7α,17β-trihydroxy-2α,17α-dimethyl-5α-androstane-3-one (3), 6α,17β-dihydroxy-2α,17α-dimethyl-5α-androstane-3,7-dione (4), 3β,6β,17β-trihydroxy-2α,17α-dimethyl-5α-androstane-7-one (5), 7α,17β-dihydroxy-2α,17α-dimethyl-5α-androstane-3-one (6), and 6β,9α,17β-trihydroxy-2α,17α-dimethyl-5α-androstane-3-one (7) were synthesized. Among those, compounds 2–5, and 7 were identified as new transformed products. MS, NMR, and other spectroscopic techniques were performed for the characterization of all compounds. Substrate 1 (IC_50_ = 23.9 ± 0.2 μg mL^−1^) showed a remarkable anti-inflammatory activity against nitric oxide (NO) production, in comparison to standard LNMMA (IC_50_ = 24.2 ± 0.8 μg mL^−1^). Whereas, its metabolites 2, and 7 showed moderate inhibition with IC_50_ values of 38.1 ± 0.5 μg mL^−1^, and 40.2 ± 3.3 μg mL^−1^, respectively. Moreover, substrate 1 was found to be cytotoxic for the human normal cell line (BJ) with an IC_50_ of 8.01 ± 0.52 μg mL^−1^, while metabolites 2–7 were identified as non-cytotoxic. Compounds 1–7 showed no cytotoxicity against MCF-7 (breast cancer), NCI-H460 (lung cancer), and HeLa (cervical cancer) cell lines.

## Abstract 2

Fungal transformation of methasterone resulted in six products (2–7). 2–5, and 7 were identified as new. Substrate 1 showed remarkable anti-inflammatory activity but was cytotoxic. Products 2 and 7 showed moderate activity but were non-cytotoxic.

## Graphical abstract

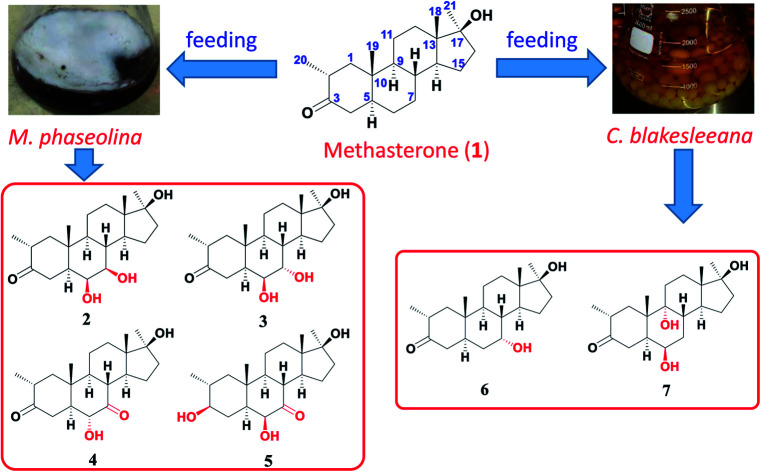

## Introduction

Biotransformation is a cost-effective and robust approach for the production of new and novel biologically active metabolites through living cell cultures of microorganisms, animals, and plants.^1,2^ These biological catalysts can trigger multiple reactions, such as hydroxylation, hydrolysis, reduction,^3^ aldol and reverse-aldol reactions,^1^ esterification,^4^ glycosylation,^5^ isomerization, epimerization, methylation,^6^ Michael addition, epoxidation, and various rearrangement reactions,^7^ thus resulting in highly stereo-, regio-, and chemo-selective products. To some extent, the use of toxic chemical catalysts has been reduced by biological catalysts, which are mostly eco-friendly and low-cost enzymes from various organisms.^8^ Therefore, biotransformation has become one of the most convincing techniques for the structural modifications of existing drugs and chemical intermediates.^9^

Methasterone (1) also known as methyldrostanolone due to its synthetic scheme,^10^ or trademarked names Methasteron™ and Superdrol™^11^ is an orally active anabolic-androgenic steroid (AAS), used to increase skeletal muscles growth. Consequently, athletes may favour methasterone (1) for its fat-burning ability and moderate anabolic properties.^12–14^ However, its toxicity has been observed in many cases.^11,15–17^ Thus methasterone (1) has been banned by World Anti-Doping Agency since 2006, and its use in- and out-of-competition is prohibited.^18^ In fact, the potential risk of AAS has become a great challenge due to their toxicity.^19,20^

There are several studies on metabolism of methasterone (1) has been reported.^10,18,21,22^ In our continuing studies of biotransformation on steroids,^23–31^ previously biocatalysis of methasterone (1) was performed by our research group, which resulted into several new anti-inflammatory compounds.^32^ Based on the previous results of compound 1, biotransformation of 1 was performed again by using *Macrophomina phaseolina* and *Cunninghamella blakesleeana*, which have been demonstrated to be effective cell cultures for bioconversion of steroids.^29,30,32,33^ Interestingly, more new structural analogues of 1 were synthesized. In addition, the anti-inflammatory activity and cytotoxicity of substrate 1 and all metabolites 2–7 were compared. It was found that biotransformation significantly decreased the cytotoxicity of derivatives of methasterone (1).

## Results and discussion

**Fig. 1:**
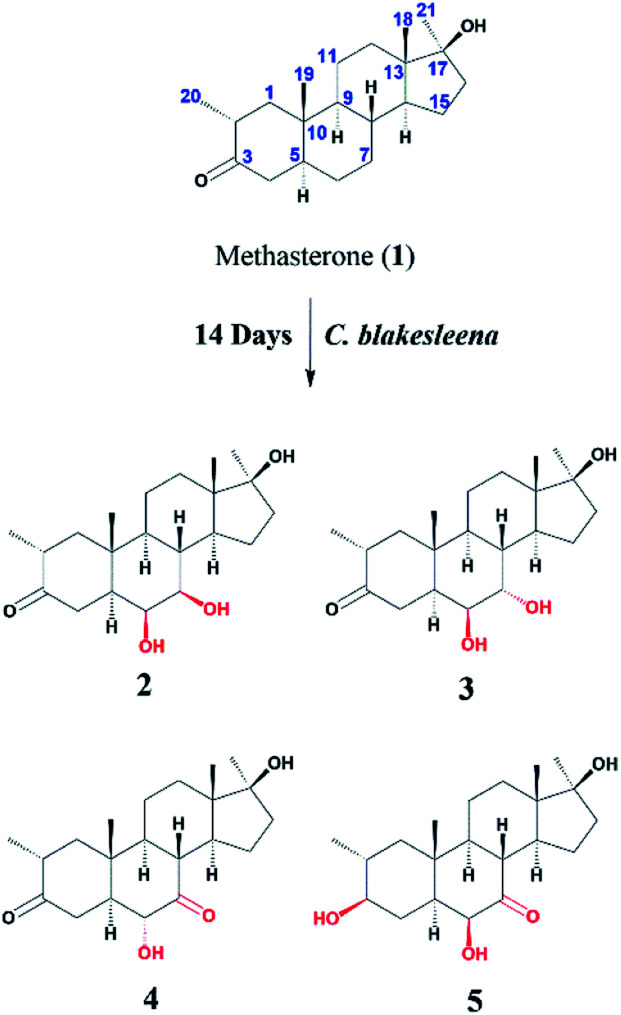
Biotransformation of methasterone (1) with *Macrophomina phaseolina*.

Compound 2 was isolated as a white crystalline solid (Fig. 1). The molecular formula of 2 showed the [M]^+^ at *m*/*z* 350.2458 (C_21_H_34_O_4,_ calcd 350.2457) through HREI-MS analysis. The absorbances in the IR spectrum at 3418 and 1703 cm^−1^ indicated the presence of hydroxyl and carbonyl groups, respectively. Compared with the NMR data of substrate 1,^32^ the ^1^H NMR spectrum (Table 1) of 2 showed the signals for two new downfield protons at *δ* 3.54 (dd, 3.5, 2.5 Hz) and 3.20 (dd 10.0, 3.5 Hz), which suggested that the di-hydroxylation of substrate 1. Meanwhile, in the ^13^C NMR spectrum (Table 2), two carbon signals appeared at *δ* 75.2 and 78.3, correlating with *δ* 3.54 and 3.20 in HSQC spectrum, respectively. The OH group at C-6 (*δ* 75.2) was placed on the basis of HMBC correlations of H-6 (*δ* 3.54) with C-5 (*δ* 50.2), C-7 (*δ* 78.3), and C-10 (*δ* 37.2). This was further supported by COSY cross-peaks of H-5 (*δ* 1.53) and H-7 (*δ* 3.20) with *δ* 3.54 (H-6) (Fig. 3). Similarly, the second OH group was placed at C-7 (*δ* 78.2) based on HMBC correlations of H-7 (*δ* 3.20) with C-5 (*δ* 50.2), C-6 (*δ* 75.2), and C-9 (*δ* 53.3), and the COSY correlations of H-7 (*δ* 3.20) with H-6 (*δ* 3.54) and H-8 (*δ* 1.81). The OH group at C-6 (*δ* 75.2) was deduced to be β-orientated due to the NOSEY correlations of H-6 (*δ* 3.54) with α-oriented H-5 (*δ* 1.53). Meanwhile, H-6 (*δ* 3.54) did not show any NOESY correlations with β-oriented H-8 (*δ* 1.81) and CH_3_-19 (*δ* 1.27), along with the coupling constant of H-6 at *δ* 3.54 (dd, 3.5, 2.5 Hz), further indicating the α-orientation of H-6. Likewise, the orientation of the OH group at C-7 (*δ* 78.2) was inferred through NOESY correlations of H-7 (*δ* 3.20) with α-oriented H-5 (*δ* 1.53), H-9 (*δ* 0.78), and H-14 (*δ* 1.41), suggesting the β-orientation of OH at C-7 (Fig. 4).

**Table 1:** ^1^H NMR data of 2–5, and 7 (*δ* in ppm, *J* in Hz)

No.	1^a^	2^b^	3^b^	4^b^	5^a^	7^a^
1	1.05 t (12.8)	1.01 t (13.0)	1.07 t (13.0)	1.04 t (13.0)	0.70 t (12.8)	1.60 (o), 1.68 (o)
	2.10 dd (12.8, 6.0)	1.99 (o^c^)	1.99 dd (13.0, 6.0)	1.98 dd (13.0, 6.0)	1.62 (o)	
2	2.56 dp (12.8, 6.4)	2.57 dp (13.0, 6.5)	2.56 dp (13.0, 6.5)	2.51 dp (13.0.6.0)	1.57 (o)	2.58 dp (12.8, 6.4)
3	—	—	—	—	3.10 (o)	—
4	1.98 dd (14.0, 3.6)	2.01 (o)	1.93 dd (14.0, 3.0)	2.14 dd (14.5, 3.5)	1.58 (o), 1.94 (o)	2.00 dd (14.0,3.6)
	2.42 t (14.0)	2.94 t (14.0)	2.88 t (14.0)	2.95 t (14.5)		2.84 t (14.4)
5	1.49 (o)	1.53 (o)	1.90 (o)	1.73 (o)	1.30 (o)	2.14 dt (14.4, 3.6)
6	1.35 m, 1.38 m	3.54 dd (3.5, 2.5)	3.46 t (3.0)	3.77 t (3.0)	3.67 d (2.4)	3.64 q (2.4)
7	0.92 qd (12.4, 5.2)	3.20 dd (10.0, 3.5)	3.61 t (3.0)	—	—	1.50 (o), 1.59 (o)
	1.73 dq (13.2, 3.6)					
8	1.51 (o)	1.81 (o)	1.90 (o)	3.10 t (11.5)	3.11 (o)	2.19 td (12.0, 4.0)
9	0.72 td (11.4, 4.0)	0.78 (o)	1.23 (o)	1.10 (o)	1.00 td (11.4, 6.0)	—
11	1.44 (o), 1.63 (o)	1.50 (o), 1.66 (o)	1.50 (o), 1.64 (o)	1.68 (o)	1.63 (o)	1.59 (o), 1.85 (o)
12	1.29 (o), 1.53 (o)	1.30 (o), 1.53 (o)	1.28 (o), 1.51 (o)	1.26 (o), 1.53 (o)	1.23 (o), 1.53 (o)	1.31 (o), 1.68 (o)
14	1.23 (o)	1.41 (o)	1.71 (o)	1.71 (o)	1.70 (o)	1.82 (o)
15	1.26 (o), 1.56 (o)	1.66 (o), 1.91 (o)	1.31 (o), 1.70 (o)	1.18 (o), 2.05 (o)	1.14 q (8.0), 1.97 (o)	1.29 (o), 1.49 (o)
16	1.64 (o)	1.62 (o), 1.84 (o)	1.69 (o), 1.87 (o)	1.80 (o), 1.82 (o)	1.66 (o), 1.83 (o)	1.66 (o), 1.85 (o)
	1.83 dt (12.0, 3.2)					
18	0.86 s	0.89 s	0.89, s	0.91 s	0.87 s	0.90 s
19	1.12 s	1.27 s	1.25, s	1.50 s	1.27 s	1.37 s
20	0.96 d (6.4)	0.96 d (6.5)	0.96 d (6.5)	1.01 d (6.5)	0.96 d (6.0)	0.97 d (6.8)
21	1.17 s	1.18 s	1.21 s	1.23 s	1.20 s	1.23 s

a Measured in CD_3_OD at 400 MHz.

b Measured in CD_3_OD at 500 MHz.

c Overlapped.

**Table 2:** ^13^C NMR data of compounds 2–5, and 7 (CD_3_OD at 125 MHz for 2–4, and 100 MHz for 5 and 7)

No.	1	2	3	4	5	7
1	50.0	51.0	51.3	48.5	48.9	44.4
2	42.2	42.1	42.3	40.8	36.3	42.0
3	215.7	216.3	216.6	211.8	77.7	216.8
4	45.6	43.0	42.9	40.9	35.5	43.3
5	46.9	50.2	45.8	51.6	52.1	44.8
6	29.9	75.2	76.0	79.4	81.6	71.2
7	32.7	78.3	72.1	211.7	215.8	34.0
8	37.5	39.6	36.5	45.6	47.0	34.4
9	55.4	53.3	46.3	54.9	57.8	76.6
10	37.7	37.2	37.8	37.1	38.1	42.5
11	22.3	22.3	22.0	21.9	22.6	28.0
12	32.9	32.7	32.6	30.8	32.1	28.3
13	45.6	47.6	46.8	45.5	46.4	47.0
14	52.0	51.3	45.2	42.8	44.2	44.4
15	24.3	27.4	23.7	24.0	24.9	24.1
16	39.3	39.5	39.2	38.8	39.1	39.3
17	82.2	81.6	82.4	81.1	81.7	82.4
18	14.7	14.9	14.5	14.1	14.7	13.9
19	12.7	16.4	15.7	15.5	16.7	18.6
20	14.9	15.0	14.9	14.6	19.3	15.1
21	26.1	26.2	26.2	25.8	26.0	26.4

Compound 3 was obtained as a white crystalline solid (Fig. 1). The molecular formula of 3 was deduced to be C_21_H_34_O_4_ (M^+^*m*/*z* 350.2439, calcd 350.2457) from HREI-MS. Absorbances in the IR spectrum at 3372 and 1697 cm^−1^ were due to the presence of hydroxyl and carbonyl ketone groups, respectively. The spectral data of compound 3 was similar to compound 2. The only difference was the orientation of the OH group at C-7. NOESY spectrum displayed a correlation between H-7 (*δ* 3.61) and β-oriented H-8 (*δ* 1.90), but not with α-oriented H-9 (*δ* 1.22) (Fig. 4). Small coupling constant (*J*) of H-7 (*J*_a/e_ = 3.0 Hz) in compound 3 also supported α-orientation of OH group, in comparison to coupling constant of H-7 (*J*_a/a_ = 10.5 Hz; *J*_a/e_ = 3.5 Hz) in compound 2.

Compound 4 was purified as a white solid (Fig. 1). The molecular composition (C_21_H_32_O_4_) was evaluated through HREI-MS analysis ([M]^+^ at *m*/*z* 348.2305, calcd for 348.2301). The IR absorbances at 3449 and 1700 cm^−1^ were due to the presence of hydroxyl and carbonyl groups, respectively. ^1^H NMR spectrum (Table 1) showed a new downfield signal at *δ* 3.77. In ^13^C NMR spectrum (DEPT-135°), a signal for an oxymethine carbon appeared at *δ* 79.4, which correlated with *δ* 3.77 in the HSQC spectrum. A new signal for a carbonyl group was also observed at *δ* 211.7 in the Broad-brand spectrum (Table 2). Signal at *δ* 3.77 showed HMBC correlations with C-4 (*δ* 40.90), C-5 (*δ* 51.6), C-7 (*δ* 211.7), C-8 (*δ* 45.6), and C-10 (*δ* 37.1), indicating hydroxylation at C-6 (*δ* 79.4). The HMBC correlations of H-5 (*δ* 1.73), H-6 (*δ* 3.77), and H-8 (*δ* 3.10) with carbonyl carbon (*δ* 211.7) suggested the position of the carbonyl at C-7 (Fig. 3). H-6 (*δ* 3.77) exhibited NOESY correlations with β-oriented CH_3_-19 (*δ* 1.50) and H-8 (*δ* 3.10), suggesting the α-orientation of the hydroxyl group at C-6 (*δ* 79.4) (Fig. 4).

Compound 5 was isolated as a white crystalline solid (Fig. 1). The molecular formula of 5 was deduced to be C_21_H_34_O_4_ (M^+^*m*/*z* 350.2454 calcd 350.2457) from HREI-MS analysis. The hydroxyl (3456 cm^−1^) and carbonyl (1701 cm^−1^) groups were identified from the IR spectrum. ^1^H NMR spectrum (Table 1) showed two new downfield signals resonating at *δ* 3.10 and 3.67, which correlated with *δ* 77.7 and 81.6 in HSQC, respectively. Beside this, the ^13^C NMR spectrum displaced a signal for a new carbonyl carbon at *δ* 215.8 (Table 2). A hydroxyl group was deduced at C-6 through HMBC correlations of *δ* 3.67 with C-4 (*δ* 35.5), C-5 (*δ* 52.1), C-7 (*δ* 215.8), C-8 (*δ* 47.0), and C-10 (*δ* 38.1), and also confirmed by COSY correlations with H-5 (*δ* 1.30) (Fig. 3). The HMBC interactions of H-6 (*δ* 3.67) and H-8 (*δ* 3.11) with *δ* 215.8 indicated carbonyl carbon at C-7. The HMBC correlations of *δ* 77.7 with H_3_-20 (*δ* 0.96), H-1 (*δ* 0.70, 1.62), H-2 (1.57), and H-4 (1.94, 1.58) suggested the position of another hydroxyl group at C-3 (*δ* 77.7). This was further supported by COSY cross-peaks of H-3 (*δ* 3.10) with H-2 (*δ* 1.57) and H-4 (*δ* 1.94, 1.58). The NOESY correlations of H-3 (*δ* 3.10) with α-oriented H-5 (*δ* 1.30) suggested the β-orientation of hydroxyl group at C-3. β-orientation of hydroxyl group at C-6 (*δ* 81.6) was deduced through NOESY correlation of H-6 (*δ* 3.67) with α-oriented H-5 (*δ* 1.30). There was no any NOESY interaction of H-6 with β-oriented H-8 (*δ* 3.11) and H_3_-19 (*δ* 1.27) (Fig. 4).

The stucture of 6 was determined after comparing the data reported (Ahmad, M. S *et al.*, 2017). It was a transformed product through *Cunninghamella blakesleeana* from methasterone. Herein, the same product was obtained by *Macrophomina phaseolina.*

**Fig. 2:**
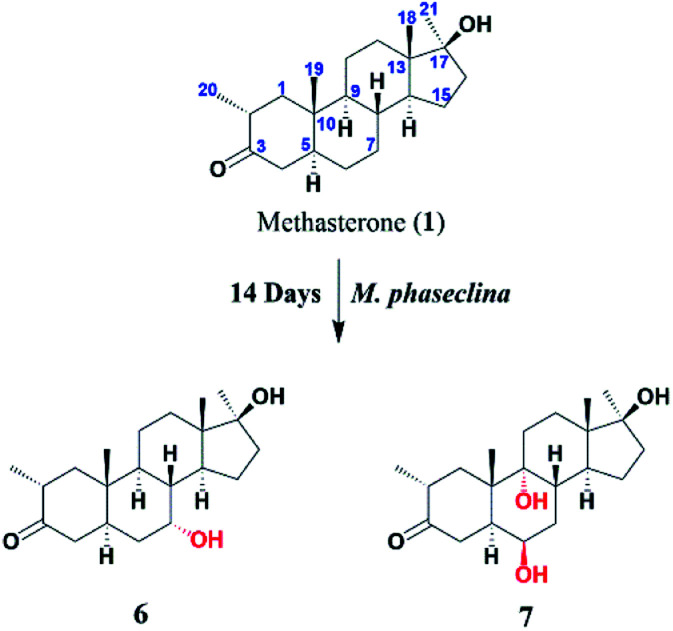
Biotransformation of methasterone (1) with *Cunninghamella blakesleeana*.

**Fig. 3:**
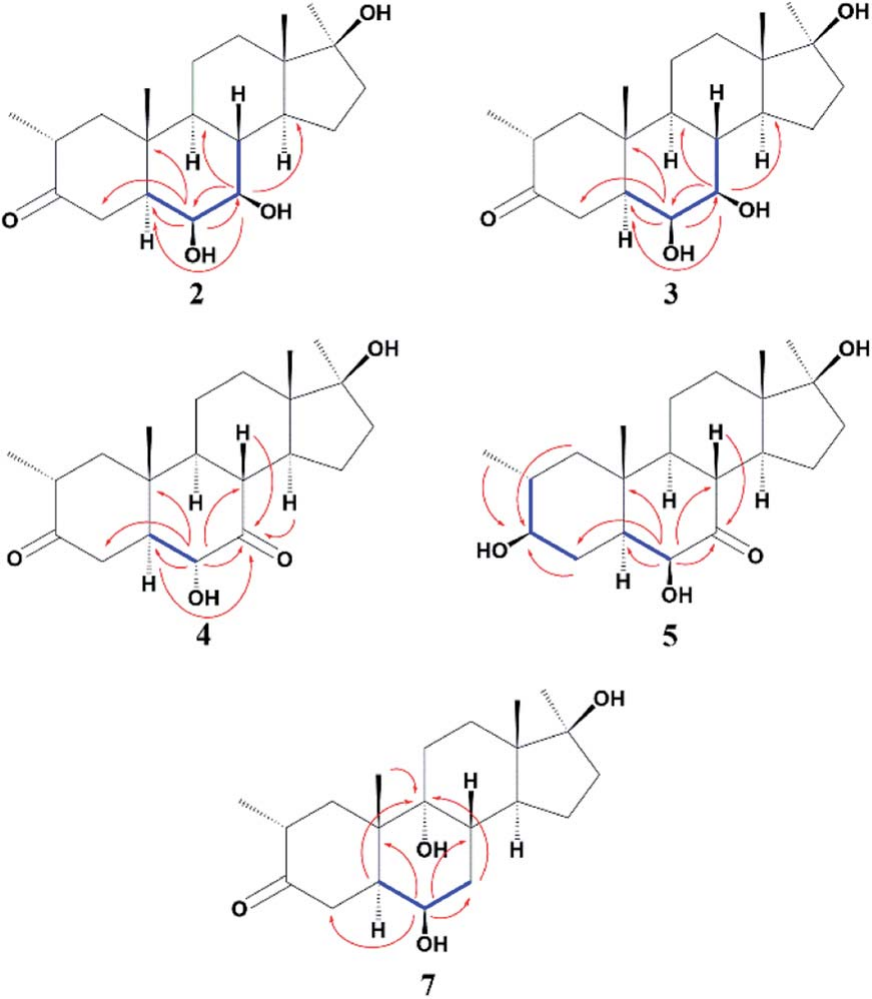
COSY and HMBC correlations of compounds 2–5 and 7.

Compound 7 was isolated as a white crystalline solid (Fig. 2). The HREI-MS revealed the [M]^+^ at *m*/*z* 350.2434 (C_21_H_34_O_4_, calcd 350.2457), which indicated the di-hydroxylation of substrate 1. In the IR spectrum, absorbances at 3454 and 1697 cm^−1^ were due to the presence of hydroxyl and carbonyl groups, respectively. In the ^1^H NMR spectrum (Table 1), a new downfield signal was resonated at *δ* 3.64, whereas the ^13^C NMR spectrum revealed two new downfield signals at *δ* 71.2 (methine carbon) and 76.6 (quaternary carbon) (Table 2). An OH group at C-6 (*δ* 71.2) was inferred from HMBC correlations of H-6 (*δ* 3.64) with C-4 (*δ* 43.3), C-7 (*δ* 34.0), C-8 (*δ* 34.4), and C-10 (*δ* 42.5). This was further supported by COSY correlations of H-6 (*δ* 3.64) with H-5 (*δ* 2.14) and H-7 (*δ* 1.50, 1.59) (Fig. 3). Second OH group at C-9 (*δ* 76.6) was deduced by HMBC correlations of H_3_-19 (*δ* 1.37) with H-5 (*δ* 2.14), H-7 (*δ* 1.50, 1.59), H-11 (*δ* 1.59, 1.85), and H-12 (*δ* 1.31, 1.68). The NOESY spectrum showed a correlation of H-6 (*δ* 3.64) with α-oriented H-5 (*δ* 2.14), which suggested the β-orientation of OH group at C-6 (*δ* 71.2). CH_3_-19 (*δ* 1.37) owned a correlation with H-11 (*δ* 1.85), indicative of the same *trans*-junction between C-8 and C-9. Therefore, the 9-OH remained the same α-orientation as the substrate 1.

**Fig. 4:**
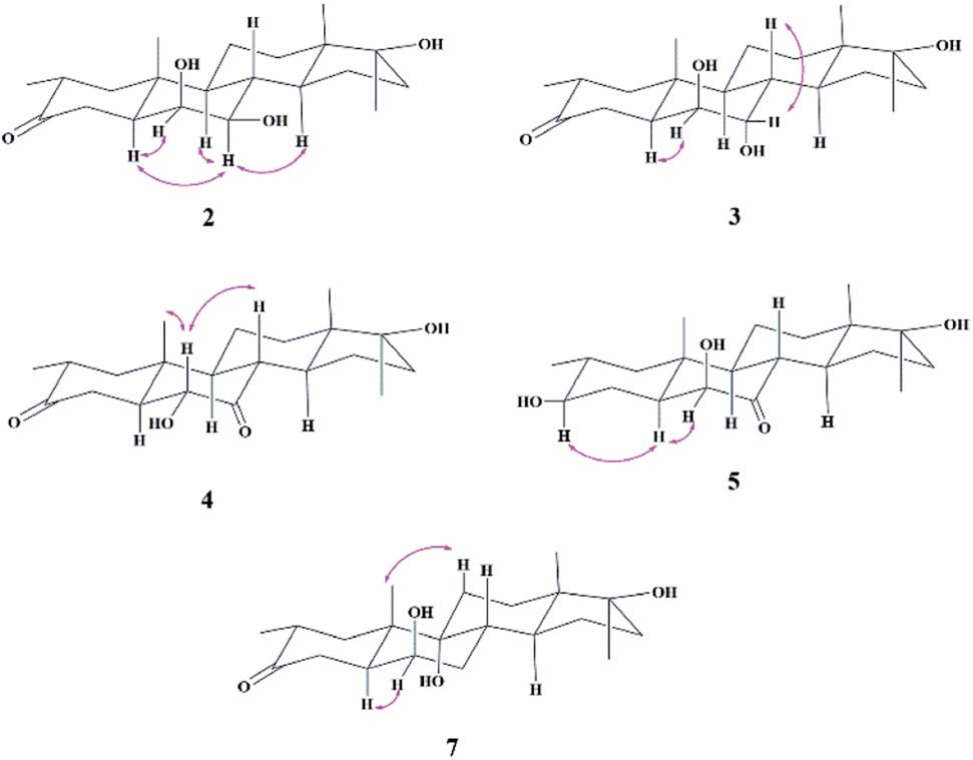
NOESY correlations of compounds 2–5 and 7.

Anti-inflammatory activity of all compounds (2–7) was evaluated using a NO production inhibitory assay. Substrate 1 was found to be a potent anti-inflammatory agent against nitric oxide (NO) production with an IC_50_ of 23.9 ± 0.2 μg mL^−1^, when compared to standard LNMMA having an IC_50_ = 24.2 ± 0.8 μg mL^−1^. While metabolites 2 (38.1 ± 0.5 μg mL^−1^), and 7 (40.2 ± 3.3 μg mL^−1^) exhibited moderate effect on NO production.

Cytotoxicity was determined against MCF-7 (breast cancer), NCI-H460 (lung cancer), HeLa (cervical cancer), and BJ (normal human fibroblast) cell lines. The results revealed that compound 1 was found to be cytotoxic against human normal cell line (BJ) with an IC_50_ of 8.01 ± 0.52 μg mL^−1^, but other transformed products (2–7) were found to be non-cytotoxic. Compounds 1–7 were non-cytotoxic against MCF-7, NCI-H460, and HeLa cancer cell lines (having <50% inhibition at 50 μM), compared with the positive control doxorubicin having IC_50_ of 1.2 ± 0.11 μg mL^−1^ against MCF-7, 0.76 ± 0.05 μg mL^−1^ against NCI-H460, and 0.16 ± 0.01 μg mL^−1^ against HeLa cell line. It is concluded that the anti-inflammatory activity of transformed products was reduced to a certain level, the cytotoxicity was also reduced. Biotransformation was found to be an effective technique for the production of active and safe lead metabolites for the exploration of new drugs.

## Experimental

### General protocol

Normal silica gel (100–200 mesh) was used for the initial fractionation using column chromatography (CC). Final purification of compounds was implemented by reverse phase preparative recycling HPLC (Japan Analytical Industry Co., Ltd) on the ODS-H-80 column. Thin-layer chromatography (TLC) was used for the analysis of the compound through pre-coated cards (Silica gel 60 F_254_, Merck, Germany). The NMR spectrum was recorded on a Bruker Advance spectrometer of 400, 500, and 600 MHz in methanol (deuterated). EI-MS and HREI-MS was performed on JEOL JMS-600H (Japan), and JEOL JMS HX-110, respectively. UV (Ultraviolet) spectra were recorded in methanol on Shimadzu UV 240 spectrophotometer (Tokyo, Japan). IR (Infrared) absorbance was recorded on an FTIR-8900 spectrophotometer as a KBr disk. Melting points were determined by Buchi 535 apparatus.

### Microorganisms and preparation of medium

*Cunninghamella blakesleeana* and *Macrophomina phaseolina* (KUCC 730) were acquired from American Type Culture Collection (ATCC) and Karachi University Culture Collection (KUCC). Sabouraud dextrose agar (Agar slant) was used to grow the fungi at 25 °C, and maintained at 4 °C.

The media for *C. blakesleeana*, and *M. phaseolina* was prepared by adding peptone (5.0 g), potassium dihydrogen phosphate (KH_2_PO_4_) (5.0 g), yeast extract (5.0 g), sodium chloride (NaCl) (5.0 g), glycerol (10 mL), and glucose (10.0 g) in one litter of distilled water.

### General fermentation protocol

Previously, Otten & Rosazza reported the general fermentation procedure used during biotransformation studies.^34^ In small scale screening, liquid media of 400 mL was prepared for each fungus. Fermentation media was shifted among four 250 mL conical flasks (100 mL in each), and then autoclaved at 120 °C for sterilization. Out of four flasks, two flasks were arranged as positive (substrate + fermentation media), one flask as negative (mature fungal growth in media), and one flask used as a seed flask. The fungus spores were added into each flask under sterilized conditions and kept for 3 to 4 days on an incubator shaker (128 rpm) at 24 ± 2 °C. After the mature growth of fungi, the substrate (20 mg for each positive flask) was dissolved in methanol and transferred to the fungus culture. All flasks were again put on a shaker for fermentation. After the definite incubation period (7 days and 14 days for two flasks, respectively), the process of fermentation was stopped by adding ethyl acetate. The sporangia were separated by filtration and extracted with ethyl acetate 3–4 times. The organic phase was isolated by a separation funnel, and then concentrated on a rotatory evaporator. The extract was analyzed by TLC.

### Biotransformation with *Cunninghamella blakesleeana*

Based on the small scale screening, 10 L media was prepared by mixing the above-mentioned components in water for *Cunninghamella blakesleeana* growth. It was dispersed among 6 conical flasks (3 L size with 1.5 L media in each), and then autoclaved at 120 °C for sterilization of media. The media was cooled down until room temperature and then incubated with seeds of *C*. *blakesleeana* under pasteurized conditions. The conical flasks were kept on a shaker (128 rpm) at 24 ± 2 °C for 3–5 days for fungi growth. After the mature growth, 1.0 g of methasterone (1) was dissolved in methanol (9 mL), then distributed to 6 flasks, and again placed on a shaker (128 rpm) at 24 ± 2 °C for 14 days. Then ethyl acetate was added to stop the fermentation, and biomass was filtered. The filtrate was extracted thrice using ethyl acetate, and then the organic phase was concentrated on a high vacuum rotary evaporator. The gummy crude extract (2.0 g) was obtained and then subjected to silica gel CC through gradient elution with hexane/acetone by increasing polarity (5–100% acetone). The fractions were combined based on TLC analysis, and three major fractions (Fr.2–Fr.4) were obtained. Finally, the purification was performed on recycling semi-preparative RP-HPLC (ODS-H80, MeOH : H_2_O 70 : 30). Metabolite 2 (*R*_t_ = 33 min) (5.4 mg), 3 (*R*_t_ = 34 min) (5.3 mg), 4 (*R*_t_ = 32 min) (5.1 mg), 5 (*R*_t_ = 39 min) (5.3 mg) were purified from fraction Fr.2–Fr.4.

#### 6β,7β,17β-Trihydroxy-2α,17α-dimethyl-5α-androstane-3-one (2)

White solid; mp 139–141 °C; [*α*]^27^_D_ = +12 (*c* 0.015 CH_3_OH); IR (KBr disk) *ν*_max_ (cm^−1^): 3418 (OH), 2943, 2867 (CH_2_, CH_3_); 1703 (C=O); EI-MS *m*/*z* (rel. int., %): 350.3 [M]^+^ (3.5), 332.2 (100.0), 314.2 (80.5), 299.2 (48.7), 281.2 (23.3), 261.2 (58.9), 243.2 (39.6), 229.2 (24.9), 175.1 (31.4), 159.1 (24.2), 147.1 (24.1). HREI-MS *m*/*z* calcd for C_21_H_34_O_4_ 350.2457 [M]^+^, found 350.2458; ^1^H NMR (CD_3_OD, 500 MHz) Table 1; ^13^C NMR (CD_3_OD, 125 MHz) Table 2.

#### 6β,7α,17β-Trihydroxy-2α,17α-dimethyl-5α-androstane-3-one (3)

White solid; mp 143–145 °C; [*α*]^27^_D_ = −19 (*c* 0.01 CH_3_OH); IR (KBr disk) *ν*_max_ (cm^−1^): 3372 (OH), 2947, 2871 (CH_2_, CH_3_); 1697 (C=O); EI-MS *m*/*z* (rel. int., %): 351.2 [M + H]^+^ (5.1), 332.2 (100.0), 314.2 (50.5), 299.2 (27.4), 274.2 (56.3), 261.2 (94.8), 256.2 (27.1), 243.2 (25.0), 208.2 (23.8), 177.2 (22.9), 159.2 (18.3). HREI-MS *m*/*z*: calcd for C_21_H_34_O_4_ 350.2457 [M]^+^, found 350.2439; ^1^H NMR (CD_3_OD, 500 MHz) Table 1; ^13^C NMR (CD_3_OD, 125 MHz) Table 2.

#### 6α,17β-Dihydroxy-2α,17α-dimethyl-5α-androstane-3,7-dione (4)

White solid; mp 115–117 °C; [*α*]^27^_D_ = −70 (*c* 0.006 CH_3_OH); IR (KBr disk) *ν*_max_ (cm^−1^): 3449 (OH), 2968, 2909 (CH_2_, CH_3_); 1700 (C=O); EI-MS *m*/*z* (rel. int., %): 348.2 [M]^+^ (66.3), 330.2 (100.0), 315.1 (32.2), 287.1 (27.8), 273.1 (23.1), 259.1 (32.2), 222.1 (30.1), 191.1 (32.4), 175.1 (10.4), 161.1 (13.7), 135.1 (16.5); HREI-MS *m*/*z* calcd for C_21_H_32_O_4_ 348.2301[M]^+^, found 348.2305; ^1^H NMR (CD_3_OD, 400 MHz) Table 1; ^13^C NMR (CD_3_OD, 100 MHz) Table 2.

#### 3β,6β,17β-Trihydroxy-2α,17α-dimethyl-5α-androstane-7-one (5)

White solid; mp 175–177 °C; [*α*]^27^_D_ = −53 (*c* 0.01 CH_3_OH); IR (KBr disk) *ν*_max_ (cm^−1^): 3456 (OH), 2936, 2876 (CH_2_, CH_3_); 1701 (C=O); EI-MS *m*/*z* (rel. int., %): 350.3 [M]^+^ (74.5), 332.3 (100.0), 317.2 (31.7), 299.2 (24.2), 275.2 (21.3), 261.2 (21.5), 224.2 (30.2), 191.1 (33.5), 177.1 (17.7), 149.1 (20.9), 121.1 (26.1); HREI-MS *m*/*z* calcd for C_21_H_34_O_4_ 350.2457 [M]^+^, found 350.2454; ^1^H NMR (CD_3_OD, 400 MHz) Table 1; ^13^C NMR (CD_3_OD, 100 MHz) Table 2.

### Biotransformation with *Macrophomina phaseolina*

9 L media for *Macrophomina phaseolina* was prepared, and distributed to six 3 L conical flasks (1.5 L in each), following with sterilization and inoculation. After full growth of the fungus, 1.0 g methasterone (1) was dissolved in methanol (9.0 mL), and equally distributed into the six culture flasks (1.5 mL in each). After 14 days, the culture was extracted and 3 g crude extract was obtained. The extract was then loaded to normal silica gel CC with hexane-acetone by increasing percentage of acetone (5–100%). Gained fractions were combined on the basis of TLC, and final purification was achieved by passing through recycling semi-preparative RP-HPLC (ODS-H80, MeOH : H_2_O; 70 : 30). Metabolite 6 (*R*_t_ = 33 min) (4.4 mg), and 7 (*R*_t_ = 36 min) (4.1 mg), respectively.

#### 6β,9α,17β-Trihydroxy-2α,17α-dimethyl-5α-androstane-3-one (7)

White solid; mp 139–141 °C; [*α*]^27^_D_ = −31 (*c* 0.007 CH_3_OH); IR (KBr disk) *ν*_max_ (cm^−1^): 3454 (OH), 2942, 2869 (CH_2_, CH_3_), 1697 (C=O); EI-MS *m*/*z* (rel. int., %): 350.3 [M]^+^ (7.1), 332.2 (76.2), 314.2 (69.9), 299.3 (40.0), 274.1 (100.0), 261.2 (27.4), 243.2 (33.3), 225.2 (43.9), 195.1 (41.5), 150.1 (47.6), 138.1 (55.3); HREI-MS *m*/*z* calcd for C_21_H_34_O_4_ 350.2457 [M]^+^, found 350.2434; ^1^H NMR (CD_3_OD, 400 MHz) Table 1; ^13^C NMR (CD_3_OD, 100 MHz) Table 2.

### Nitric oxide (NO) production inhibitory assay

The J774.2 cell line (mouse macrophage) was obtained from (European Collection of Cell Cultures, Salisbury, UK). It was cultivated in 75 cm^3^ flasks (IWAKI, Asahi Techno Glass, Shizuoka, Japan), containing DMEM (Sigma-Aldrich, Steinheim, Germany) and fetal bovine serum (FBS) (10%) (GIBCO, New York, NY, USA) and 1% streptomycin/penicillin, and were incubated in 5% CO_2_ at 37 °C. Fully-grown cells (10^6^ cells per mL) were transferred into the 96-well plate, and NO synthase was added into the wells by *E. coli* (30 μg mL^−1^) lipopolysaccharide (LPS) (DIFCO Laboratories, Detroit, MI, USA) for NO production. Samples were tested with different concentrations (1, 10, and 25 μg mL^−1^), and placed in CO_2_ (5%) at 37 °C. N^G^ monomethyl l-arginine acetate (LNMMA) was used as a standard NO inhibitor. Griess method was used to analyze the accumulation of nitrite in the cells.^35^ IC_50_ values were calculated by a Microsoft Excel-based formula.

### Cytotoxicity assay

MCF-7 (breast cancer), NCI-H460 (lung cancer), HeLa (cervical cancer) cell lines, and BJ (normal human fibroblast) cell lines were collected from cell culture biobank (PCMD, ICCBS). DMEM medium was used for MCF-7, HeLa, and BJ, and RPMI (ATCC modified medium) was used for NCI-H460. The media was supplemented with 10% FBS, 100 IU mL^−1^ of penicillin, and 100 μg mL^−1^ of streptomycin, and kept at 37 °C in a 5% CO_2_ incubator. The inhibition was investigated by MTT assay. Briefly, 100 μL per well of cell solutions (10 × 10^4^ cells per mL MCF-7 cells, 5 × 10^4^ cells per mL NCI-H460 cells, 6 × 10^4^ cells per mL HeLa cells, or 5 × 10^4^ cells per mL BJ cells), were added into 96-well plate and incubated for 24 h at 37 °C, before treatment with 6.25–50 μM of compounds or positive control doxorubicin for 48 h. After this, 20 μL of MTT (5 mg mL^−1^) was added to each well and then incubated for 4 h at 37 °C. Then 100 μL DMSO was added to each well to dissolve the purple formazan crystal. The absorbance was analyzed at 570 nm (MCF-7, NCI-H460, and HeLa) or 550 nm (BJ) using an ELISA plate reader. The IC_50_ value of active compounds (inhibition > 50% at 50 μM) was calculated using the EZ-Fit. The unit of μM was finally converted to μg mL^−1^ to be comparable with anti-inflammatory activity.

## Conclusion

In conclusion, methasterone (1) was effectively transformed by two fungi, *C. blakesleeana* and *M. phaseolina,* which led to the isolation and elucidation of seven transformed products (2–7). Oxidation and hydroxylation were the main routes of transformation. In comparison to standard LNMMA (IC_50_ = 24.2 ± 0.8 μg mL^−1^), substrate 1 (IC_50_ = 23.9 ± 0.2 μg mL^−1^) was found to be a potent anti-inflammatory agent against nitric oxide (NO) production. Metabolites 2 and 7 were moderately active with IC_50_ values of 38.1–40.7 μg mL^−1^. Substrate 1 displayed cytotoxicity on the human normal cell line (BJ) with an IC_50_ of 8.01 ± 0.52 μg mL^−1^. While all its transformed products are non-cytotoxic. Although the effect of the analogues was not as strong as the parent molecule, the cytotoxicity of the products after the fungal transformation has been reduced significantly. Therefore, biotransformation is not only an effective approach to discover potent leads for new drug discovery, but also a reliable method to decrease the toxicity of bioactive molecules.

## Conflicts of interest

There are no conflicts to declare.

## Supplementary Material

RA-012-D2RA01396G-s001
