# Supplementary material for: Structural transformation of methasterone with Cunninghamella blakesleeana and Macrophomina phaseolina
Source: RSC Adv. 2022 Mar 25;12(15):9494–500. doi: 10.1039/d2ra01396g (PMC8985176; doi:10.1039/d2ra01396g)
Supplement: RA-012-D2RA01396G-s001 [file RA-012-D2RA01396G-s001.pdf]

Electronic Supplementary Material (ESI) for RSC Advances.
This journal is © The Royal Society of Chemistry 2022

Supplementary Information

Structural Transformation of Methasterone with *Cunninghamella blakesleeana* and *Macrophomina phaseolina*

Muhammad Aamer [a], Mahwish Siddiqui [a], Almas Jabeen [b], Rimsha Irshad [a],

Farooq-Ahmad Khan [a,c], Atia-tul-Wahab [b], M. Iqbal Choudhary [a,b,d,] *, Yan Wang [a,]*

[a] *H. E. J. Research Institute of Chemistry, International Center for Chemical and Biological Sciences, University of Karachi, Karachi-75270, Pakistan*

[b] *Dr. Panjwani Center for Molecular Medicine and Drug Research, International Center for Chemical and Biological Sciences, University of Karachi, Karachi-75270, Pakistan*

[c] *Third World Center (TWC) for Chemical Sciences, International Center for Chemical & Biological Sciences, University of Karachi-75270, Pakistan*

[d] *Department of Biochemistry, Faculty of Science, King Abdulaziz University, Jeddah-21589, Saudi Arabia*

*Corresponding author

yan.wang@iccs.edu (Y.W.); iqbal.choudhary@iccs.edu (M.I.C.)

Table of Contents

Figure S1. EI-MS spectrum of compound **1** 5

Figure S2. ^{1}H NMR spectrum-1 of compound **1** (400 MHz, CD_3OD) 6

Figure S3. ^{1}H NMR spectrum-2 of compound **1** (400 MHz, CD_3OD) 6

Figure S4. ^{13}C NMR spectrum-1 of compound **1** (100 MHz, CD_3OD) 7

Figure S5. ^{13}C NMR spectrum-2 of compound **1** (100 MHz, CD_3OD) 7

Figure S6. EI-MS spectrum of compound **2** 8

Figure S7. HREI-MS spectrum of compound **2** 8

Figure S8. IR spectrum of compound **2** 9

Figure S9. ^{1}H NMR spectrum-1 of compound **2** (500 MHz, CD_3OD) 9

Figure S10. ^{1}H NMR spectrum-2 of compound **2** (500 MHz, CD_3OD) 10

Figure S11. ^{13}C NMR spectrum-1 of compound **2** (125 MHz, CD_3OD) 10

Figure S12. ^{13}C NMR spectrum-2 of compound **2** (125 MHz, CD_3OD) 11

Figure S13. DEPT spectrum of compound **2** 11

Figure S14. ^{1}H-^{1}H COSY spectrum of compound **2** 12

Figure S15. HSQC spectrum of compound **2** 12

Figure S16. HMBC spectrum-1 of compound **2** 13

Figure S17. HMBC spectrum-2 of compound **2** 13

Figure S18. NOESY spectrum of compound **2** 14

Figure S19. EI-MS spectrum of compound **3** 14

Figure S20. HREI-MS spectrum of compound **3** 15

Figure S21. IR spectrum of compound **3** 15

Figure S22. ^{1}H NMR spectrum-1 of compound **3** (500 MHz, CD_3OD) 16

Figure S23. ^{1}H NMR spectrum-2 of compound **3** (500 MHz, CD_3OD) 16

Figure S24. ^{13}C NMR spectrum-1 of compound **3** (125 MHz, CD_3OD) 17

Figure S25. ^{13}C NMR spectrum-2 of compound **3** (125 MHz, CD_3OD) 17

Figure S26. DEPT spectrum of compound **3** 18

Figure S27. ^{1}H-^{1}H COSY spectrum of compound **3** 18

Figure S28. HSQC spectrum-1 of compound **3** 19

Figure S29. HSQC spectrum-2 of compound **3** 19

Figure S30. HMBC spectrum-1 of compound **3** 20

Figure S31. HMBC spectrum-2 of compound **3** 20

Figure S32. NOESY spectrum of compound **3** 21

Figure S33. EI-MS spectrum of compound **4** 21
Figure S34. HREI-MS spectrum of compound **4** 22
Figure S35. IR spectrum of compound **4** 22
Figure S36. ^{1}H NMR spectrum-1 of compound **4** (500 MHz, CD_3OD) 23
Figure S37. ^{1}H NMR spectrum-2 of compound **4** (500 MHz, CD_3OD) 23
Figure S38. ^{1}H NMR spectrum-3 of compound **4** (500 MHz, CD_3OD) 24
Figure S39. ^{13}C NMR spectrum-1 of compound **4** (125 MHz, CD_3OD) 24
Figure S40. ^{13}C NMR spectrum-2 of compound **4** (125 MHz, CD_3OD) 25
Figure S41. DEPT spectrum of compound **4** 25
Figure S42. ^{1}H-^{1}H COSY spectrum of compound **4** 26
Figure S43. HSQC spectrum of compound **4** 26
Figure S44. HMBC spectrum-1 of compound **4** 27
Figure S45. HMBC spectrum-2 of compound **4** 27
Figure S46. NOESY spectrum of compound **4** 28
Figure S47. EI-MS spectrum of compound **5** 28
Figure S48. HREI-MS spectrum of compound **5** 29
Figure S49. IR spectrum of compound **5** 29
Figure S50. ^{1}H NMR spectrum-1 of compound **5** (400 MHz, CD3OD) 30
Figure S51. ^{1}H NMR spectrum-2 of compound **5** (400 MHz, CD3OD) 30
Figure S52. ^{13}C NMR spectrum-1 of compound **5** (100 MHz, CD3OD) 31
Figure S53. ^{13}C NMR spectrum-2 of compound **5** (100 MHz, CD3OD) 31
Figure S54. DEPT spectrum of compound **5** 32
Figure S55. ^{1}H-^{1}H COSY spectrum-1 of compound **5** 32
Figure S56. ^{1}H-^{1}H COSY spectrum-2 of compound **5** 33
Figure S57. HSQC spectrum-1 of compound **5** 33
Figure S58. HSQC spectrum-2 of compound **5** 34
Figure S59. HMBC spectrum-1 of compound **5** 34
Figure S60. HMBC spectrum-2 of compound **5** 35
Figure S61. NOESY spectrum-1 of compound **5** 35
Figure S62. NOESY spectrum-2 of compound **5** 36
Figure S63. EI-MS spectrum of compound **6** 36
Figure S64. ^{1}H NMR spectrum-1 of compound **6** (400 MHz, CD_3OD) 37
Figure S65. ^{1}H NMR spectrum-2 of compound **6** (400 MHz, CD_3OD) 37

Figure S66. ^{13}C NMR spectrum-1 of compound **6** (125 MHz, CD_3OD) 38
Figure S67. ^{13}C NMR spectrum-2 of compound **6** (125 MHz, CD_3OD) 38
Figure S68. EI-MS spectrum of compound **7** 39
Figure S69. HREI-MS spectrum of compound **7** 40
Figure S70. IR spectrum of compound **7** 41
Figure S71. ^{1}H NMR spectrum-1 of compound **7** (400 MHz, CD3OD) 41
Figure S72. ^{1}H NMR spectrum-2 of compound **7** (400 MHz, CD3OD) 42
Figure S73. ^{13}C NMR spectrum-1 of compound **7** (100 MHz, CD3OD) 42
Figure S74. ^{13}C NMR spectrum-2 of compound **7** (100 MHz, CD3OD) 43
Figure S75. DEPT spectrum of compound **7** 43
Figure S76. ^{1}H-^{1}H COSY spectrum of compound **7** 44
Figure S77. HSQC spectrum of compound **7** 44
Figure S78. HMBC spectrum-1 of compound **7** 45
Figure S79. HMBC spectrum-2 of compound **7** 45
Figure S80. NOESY spectrum of compound **7** 46

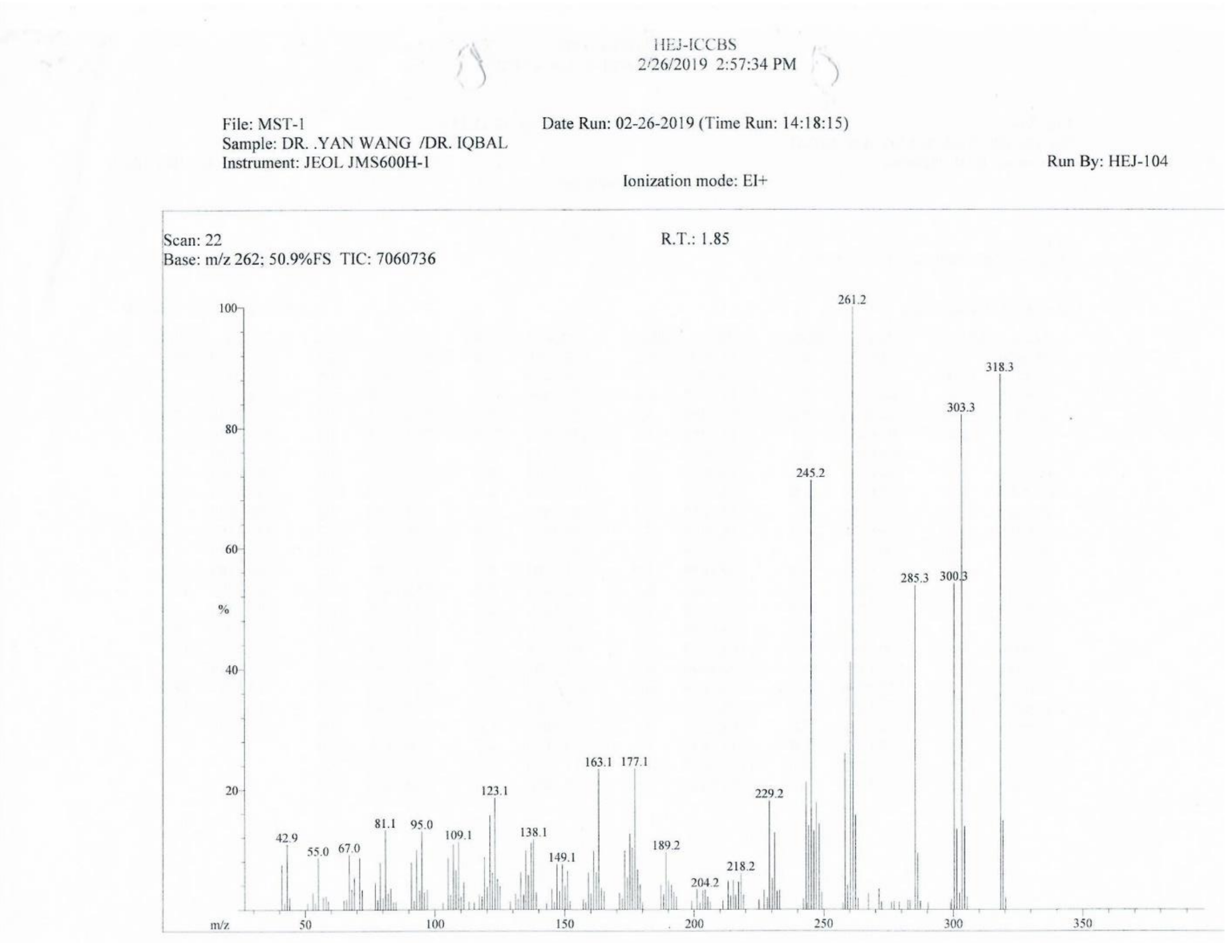

Figure S1. EI-MS spectrum of compound **1**

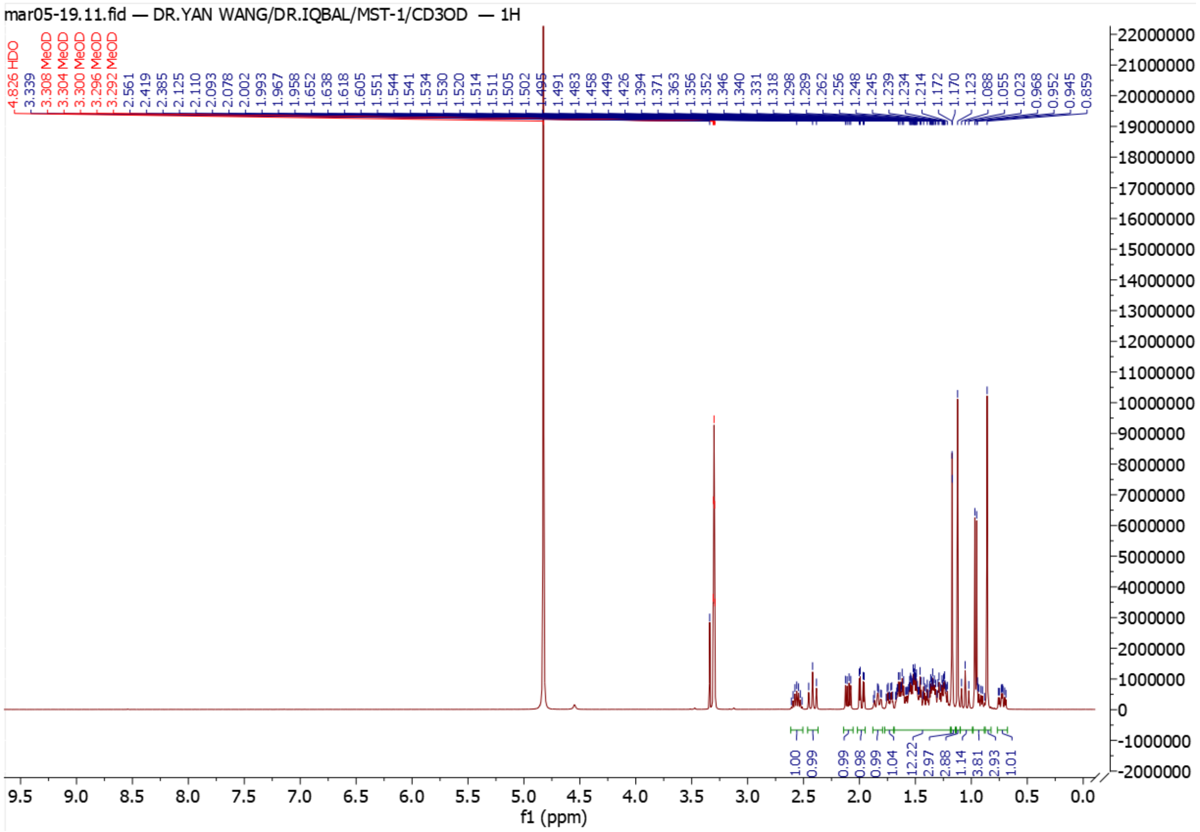

Figure S2. ^{1}H NMR spectrum-1 of compound **1** (400 MHz, CD_3OD)

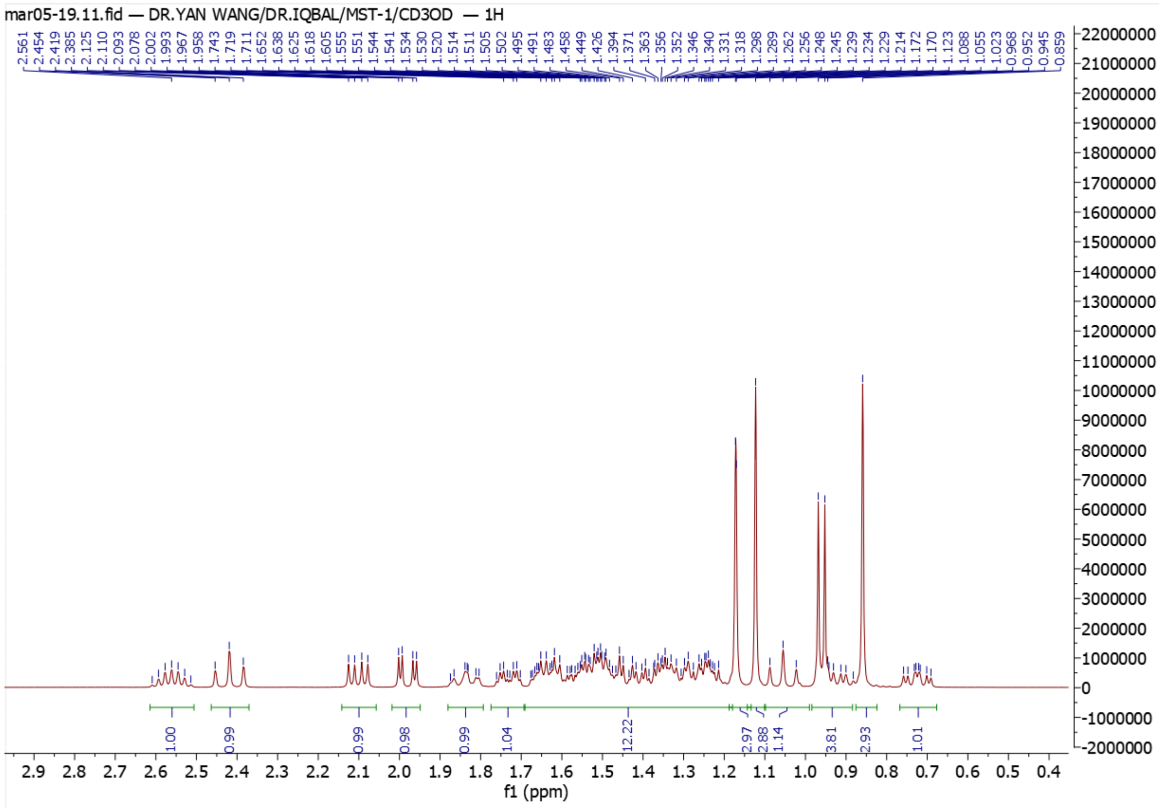

Figure S3. ^{1}H NMR spectrum-2 of compound **1** (400 MHz, CD_3OD)

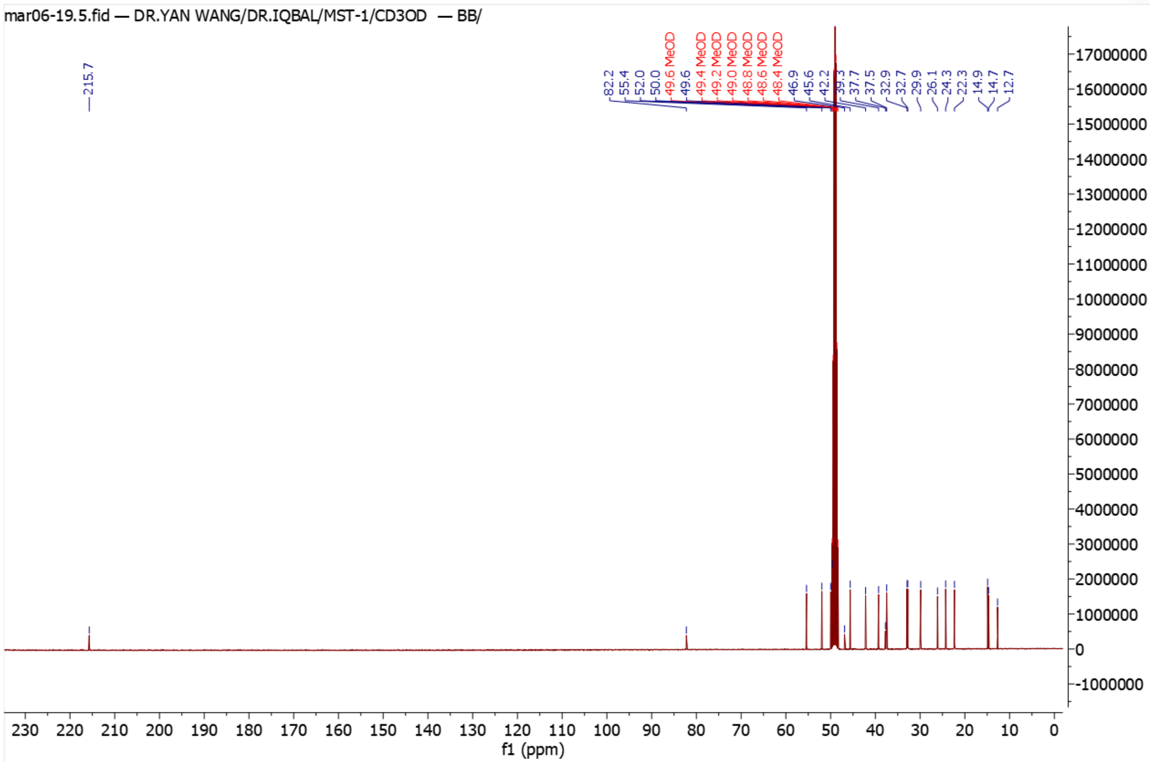

Figure S4. ^{13}C NMR spectrum-1 of compound **1** (100 MHz, CD_3OD)

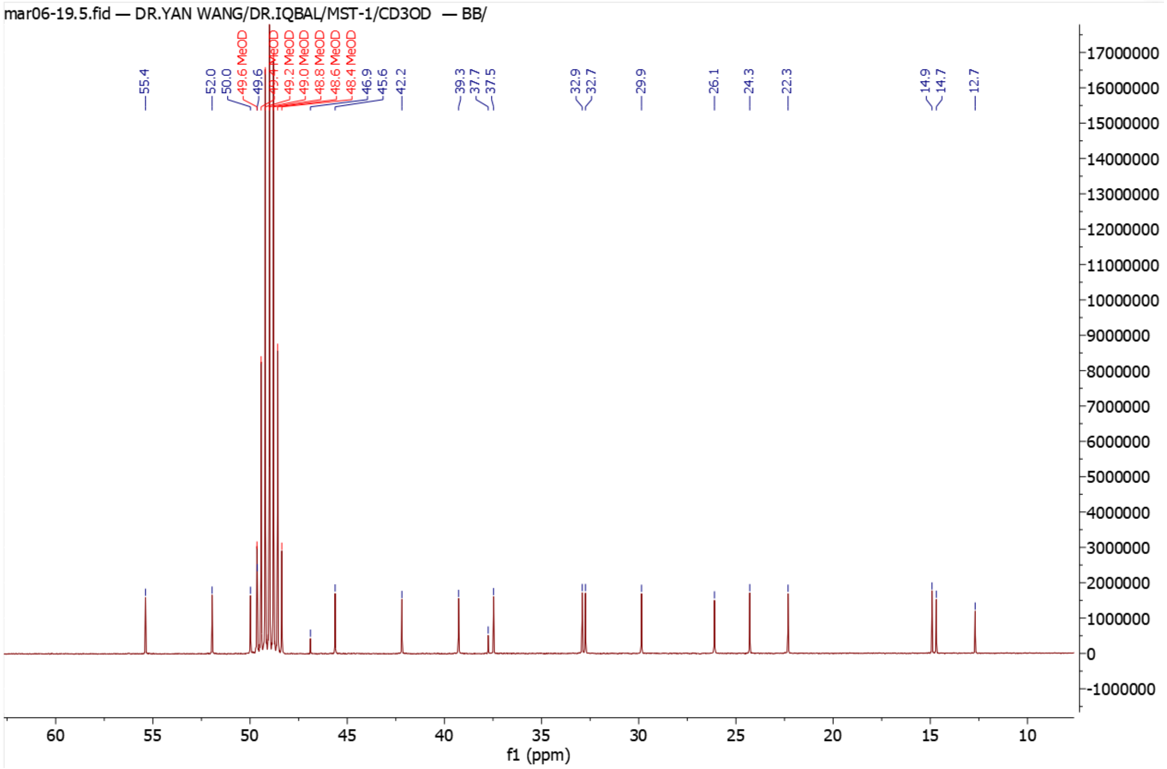

Figure S5. ^{13}C NMR spectrum-2 of compound **1** (100 MHz, CD_3OD)

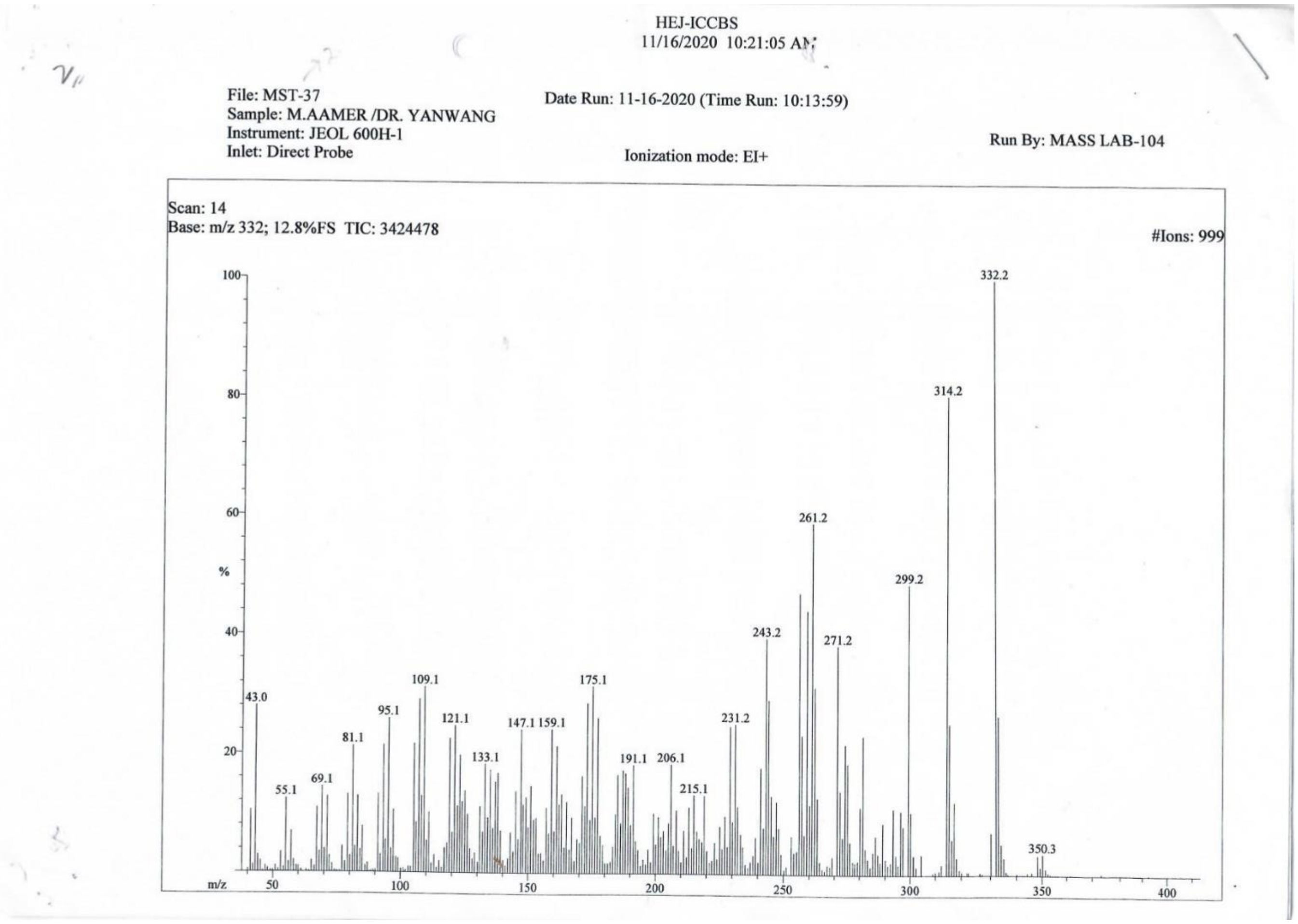

Figure S6. EI-MS spectrum of compound **2**

Mass	Relative Intensity	Theoretical Mass	Delta [ppm]	Delta [mmu]	RDB	Composition
		317.2328	-32.5	-10.3	1.5	$C_{17}H_{33}O_5$
		317.2117	34.1	10.8	6.5	$C_{20}H_{29}O_3$
318.2226	5.8	318.2195	9.9	3.1	6.0	$C_{20}H_{30}O_3$
319.2213	1.4	319.2273	-18.8	-6.0	5.5	$C_{20}H_{31}O_3$
		319.2121	29.0	9.2	1.5	$C_{16}H_{31}O_6$
328.2129	0.4	328.2191	-18.9	-6.2	12.0	$C_{25}H_{28}$
		328.2038	27.6	9.0	8.0	$C_{21}H_{28}O_3$
330.2158	3.3	330.2195	-11.2	-3.7	7.0	$C_{21}H_{30}O_3$
		330.2042	35.0	11.6	3.0	$C_{17}H_{30}O_6$
331.2244	1.4	331.2273	-8.8	-2.9	6.5	$C_{21}H_{31}O_3$
332.2309	100.0	332.2351	-12.7	-4.2	6.0	$C_{21}H_{32}O_3$
		332.2199	33.3	11.1	2.0	$C_{17}H_{32}O_6$
333.2349	25.4	333.2277	21.6	7.2	1.5	$C_{17}H_{33}O_6$
		333.2430	-24.2	-8.1	5.5	$C_{21}H_{33}O_3$
333.3495	0.3	333.3521	-8.0	-2.7	2.5	$C_{24}H_{45}$
333.3630	0.3	333.3521	32.6	10.9	2.5	$C_{24}H_{45}$
334.2415	3.6	334.2355	17.9	6.0	1.0	$C_{17}H_{34}O_6$
		334.2508	-27.8	-9.3	5.0	$C_{21}H_{34}O_3$
335.2239	2.8	335.2222	4.9	1.6	5.5	$C_{20}H_{31}O_4$
336.2248	0.7	336.2301	-15.7	-5.3	5.0	$C_{20}H_{32}O_4$
		336.2148	29.7	10.0	1.0	$C_{16}H_{32}O_7$
346.2202	0.5	346.2144	16.6	5.7	7.0	$C_{21}H_{30}O_4$
		346.2297	-27.5	-9.5	11.0	$C_{25}H_{30}O_1$
348.2285	1.3	348.2301	-4.6	-1.6	6.0	$C_{21}H_{32}O_4$
349.2216	0.6	349.2226	-3.1	-1.1	1.5	$C_{17}H_{33}O_7$
		349.2168	13.8	4.8	10.5	$C_{24}H_{29}O_2$
350.2458	2.2	350.2457	0.4	0.1	5.0	$C_{21}H_{34}O_4$
351.2543	0.5	351.2535	2.1	0.7	4.5	$C_{21}H_{35}O_4$

Activate Windows
Go to Settings to activat

mst-37

Figure S7. HREI-MS spectrum of compound **2**

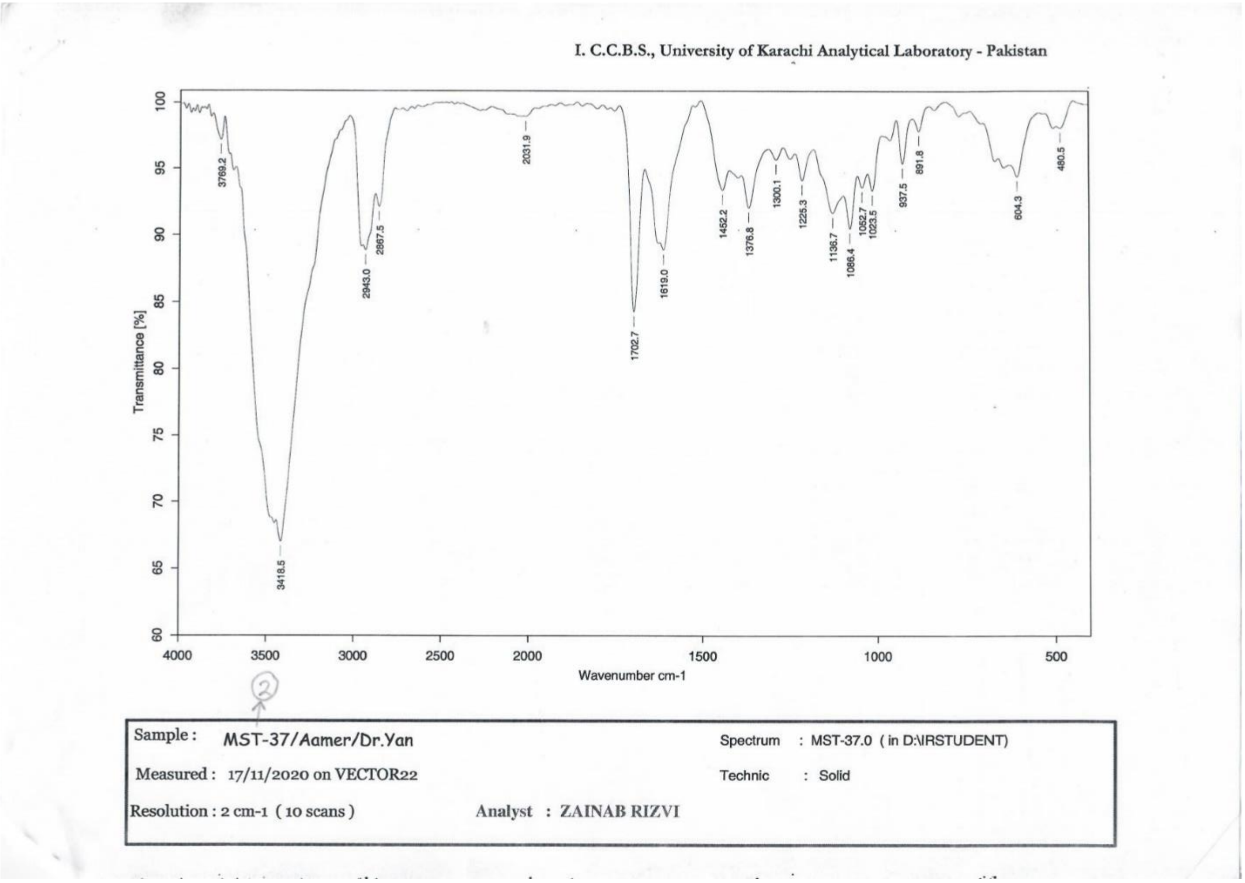

Figure S8. IR spectrum of compound **2**

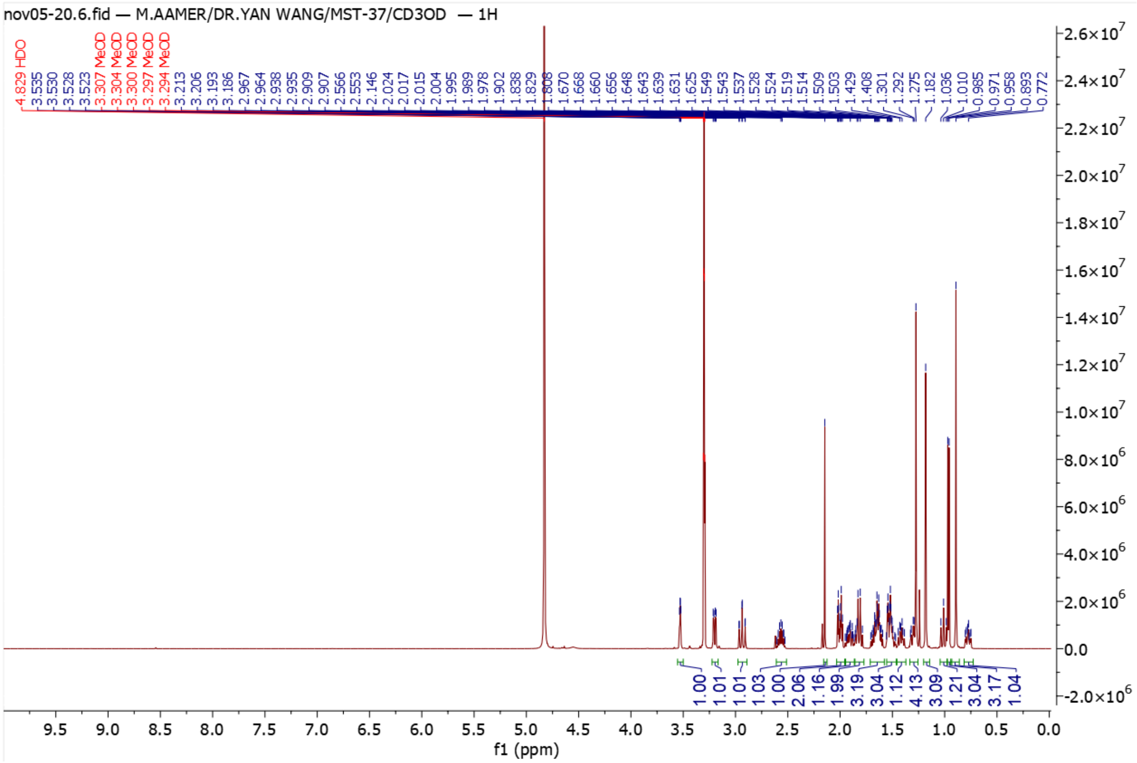

Figure S9. ^{1}H NMR spectrum-1 of compound **2** (500 MHz, CD_3OD)

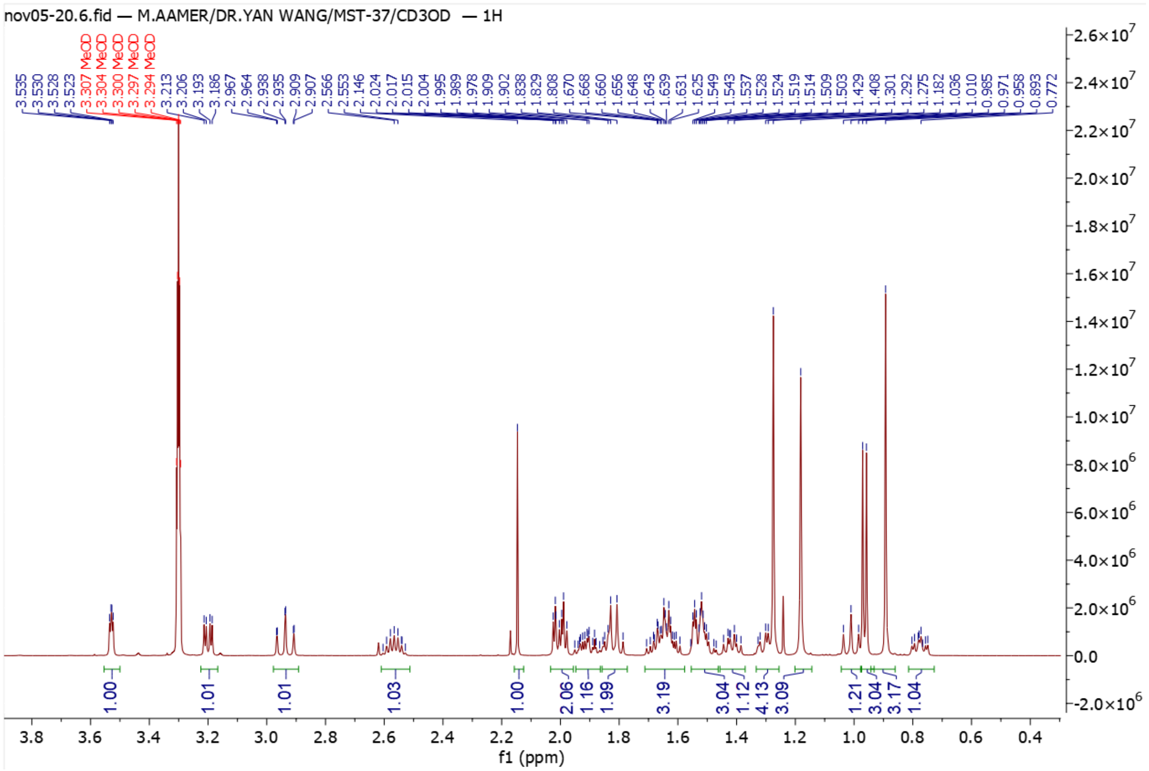

Figure S10. ^{1}H NMR spectrum-2 of compound **2** (500 MHz, CD_3OD)

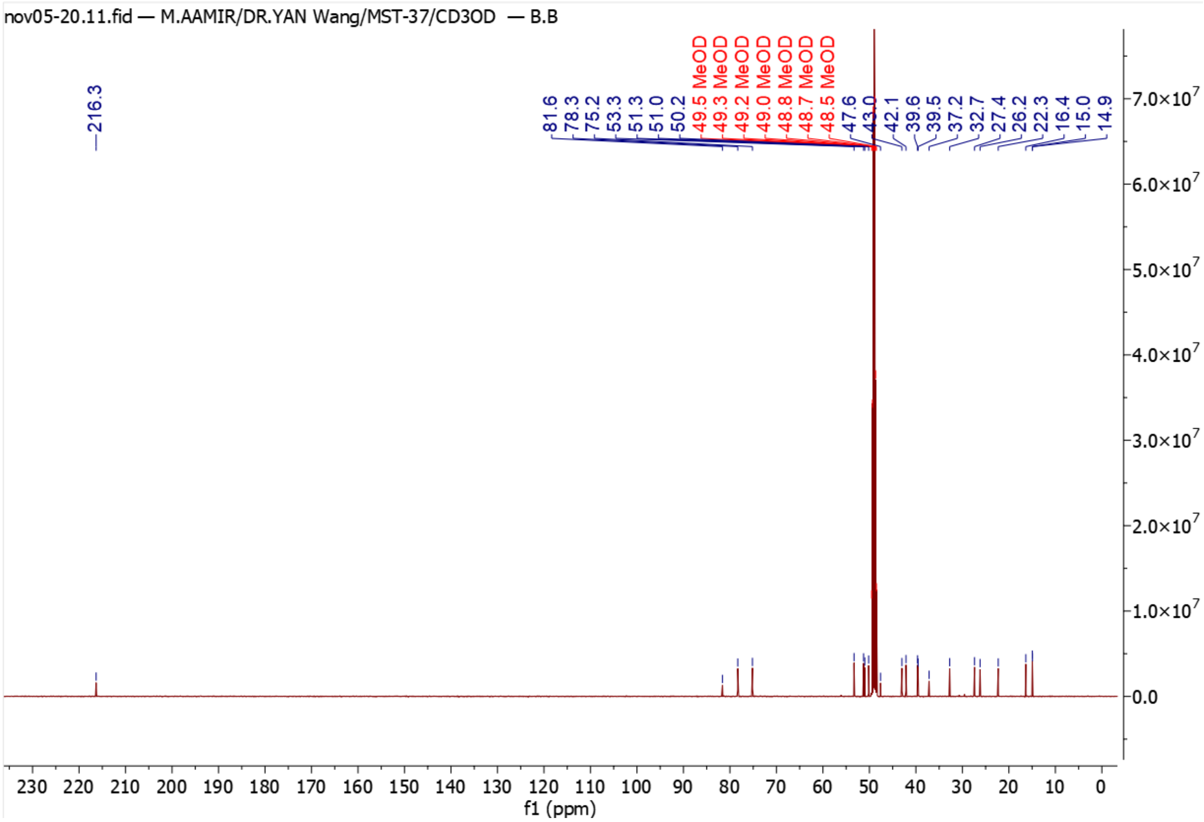

Figure S11. ^{13}C NMR spectrum-1 of compound **2** (125 MHz, CD_3OD)

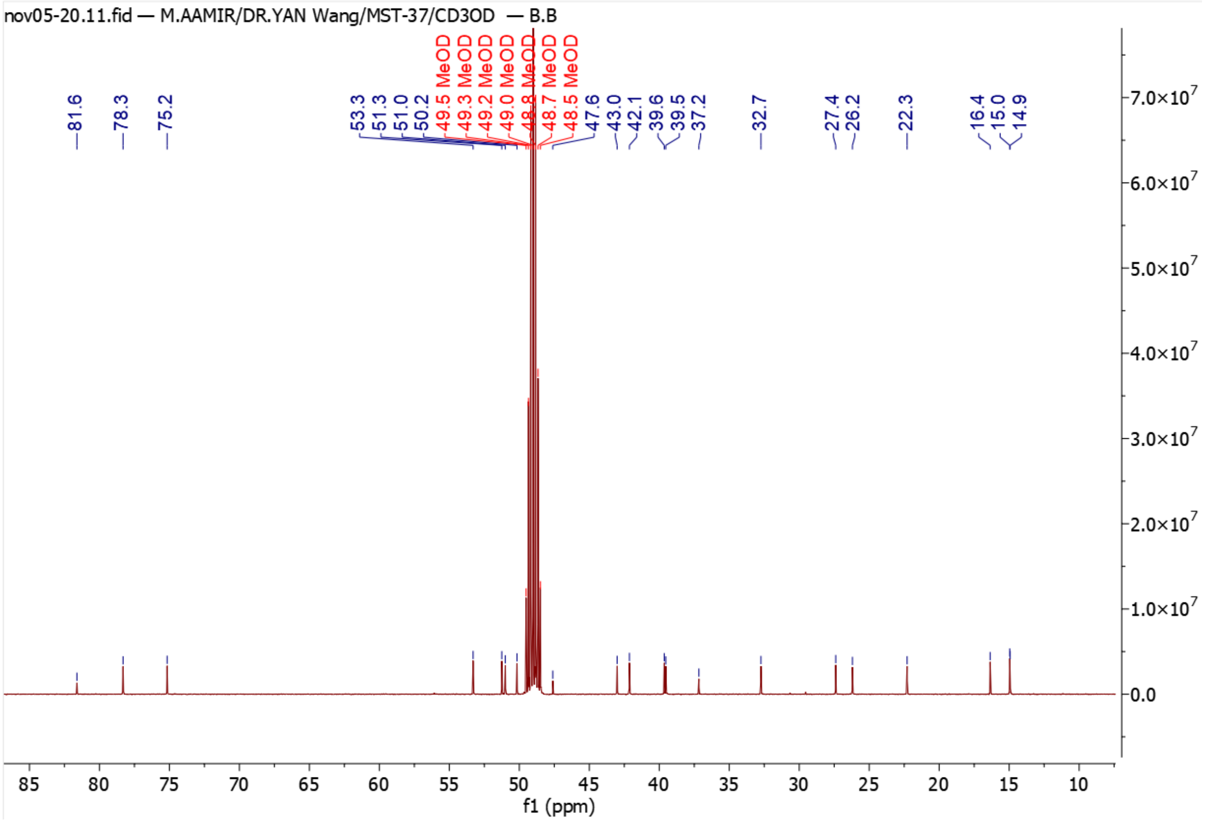

Figure S12. ^{13}C NMR spectrum-2 of compound **2** (125 MHz, CD_3OD)

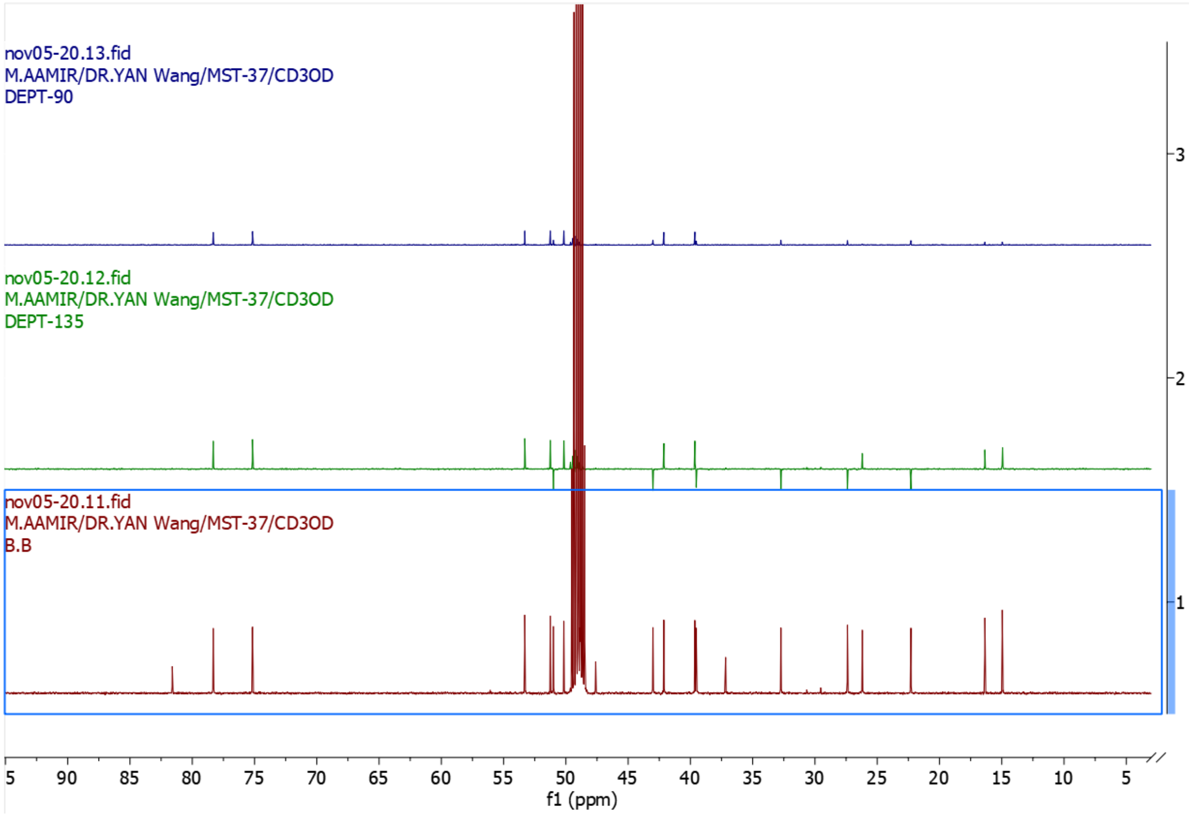

Figure S13. DEPT spectrum of compound **2**

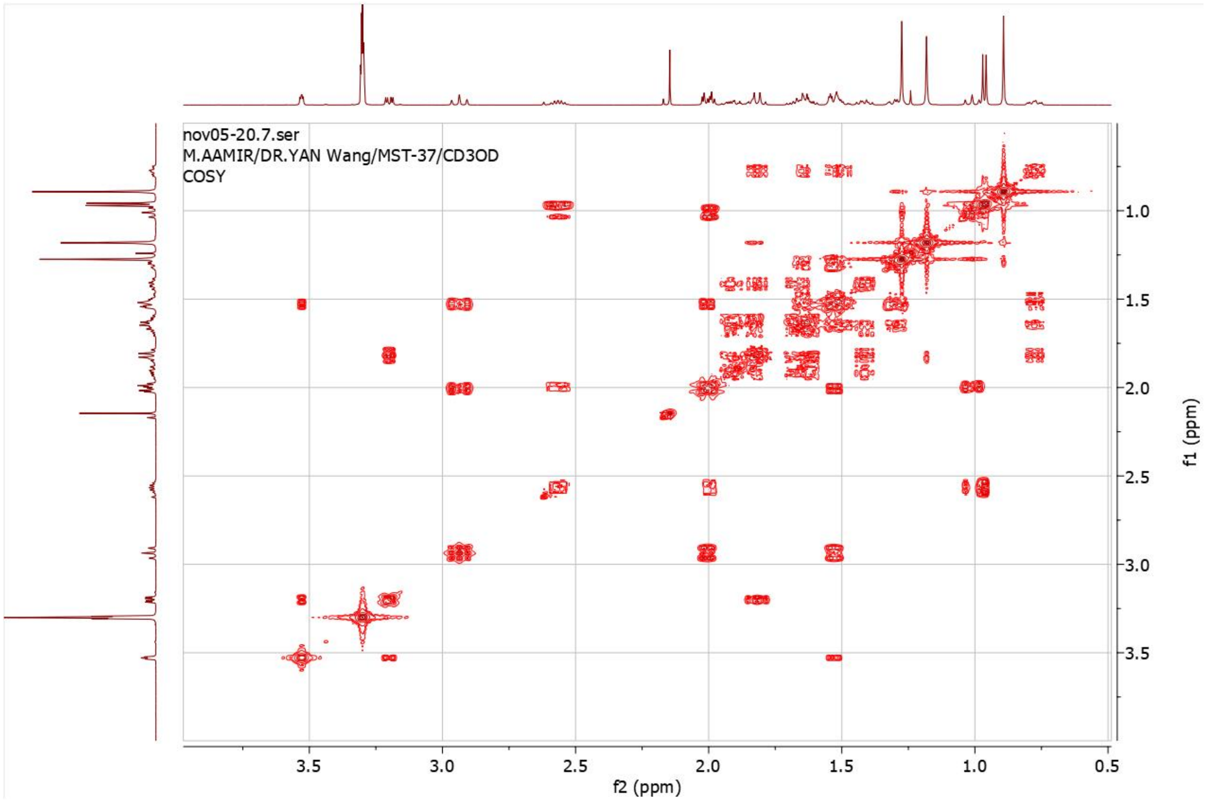

Figure S14. ^{1}H-^{1}H COSY spectrum of compound **2**

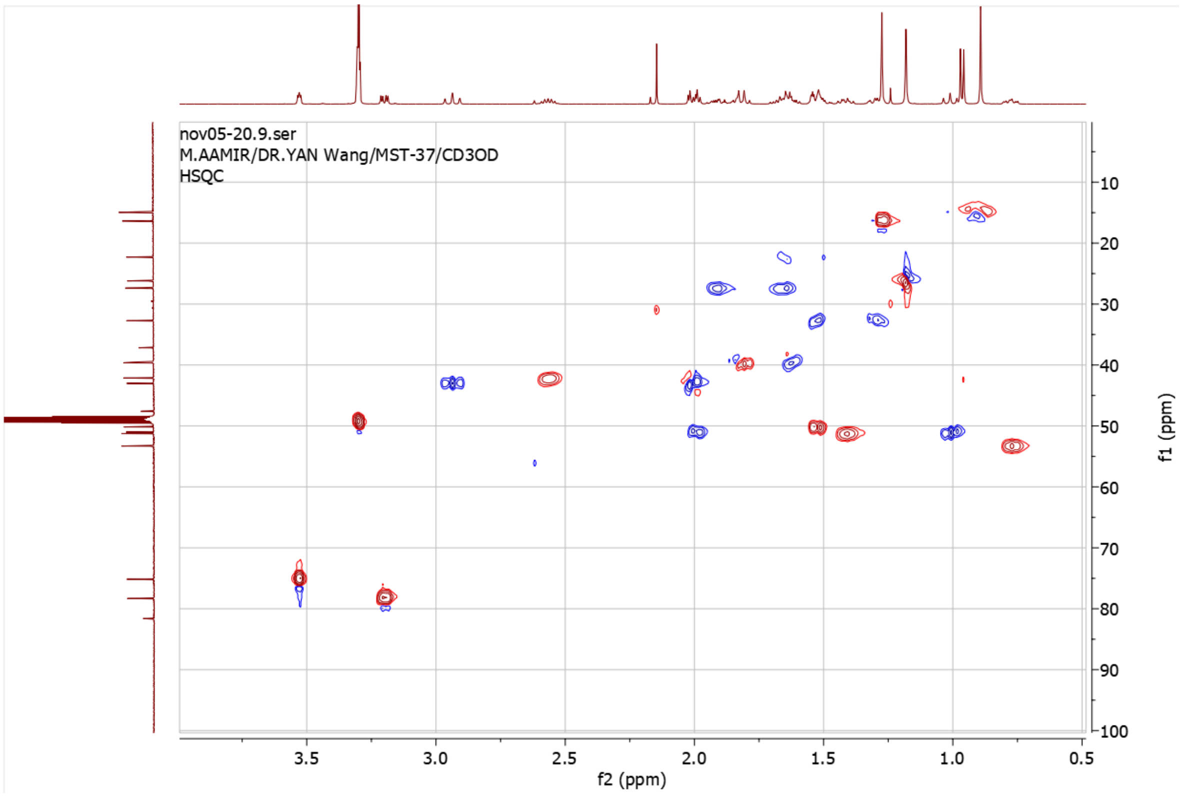

Figure S15. HSQC spectrum of compound **2**

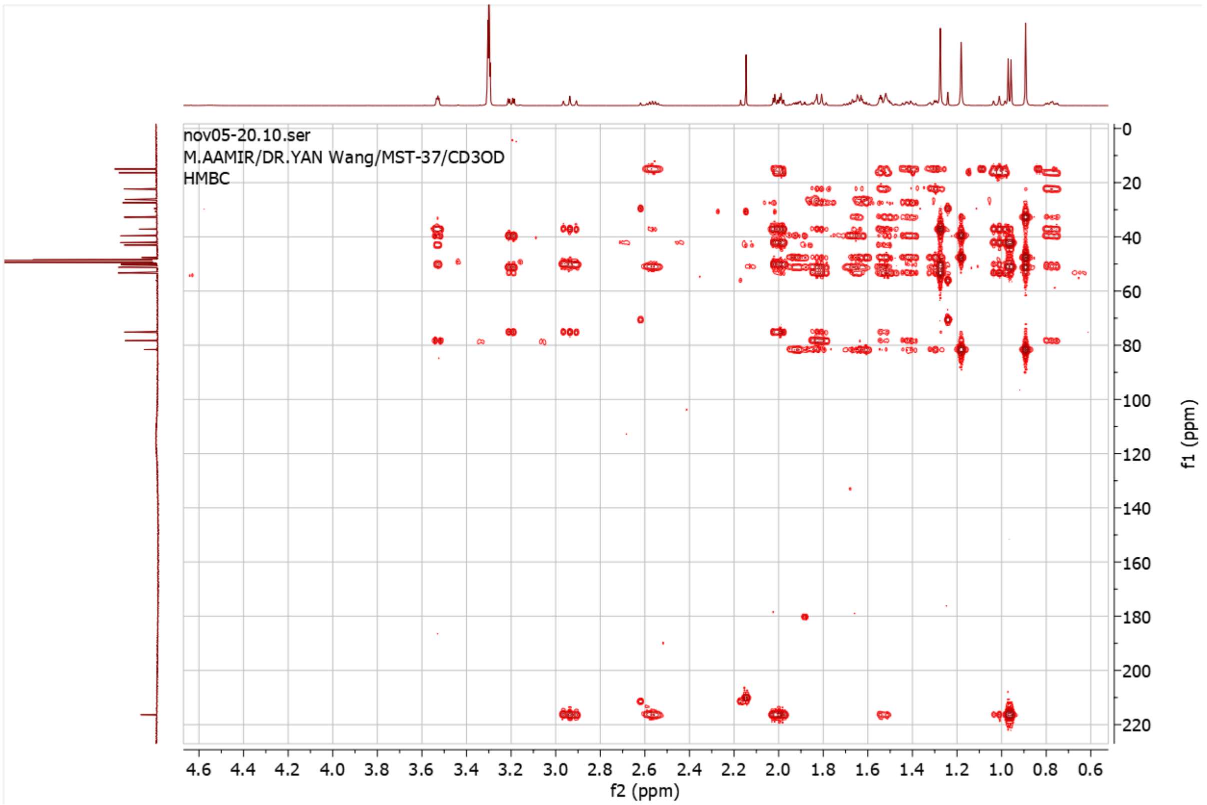

Figure S16. HMBC spectrum-1 of compound **2**

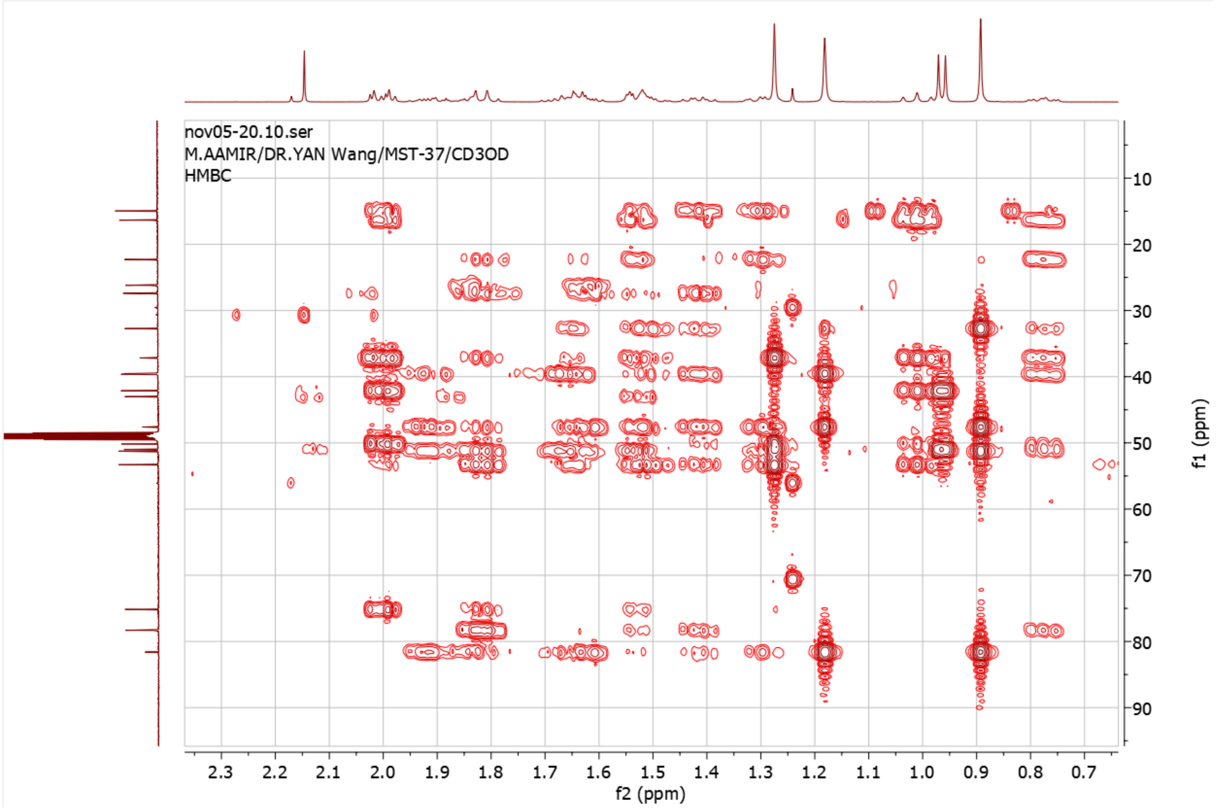

Figure S17. HMBC spectrum-2 of compound **2**

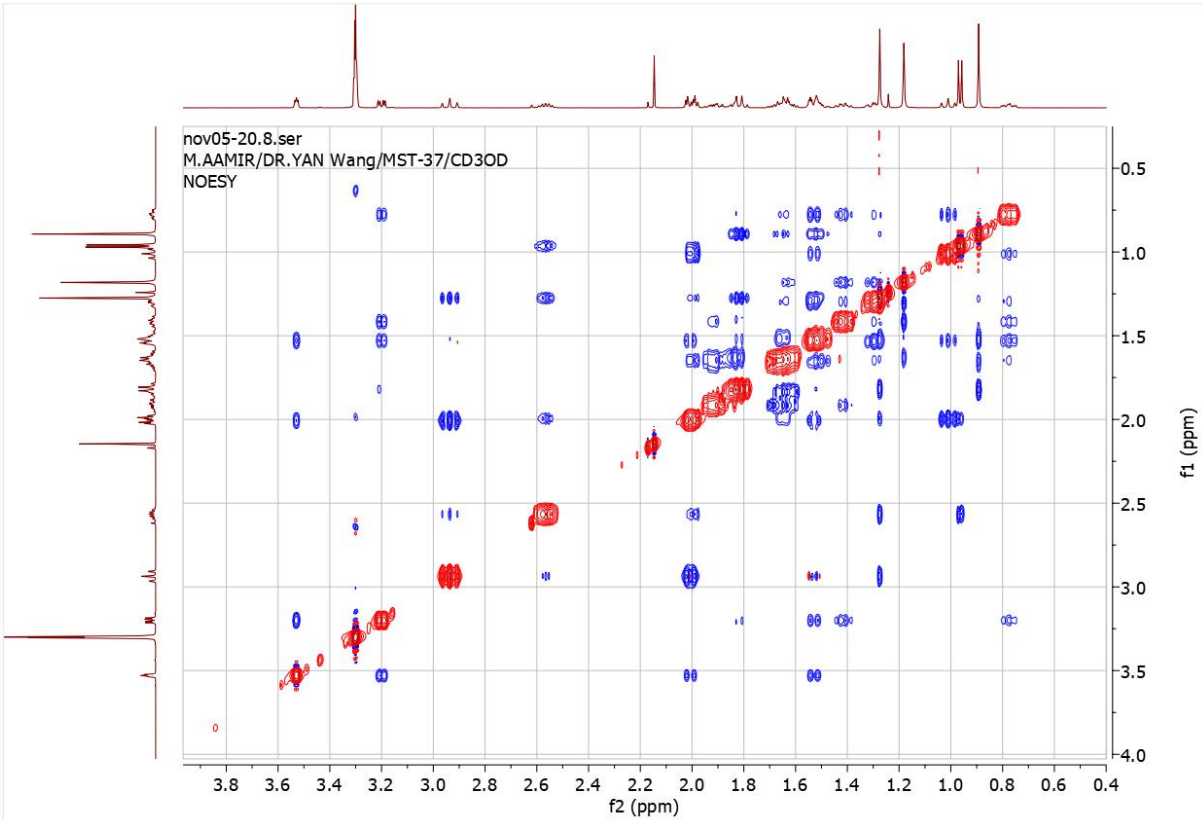

Figure S18. NOESY spectrum of compound **2**

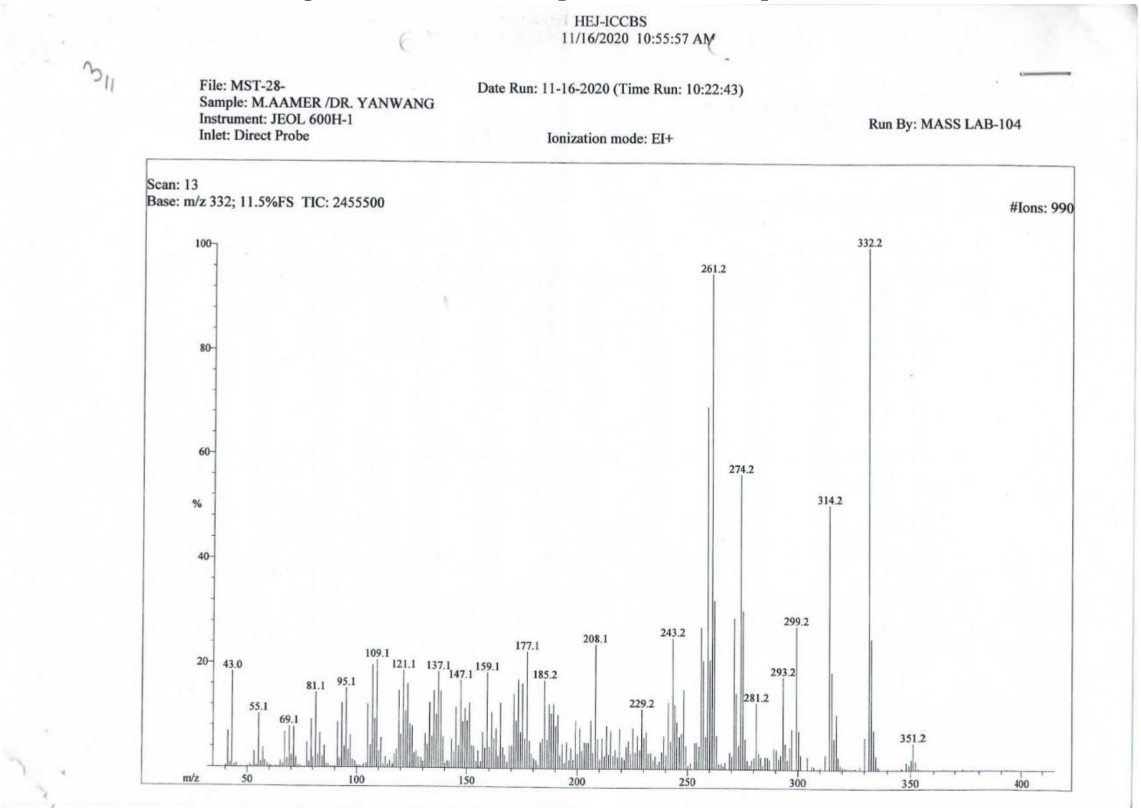

Figure S19. EI-MS spectrum of compound **3**

Mass	Relative Intensity	Theoretical Mass	Delta [ppm]	Delta [mmu]	RDB	Composition
		310.1780	37.6	11.7	5.0	$C_{17} H_{26} O_5$
311.1819	0.3	311.1800	6.1	1.9	13.5	$C_{24} H_{23}$
		311.1858	-12.8	-4.0	4.5	$C_{17} H_{27} O_5$
		311.1706	36.2	11.3	0.5	$C_{13} H_{27} O_8$
312.2176	5.2	312.2089	27.6	8.6	8.0	$C_{21} H_{28} O_2$
313.2085	3.3	313.2015	22.2	7.0	3.5	$C_{17} H_{29} O_5$
		313.2168	-26.5	-8.3	7.5	$C_{21} H_{29} O_2$
314.2246	72.9	314.2246	0.2	0.1	7.0	$C_{21} H_{30} O_2$
315.2306	24.2	315.2324	-5.6	-1.8	6.5	$C_{21} H_{31} O_2$
316.2345	6.8	316.2402	-18.0	-5.7	6.0	$C_{21} H_{32} O_2$
		316.2250	30.3	9.6	2.0	$C_{17} H_{32} O_5$
317.2109	51.1	317.2117	-2.4	-0.8	6.5	$C_{20} H_{29} O_3$
318.2160	12.0	318.2195	-11.0	-3.5	6.0	$C_{20} H_{30} O_3$
		318.2042	37.0	11.8	2.0	$C_{16} H_{30} O_6$
319.2259	1.8	319.2273	-4.6	-1.5	5.5	$C_{20} H_{31} O_3$
328.2148	0.8	328.2191	-13.2	-4.3	12.0	$C_{25} H_{28}$
		328.2250	-31.1	-10.2	3.0	$C_{18} H_{32} O_5$
		328.2038	33.3	10.9	8.0	$C_{21} H_{28} O_3$
330.2241	7.6	330.2195	14.0	4.6	7.0	$C_{21} H_{30} O_3$
		330.2348	-32.2	-10.6	11.0	$C_{25} H_{30}$
331.2283	3.2	331.2273	2.9	1.0	6.5	$C_{21} H_{31} O_3$
332.2345	61.6	332.2351	-1.9	-0.6	6.0	$C_{21} H_{32} O_3$
333.2370	15.7	333.2430	-17.8	-5.9	5.5	$C_{21} H_{33} O_3$
		333.2277	28.0	9.3	1.5	$C_{17} H_{33} O_6$
334.2527	4.0	334.2508	5.6	1.9	5.0	$C_{21} H_{34} O_3$
335.2445	1.0	335.2434	3.5	1.2	0.5	$C_{17} H_{35} O_6$
		335.2375	21.0	7.0	9.5	$C_{24} H_{31} O_1$
348.2103	1.0	348.2089	3.9	1.4	11.0	$C_{24} H_{28} O_2$
		348.2148	-13.0	-4.5	2.0	$C_{17} H_{32} O_7$
350.2439	0.5	350.2457	-5.2	-1.8	5.0	$C_{21} H_{34} O_4$
		350.2305	38.3	13.4	1.0	$C_{17} H_{34} O_7$
351.2530	0.3	351.2535	-1.5	-0.5	4.5	$C_{21} H_{35} O_4$

MSt-28

Figure S20. HREI-MS spectrum of compound **3**

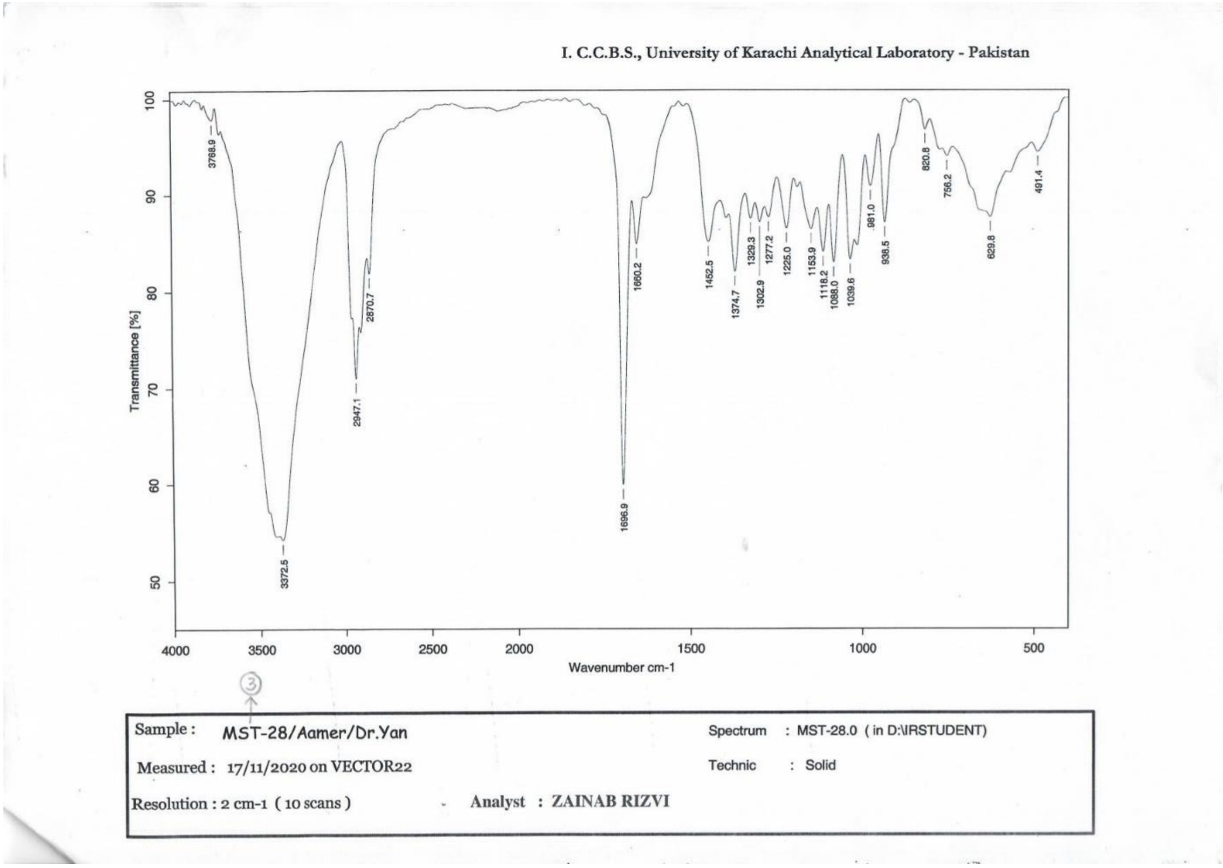

Figure S21. IR spectrum of compound **3**

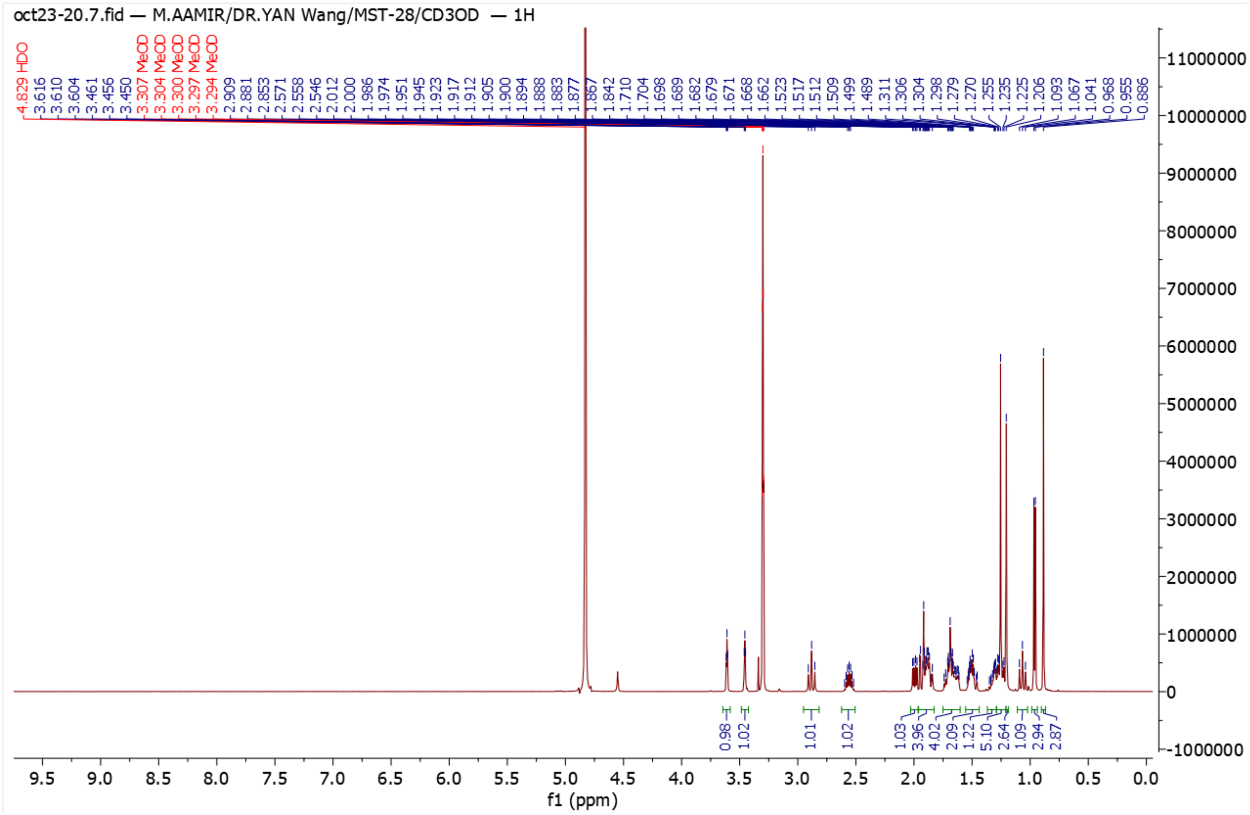

Figure S22. ^{1}H NMR spectrum-1 of compound **3** (500 MHz, CD_3OD)

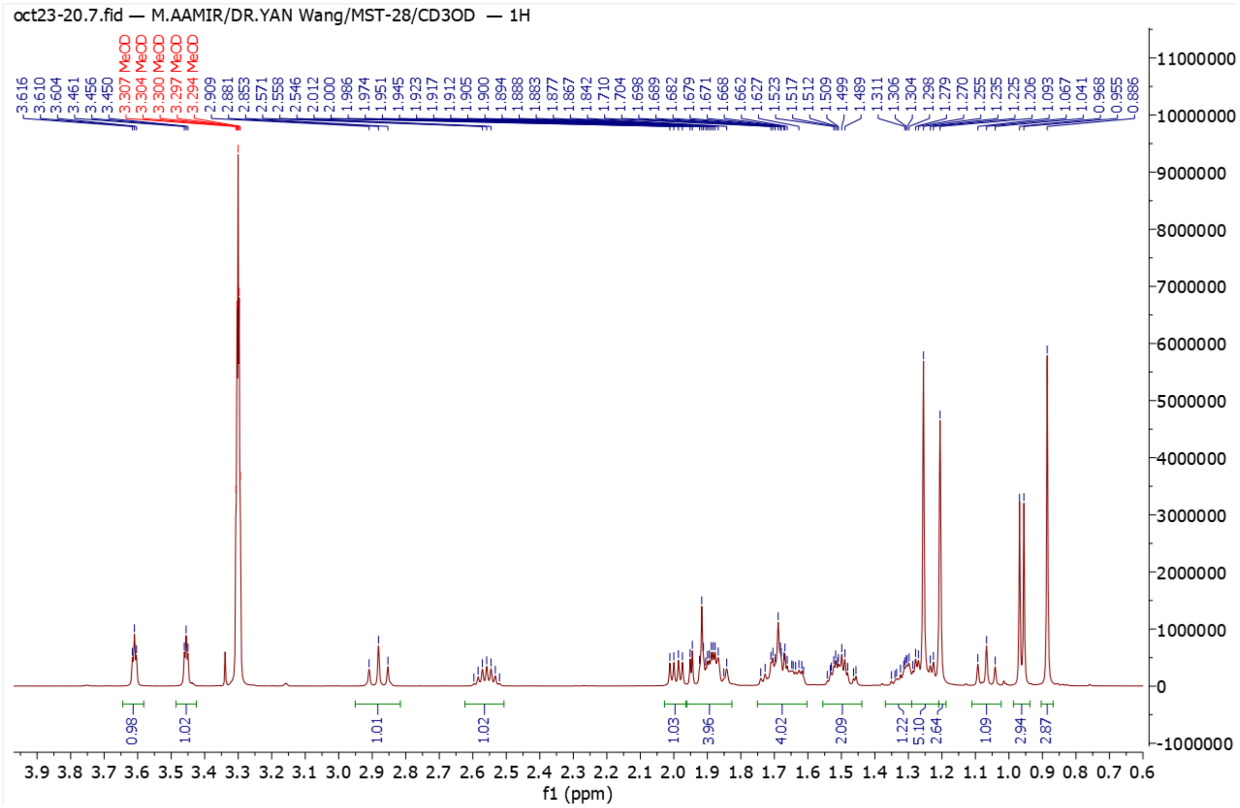

Figure S23. ^{1}H NMR spectrum-2 of compound **3** (500 MHz, CD_3OD)

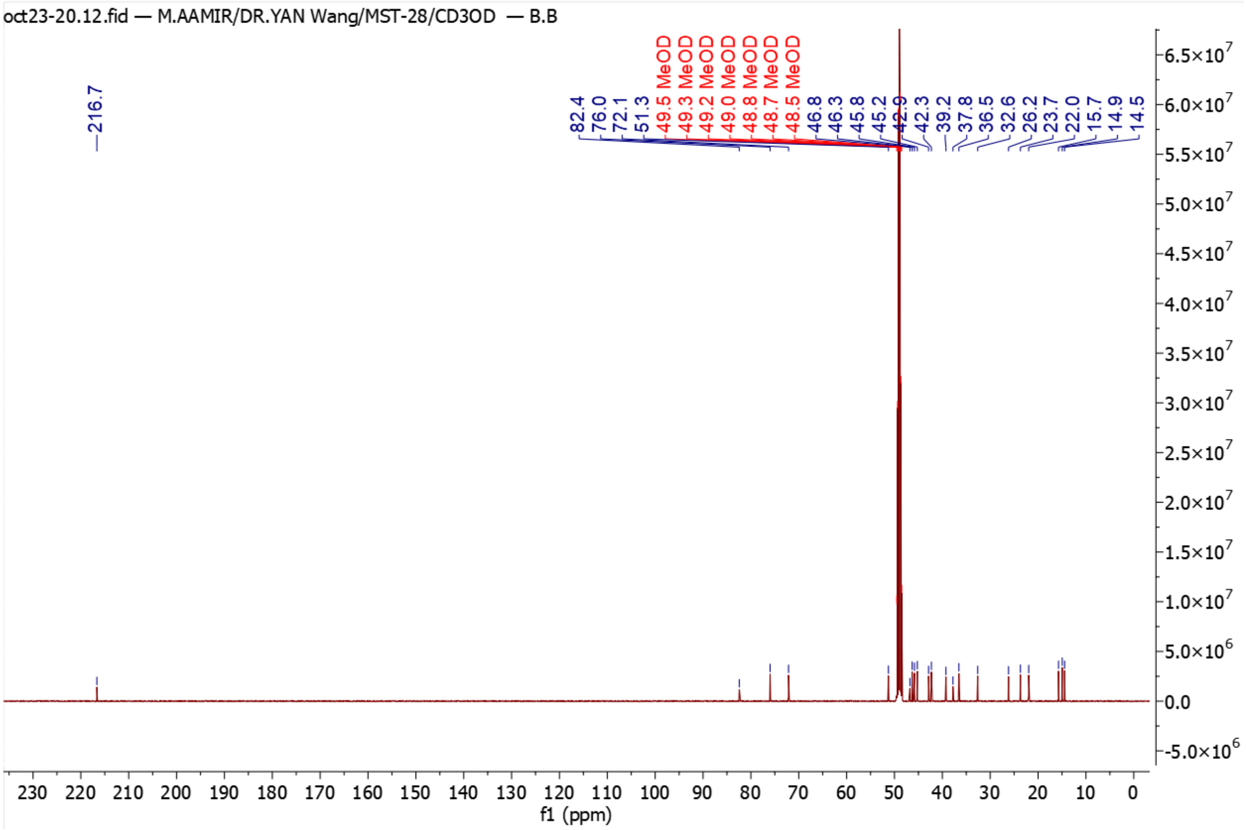

Figure S24. ^{13}C NMR spectrum-1 of compound **3** (125 MHz, CD_3OD)

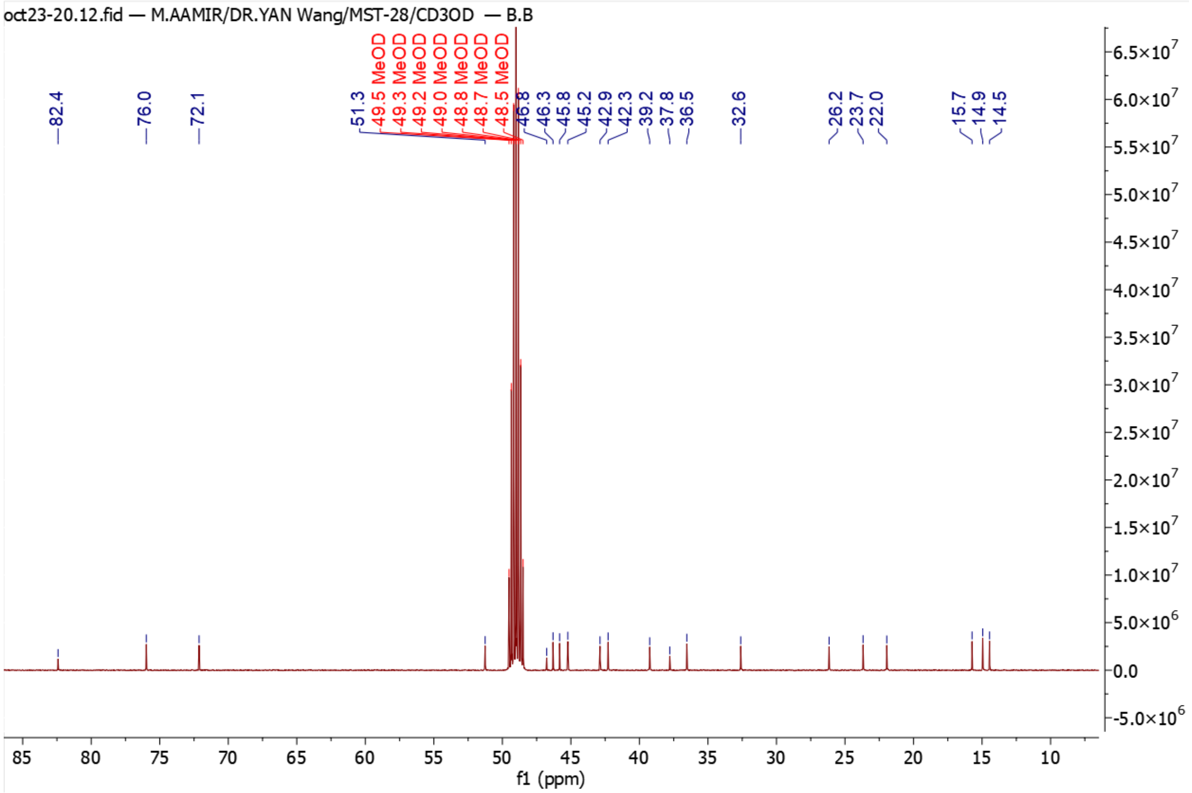

Figure S25. ^{13}C NMR spectrum-2 of compound **3** (125 MHz, CD_3OD)

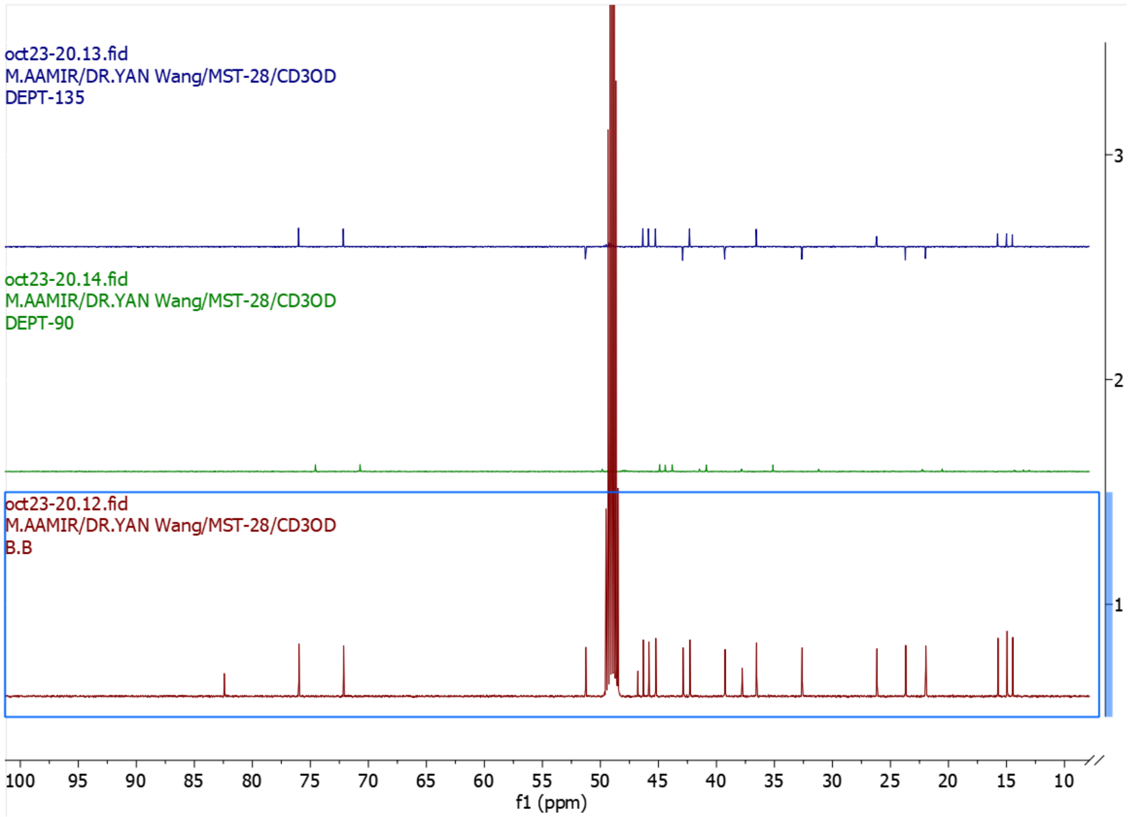

Figure S26. DEPT spectrum of compound **3**

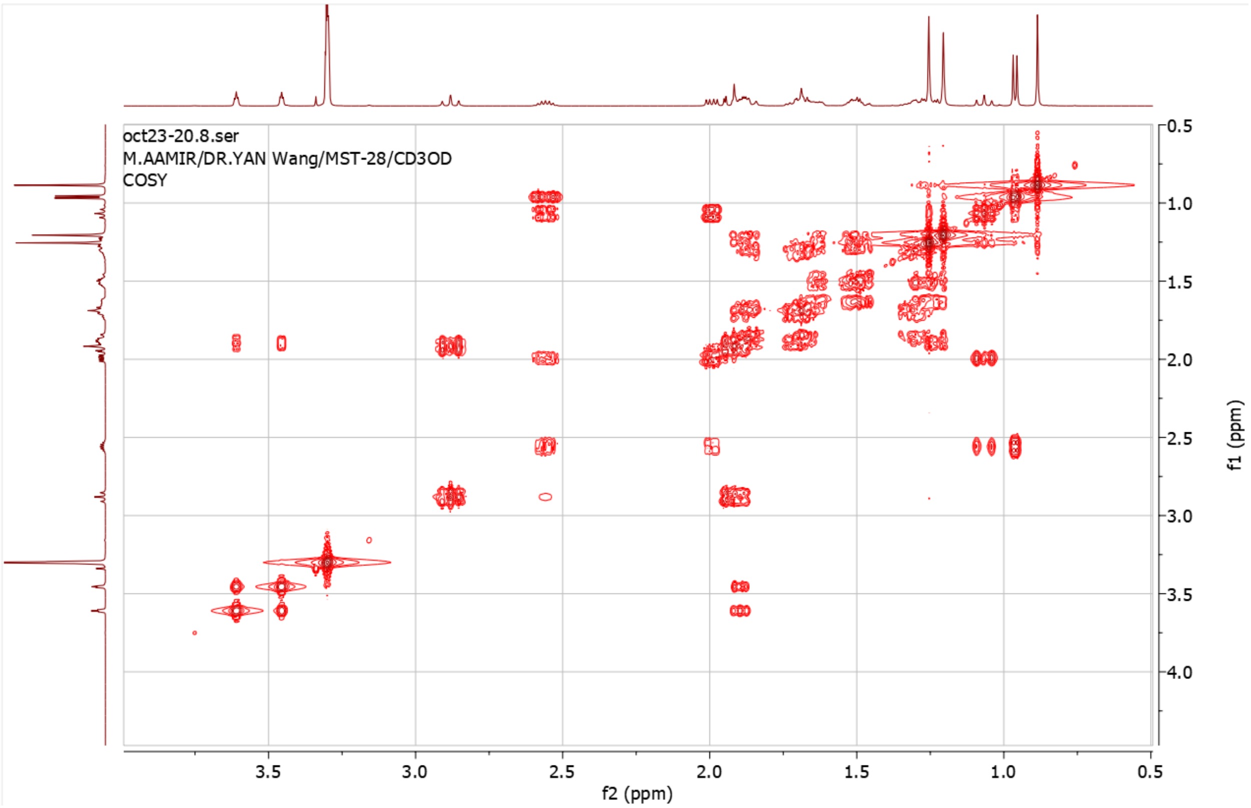

Figure S27. ^{1}H-^{1}H COSY spectrum of compound **3**

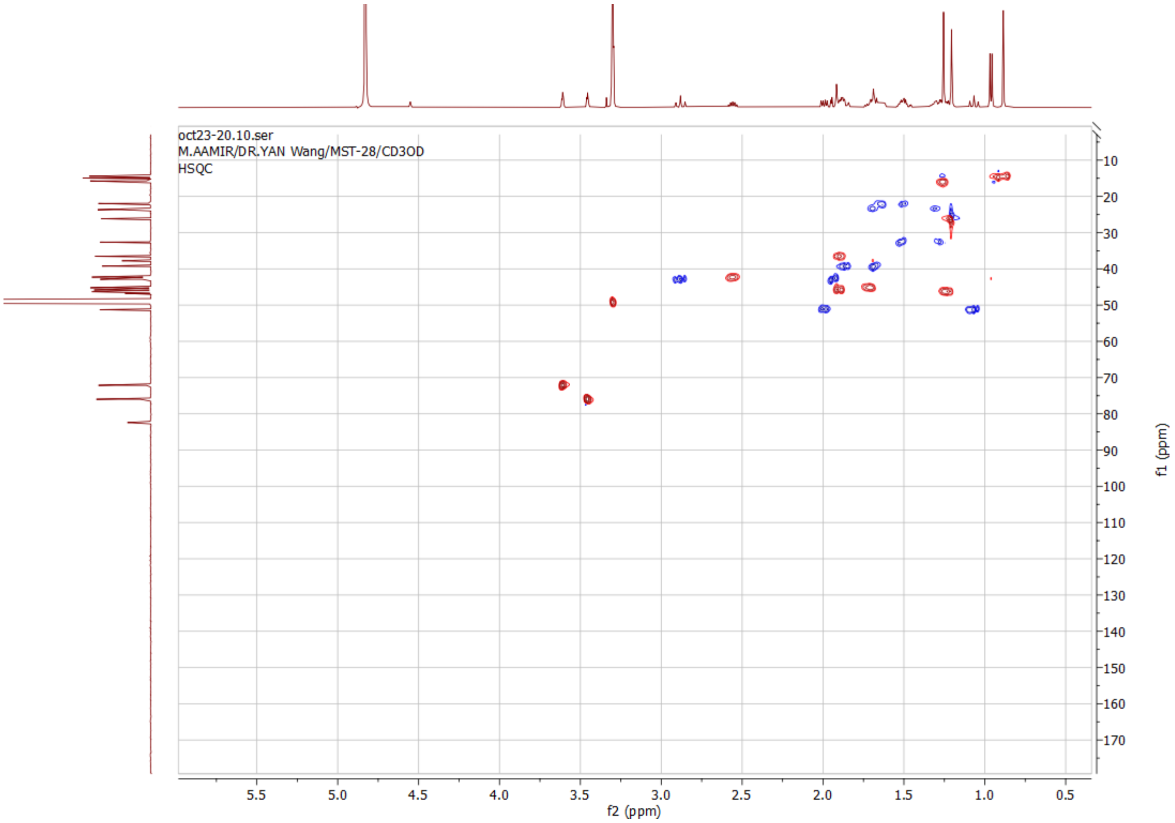

Figure S28. HSQC spectrum-1 of compound **3**

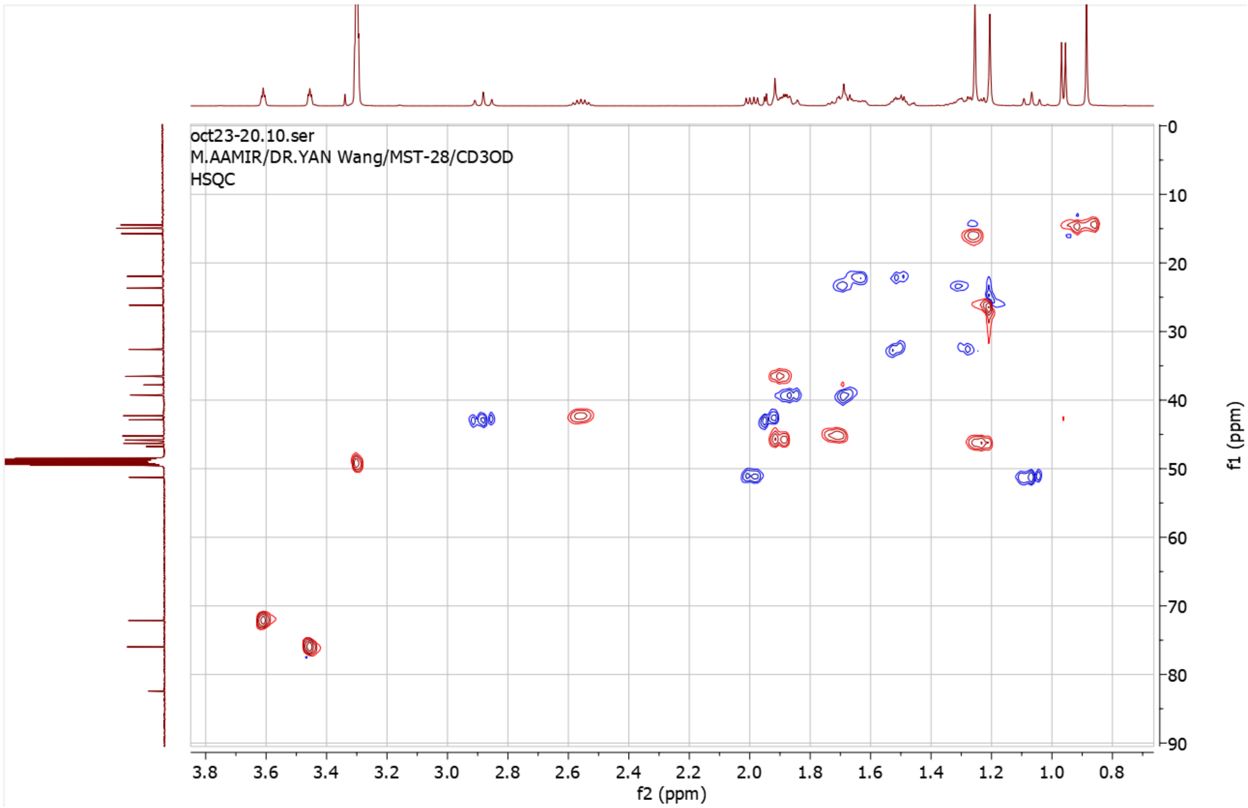

Figure S29. HSQC spectrum-2 of compound **3**

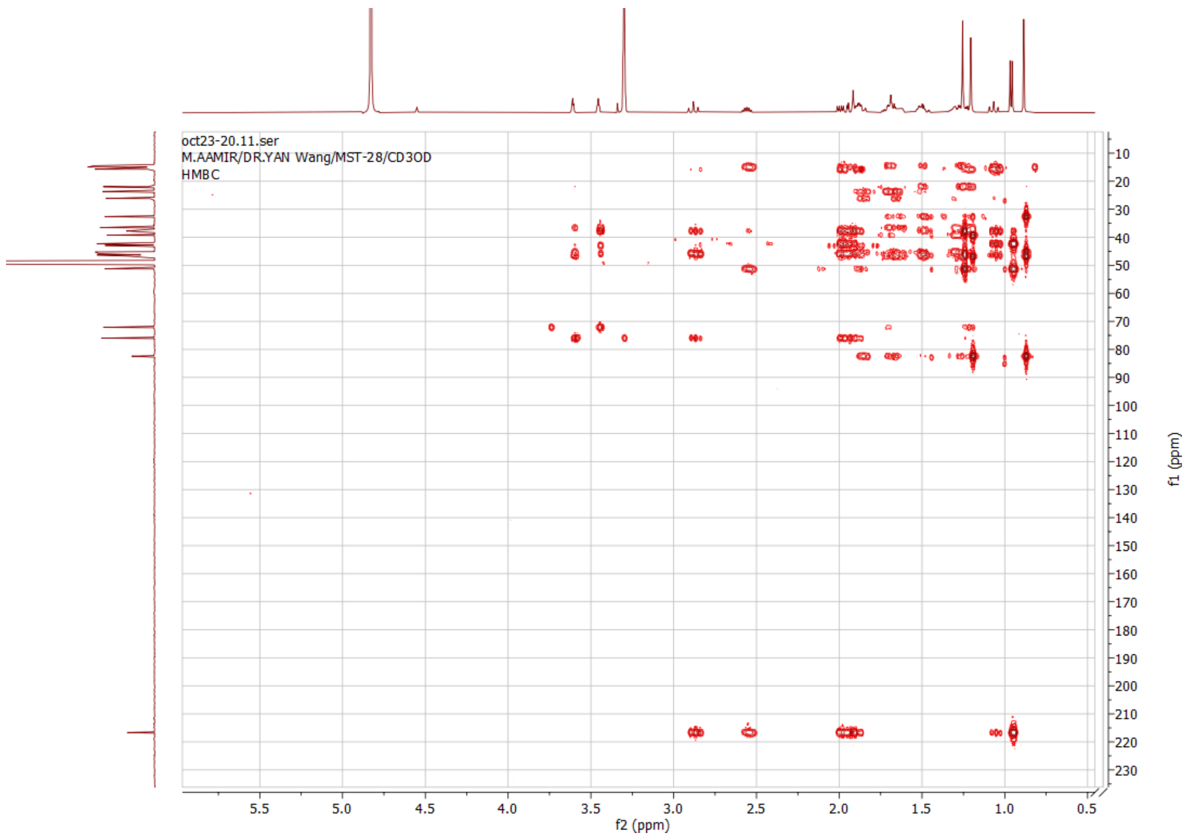

Figure S30. HMBC spectrum-1 of compound **3**

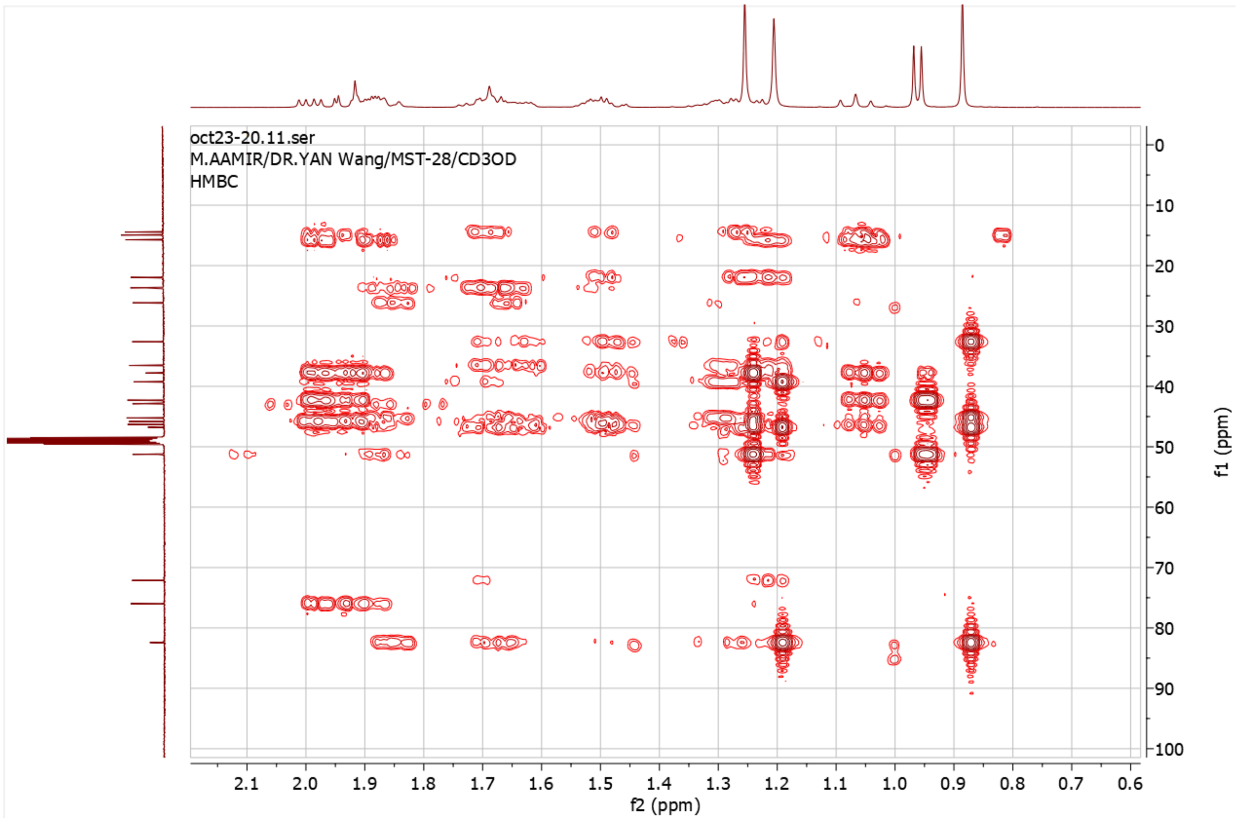

Figure S31. HMBC spectrum-2 of compound **3**

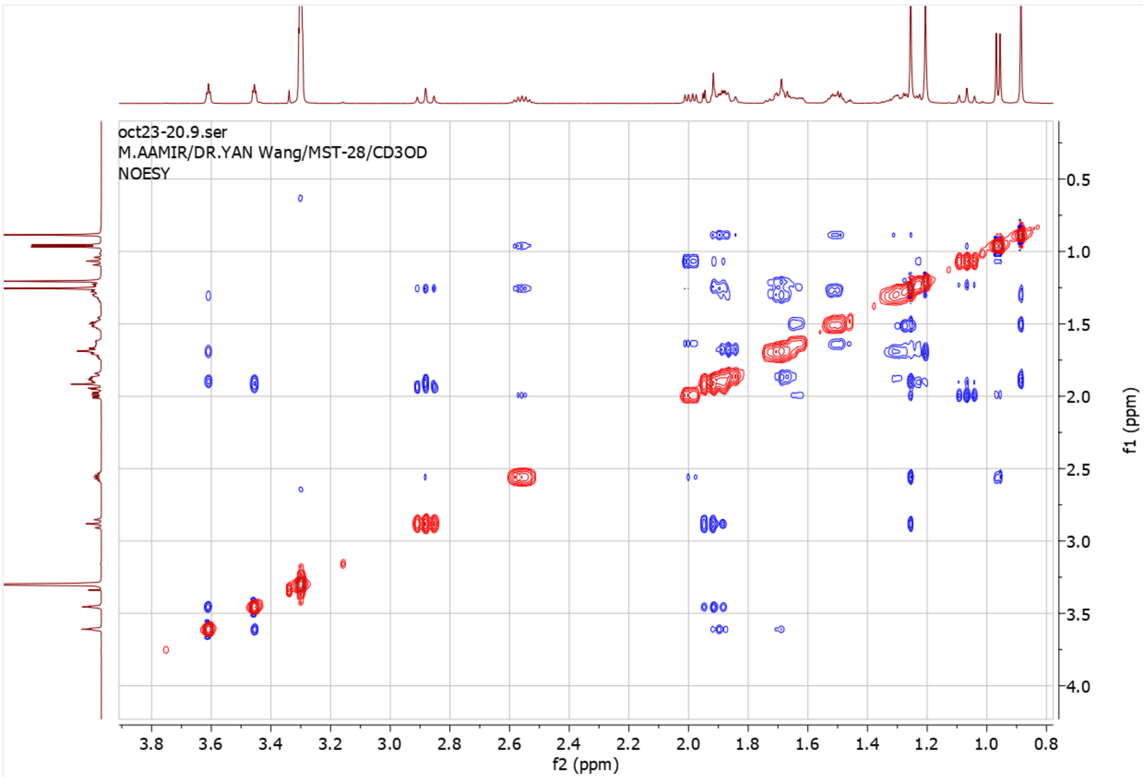

Figure S32. NOESY spectrum of compound **3**

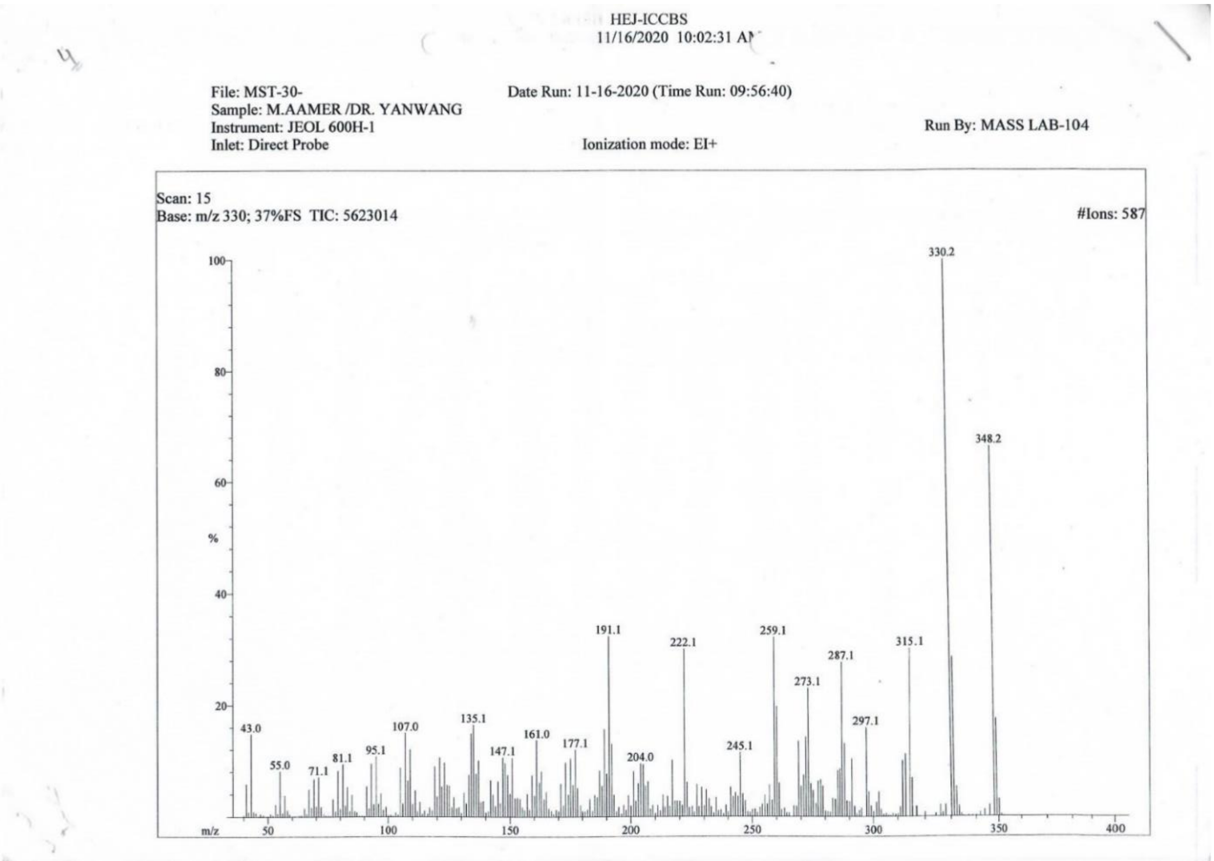

Figure S33. EI-MS spectrum of compound **4**

Mass	Relative Intensity	Theoretical Mass	Delta [ppm]	Delta [mmu]	RDB	Composition
287.2024	9.0	287.2011	4.4	1.3	6.5	$C_{19}H_{27}O_2$
288.1905	4.5	288.1878	9.5	2.7	11.0	$C_{22}H_{24}$
		288.1937	-10.9	-3.1	2.0	$C_{15}H_{28}O_5$
289.1816	1.2	289.1804	4.3	1.2	6.5	$C_{18}H_{25}O_3$
290.1845	1.0	290.1882	-12.7	-3.7	6.0	$C_{18}H_{26}O_3$
291.1963	3.3	291.1960	1.1	0.3	5.5	$C_{18}H_{27}O_3$
295.1946	1.1	295.1909	12.3	3.6	4.5	$C_{17}H_{27}O_4$
297.1851	10.7	297.1855	-1.4	-0.4	8.5	$C_{20}H_{25}O_2$
298.1926	2.6	298.1933	-2.2	-0.7	8.0	$C_{20}H_{26}O_2$
299.2004	2.2	299.2011	-2.3	-0.7	7.5	$C_{20}H_{27}O_2$
302.2079	1.3	302.2093	-4.7	-1.4	2.0	$C_{16}H_{30}O_5$
		302.2035	14.7	4.5	11.0	$C_{23}H_{26}$
312.2023	5.9	312.2089	-21.2	-6.6	8.0	$C_{21}H_{28}O_2$
		312.1937	27.6	8.6	4.0	$C_{17}H_{28}O_5$
313.2092	4.0	313.2168	-24.1	-7.5	7.5	$C_{21}H_{29}O_2$
		313.2015	24.6	7.7	3.5	$C_{17}H_{29}O_5$
314.2201	1.7	314.2246	-14.3	-4.5	7.0	$C_{21}H_{30}O_2$
		314.2093	34.3	10.8	3.0	$C_{17}H_{30}O_5$
315.1947	18.0	315.1960	-4.2	-1.3	7.5	$C_{20}H_{27}O_3$
316.2022	3.9	316.2038	-5.3	-1.7	7.0	$C_{20}H_{26}O_3$
328.2081	1.0	328.2038	12.9	4.2	8.0	$C_{21}H_{28}O_3$
		328.2191	-33.6	-11.0	12.0	$C_{25}H_{28}$
330.2218	25.4	330.2195	7.1	2.3	7.0	$C_{21}H_{30}O_3$
331.2249	6.9	331.2273	-7.4	-2.5	6.5	$C_{21}H_{31}O_3$
332.2369	2.4	332.2351	5.4	1.8	6.0	$C_{21}H_{32}O_3$
348.2305	11.4	348.2301	1.3	0.5	6.0	$C_{21}H_{32}O_4$
349.2318	2.6	349.2379	-17.4	-6.1	5.5	$C_{21}H_{33}O_4$
		349.2226	26.3	9.2	1.5	$C_{17}H_{33}O_7$

Activate Windows
Go to Settings to acti

MSt-30

Figure S34. HREI-MS spectrum of compound **4**

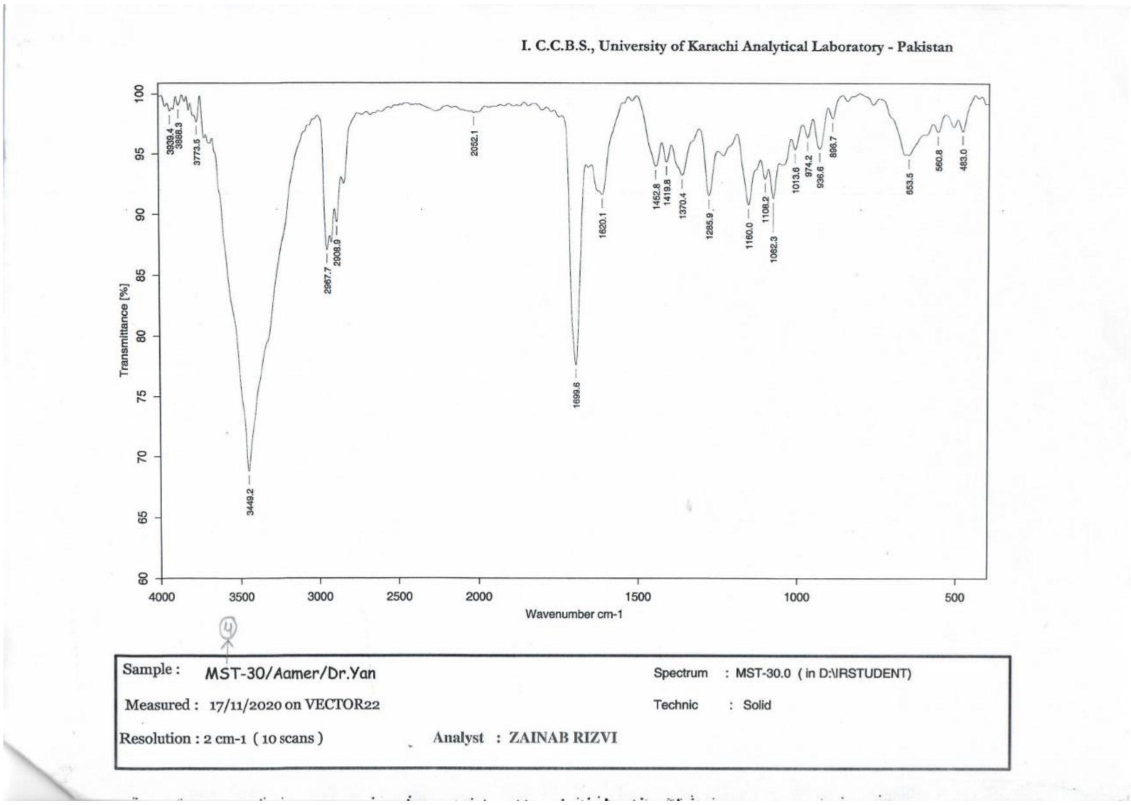

Figure S35. IR spectrum of compound **4**

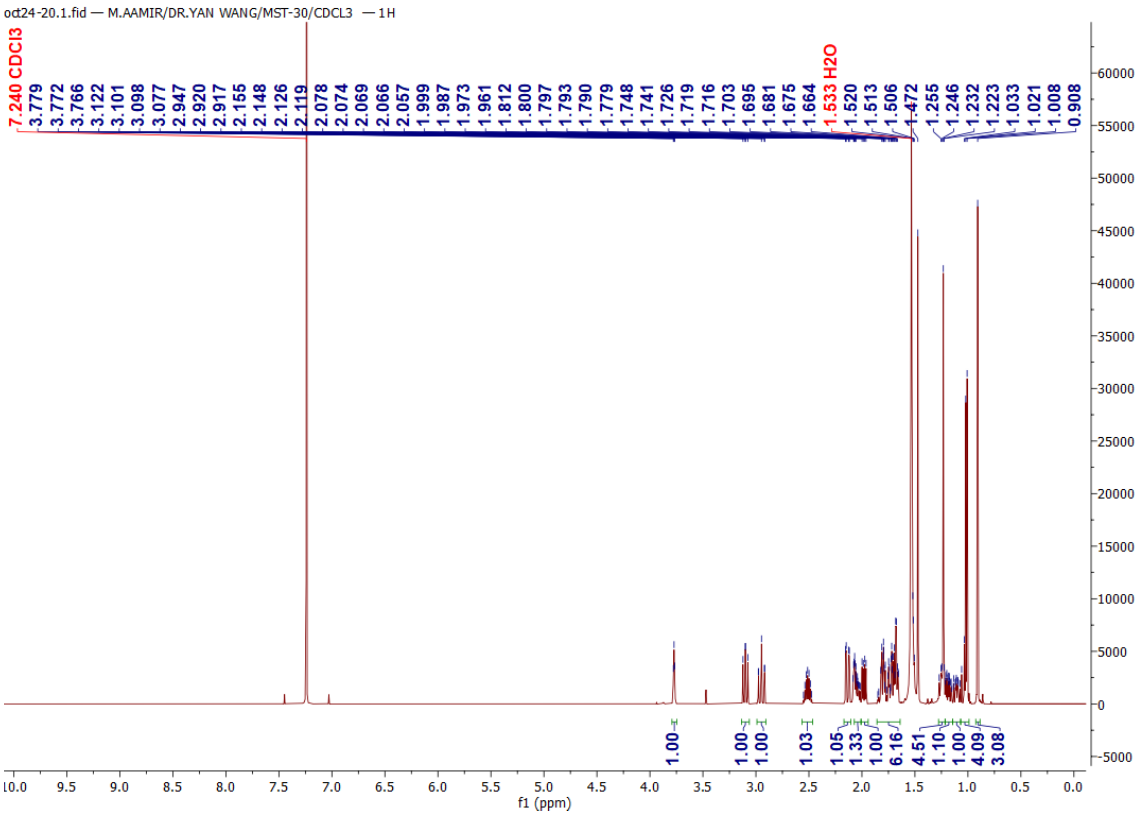

Figure S36. ^{1}H NMR spectrum-1 of compound **4** (500 MHz, CD_3OD)

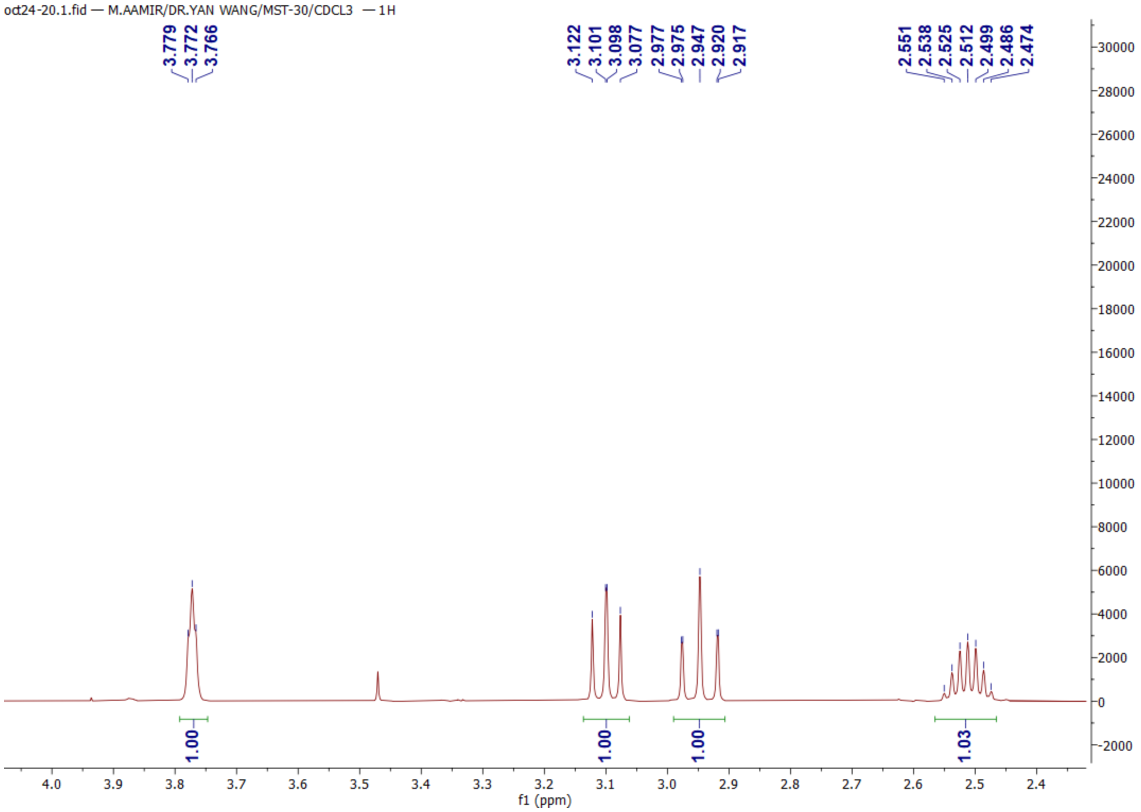

Figure S37. ^{1}H NMR spectrum-2 of compound **4** (500 MHz, CD_3OD)

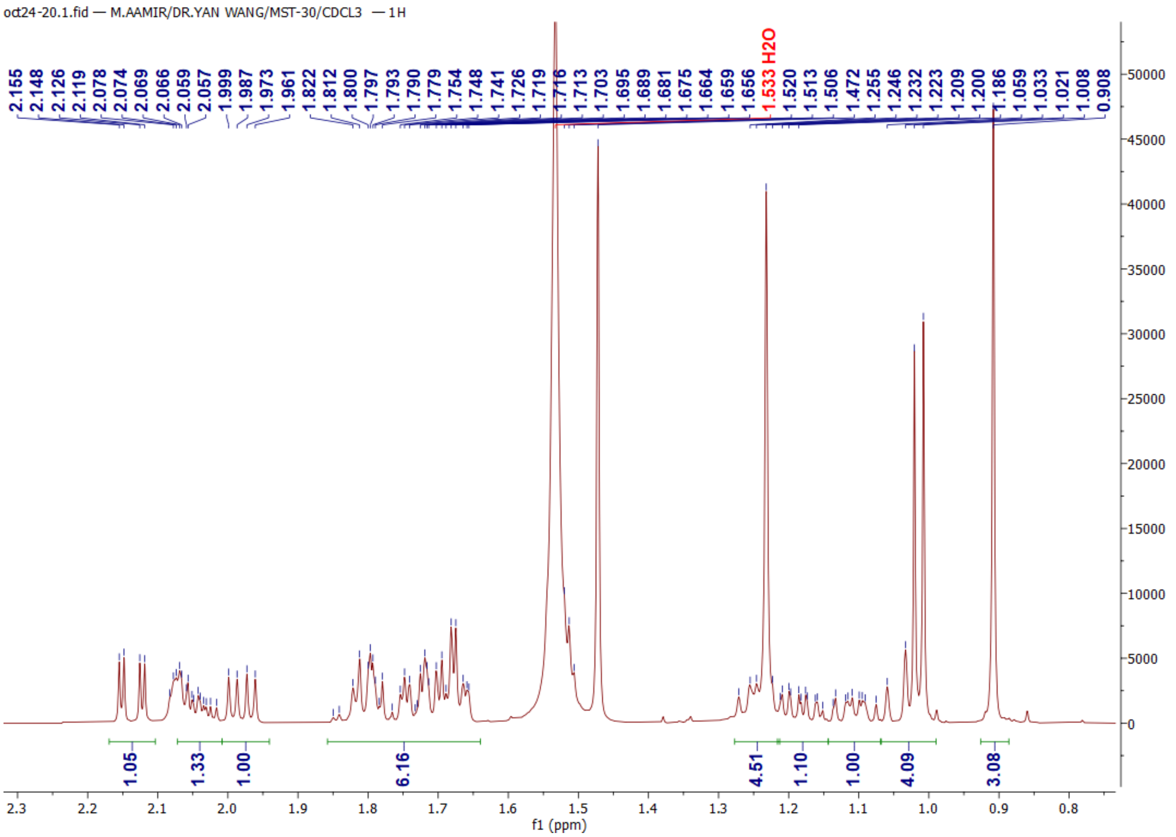

Figure S38. ^{1}H NMR spectrum-3 of compound **4** (500 MHz, CD_3OD)

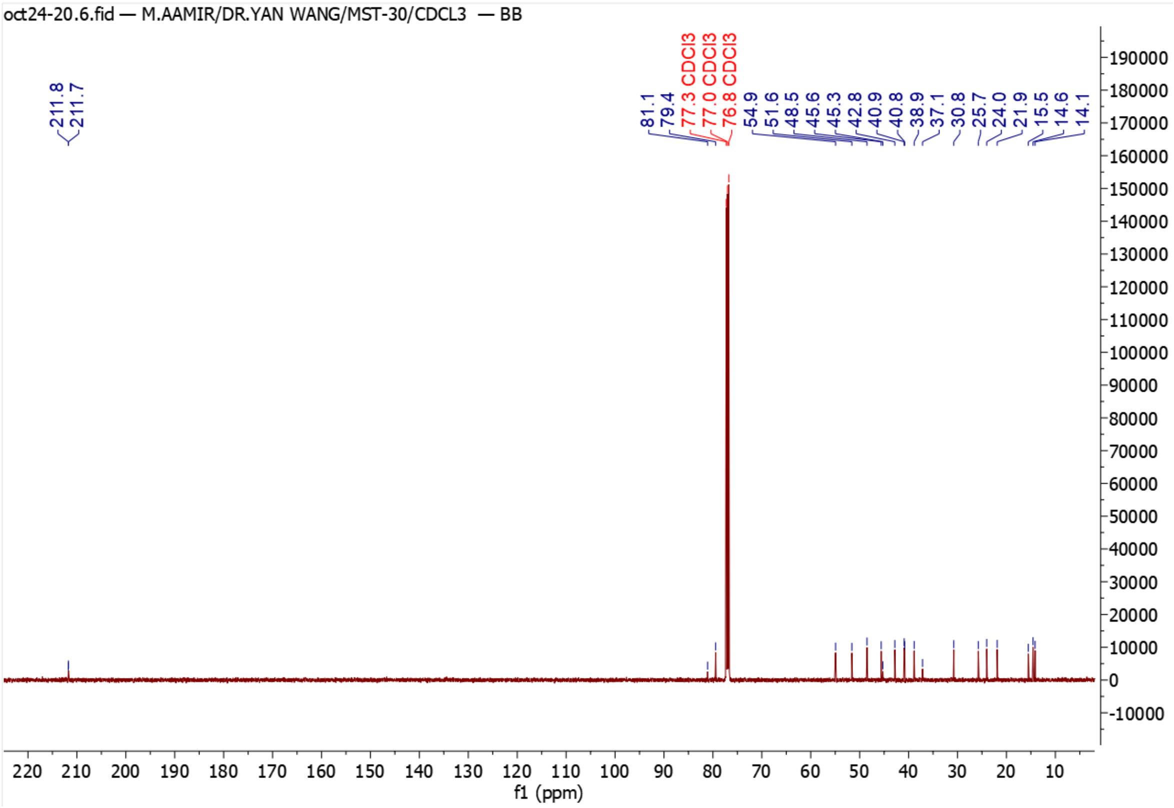

Figure S39. ^{13}C NMR spectrum-1 of compound **4** (125 MHz, CD_3OD)

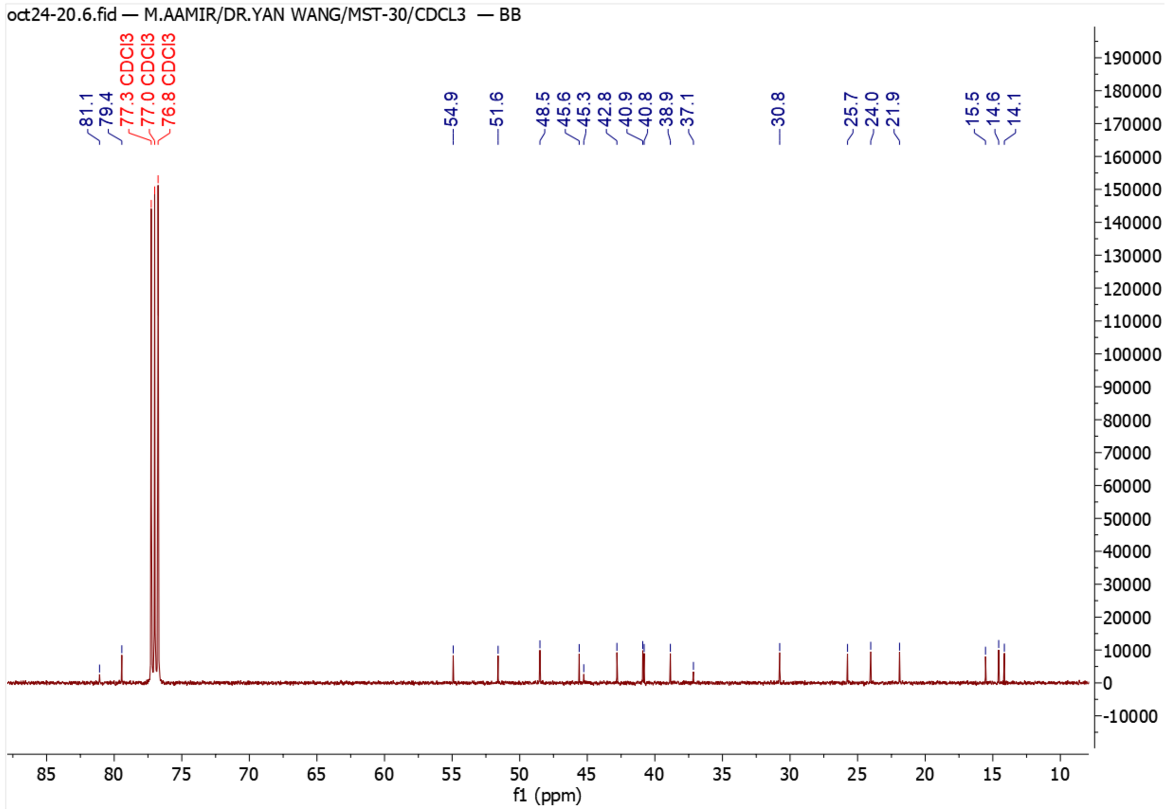

Figure S40. ^{13}C NMR spectrum-2 of compound **4** (125 MHz, CD_3OD)

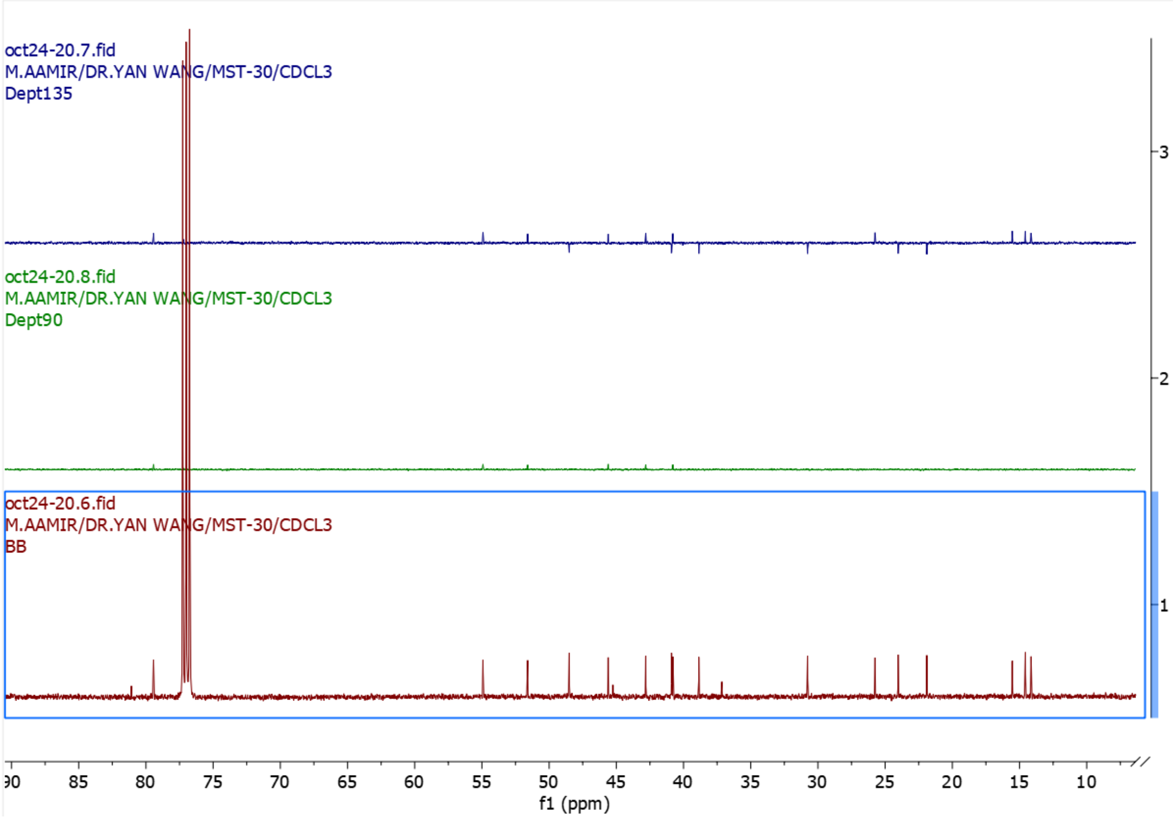

Figure S41. DEPT spectrum of compound **4**

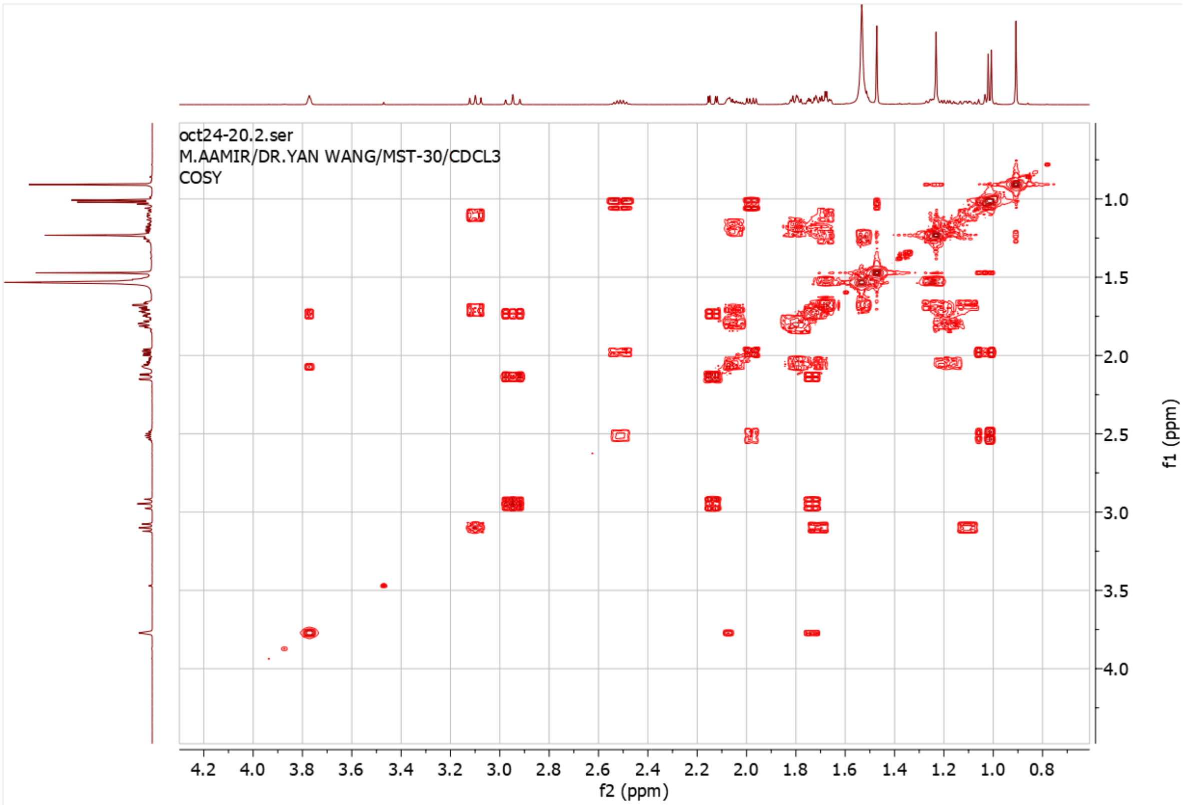

Figure S42. ^{1}H-^{1}H COSY spectrum of compound **4**

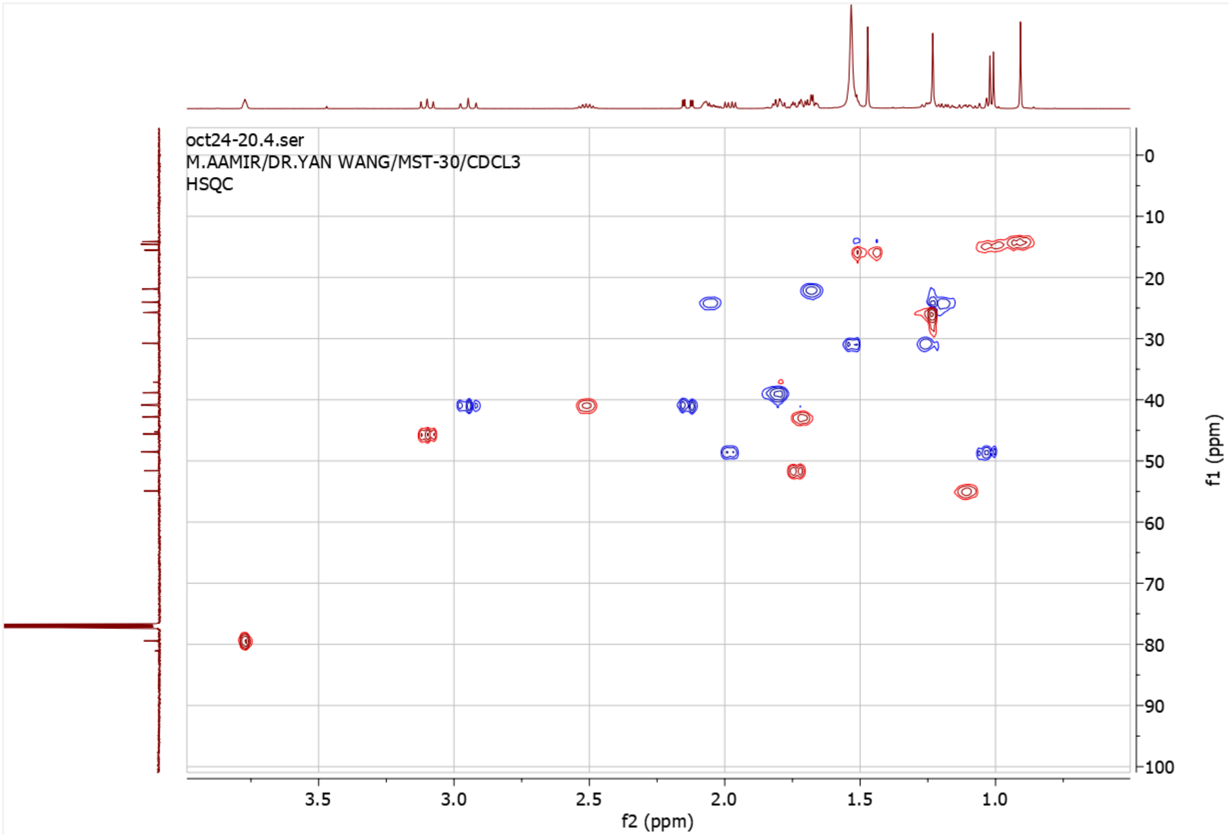

Figure S43. HSQC spectrum of compound **4**

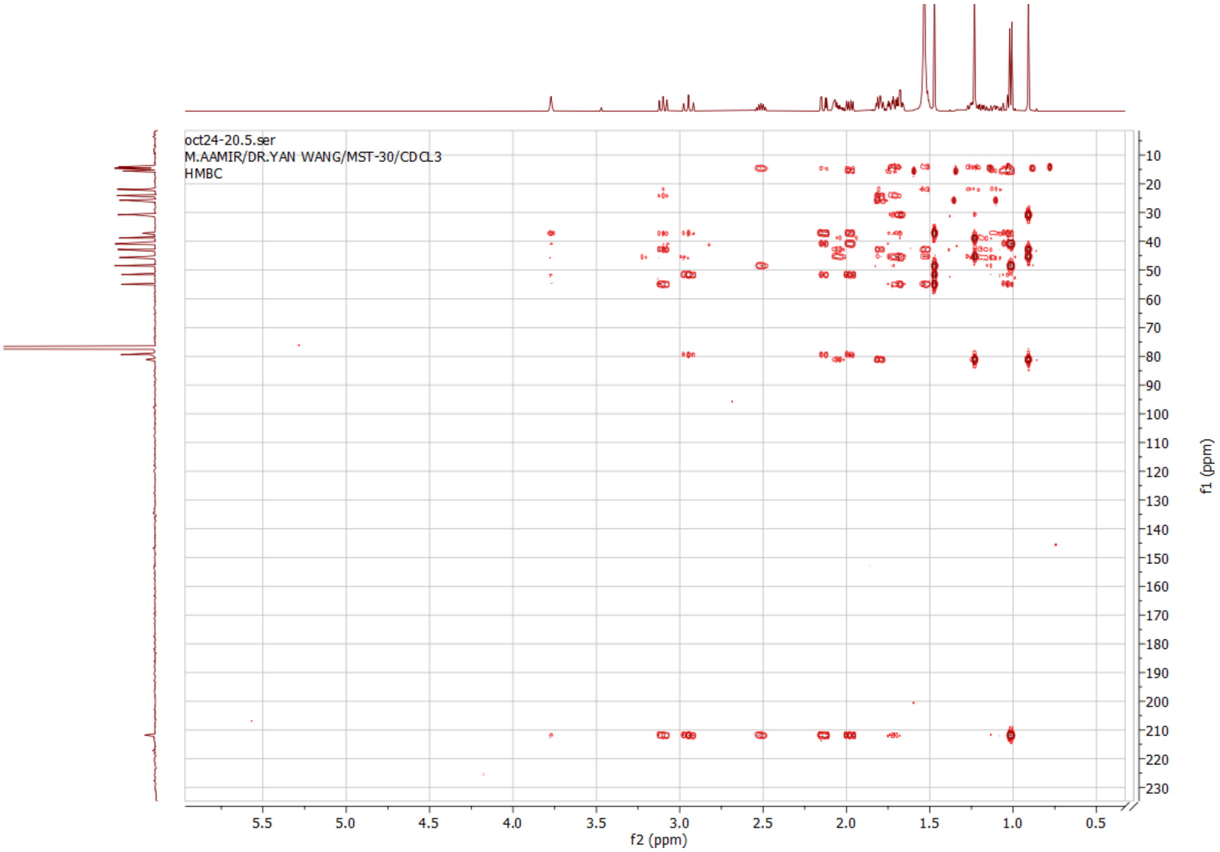

Figure S44. HMBC spectrum-1 of compound **4**

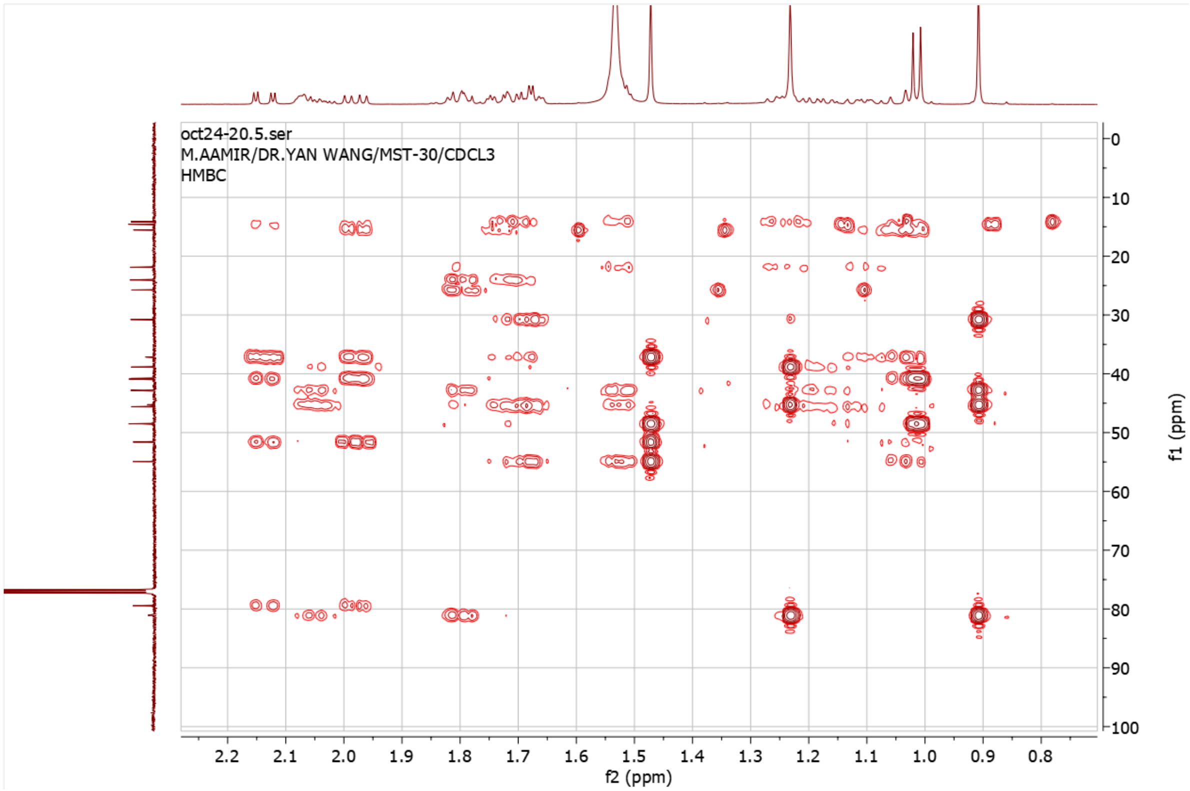

Figure S45. HMBC spectrum-2 of compound **4**

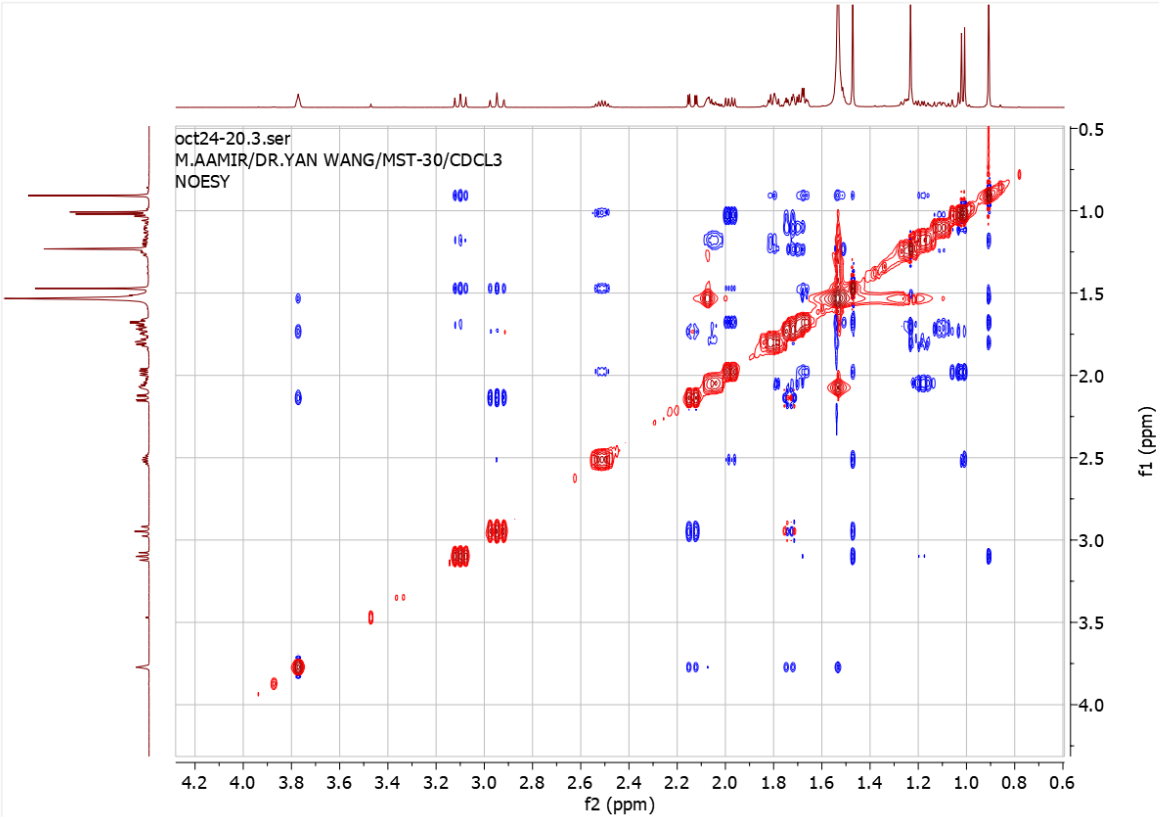

Figure S46. NOESY spectrum of compound **4**

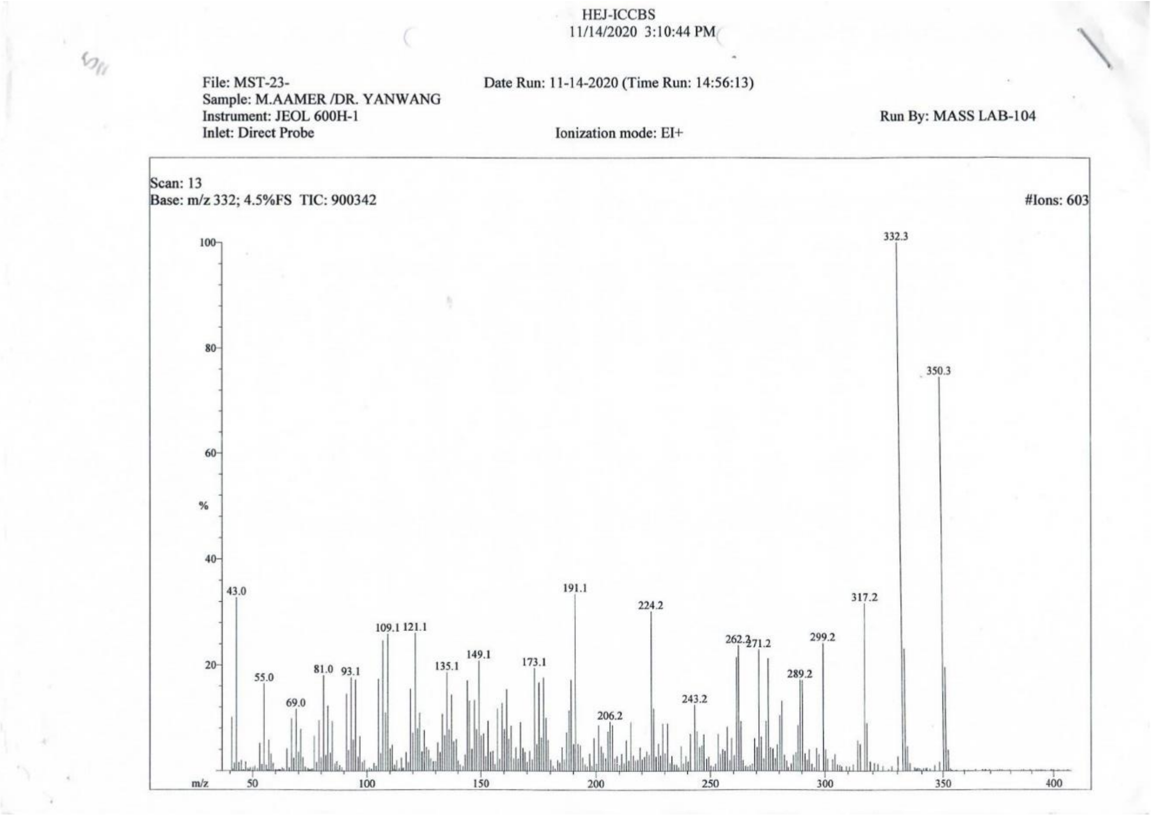

Figure S47. EI-MS spectrum of compound **5**

Mass	Relative Intensity	Theoretical Mass	Delta [ppm]	Delta [mmu]	RDB	Composition
		304.2402	-33.4	-10.2	5.0	$C_{20} H_{32} O_2$
312.2042	2.5	312.2089	-15.1	-4.7	8.0	$C_{21} H_{28} O_2$
		312.1937	33.7	10.5	4.0	$C_{17} H_{28} O_5$
313.2201	1.6	313.2168	10.8	3.4	7.5	$C_{21} H_{29} O_2$
314.2228	33.1	314.2246	-5.8	-1.8	7.0	$C_{21} H_{30} O_2$
315.2235	12.2	315.2171	20.1	6.3	2.5	$C_{17} H_{31} O_5$
		315.2324	-28.3	-8.9	6.5	$C_{21} H_{31} O_2$
316.2282	4.4	316.2250	10.1	3.2	2.0	$C_{17} H_{32} O_5$
		316.2191	28.7	9.1	11.0	$C_{24} H_{28}$
317.2169	40.6	317.2117	16.3	5.2	6.5	$C_{20} H_{29} O_3$
		317.2269	-31.8	-10.1	10.5	$C_{24} H_{29}$
318.2145	8.5	318.2195	-15.7	-5.0	6.0	$C_{20} H_{30} O_3$
		318.2042	32.2	10.3	2.0	$C_{16} H_{30} O_6$
319.2194	2.6	319.2121	22.8	7.3	1.5	$C_{16} H_{31} O_6$
		319.2273	-25.0	-8.0	5.5	$C_{20} H_{31} O_3$
330.2229	4.8	330.2195	10.3	3.4	7.0	$C_{21} H_{30} O_3$
331.2242	3.0	331.2273	-9.3	-3.1	6.5	$C_{21} H_{31} O_3$
332.2384	100.0	332.2351	9.6	3.2	6.0	$C_{21} H_{32} O_3$
333.2411	23.5	333.2430	-5.7	-1.9	5.5	$C_{21} H_{33} O_3$
334.2448	4.7	334.2508	-18.0	-6.0	5.0	$C_{21} H_{34} O_3$
		334.2355	27.7	9.2	1.0	$C_{17} H_{34} O_6$
335.2363	1.5	335.2375	-3.4	-1.2	9.5	$C_{24} H_{31} O_1$
		335.2434	-21.0	-7.0	0.5	$C_{17} H_{35} O_6$
348.2311	1.8	348.2301	3.1	1.1	6.0	$C_{21} H_{32} O_4$
350.2454	38.2	350.2457	-0.9	-0.3	5.0	$C_{21} H_{34} O_4$
351.2453	8.9	351.2383	20.1	7.1	0.5	$C_{17} H_{35} O_7$
		351.2535	-23.3	-8.2	4.5	$C_{21} H_{35} O_4$
352.2461	1.5	352.2461	0.0	0.0	0.0	$C_{17} H_{36} O_7$
		352.2402	16.7	5.9	9.0	$C_{24} H_{32} O_2$

Activate Wind
Go to Settings to a

Mst-23

Figure S48. HREI-MS spectrum of compound **5**

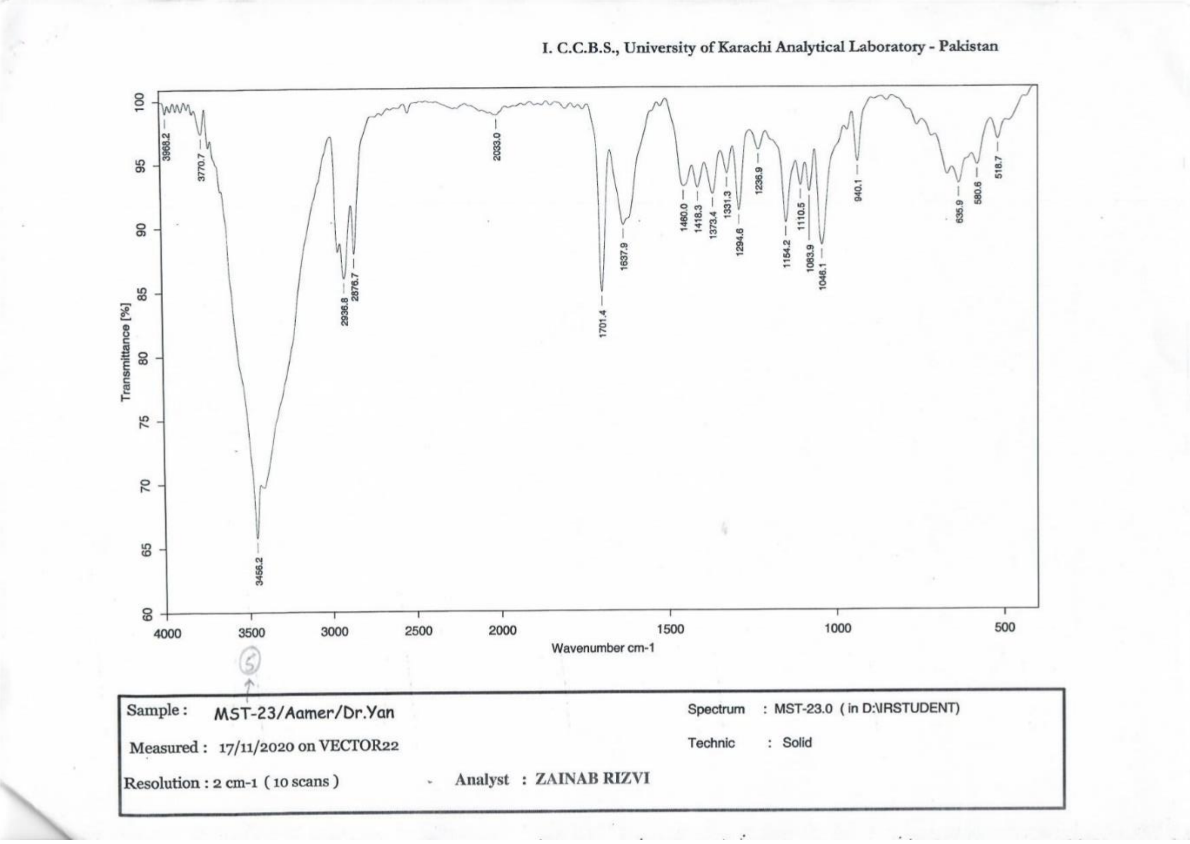

Figure S49. IR spectrum of compound **5**

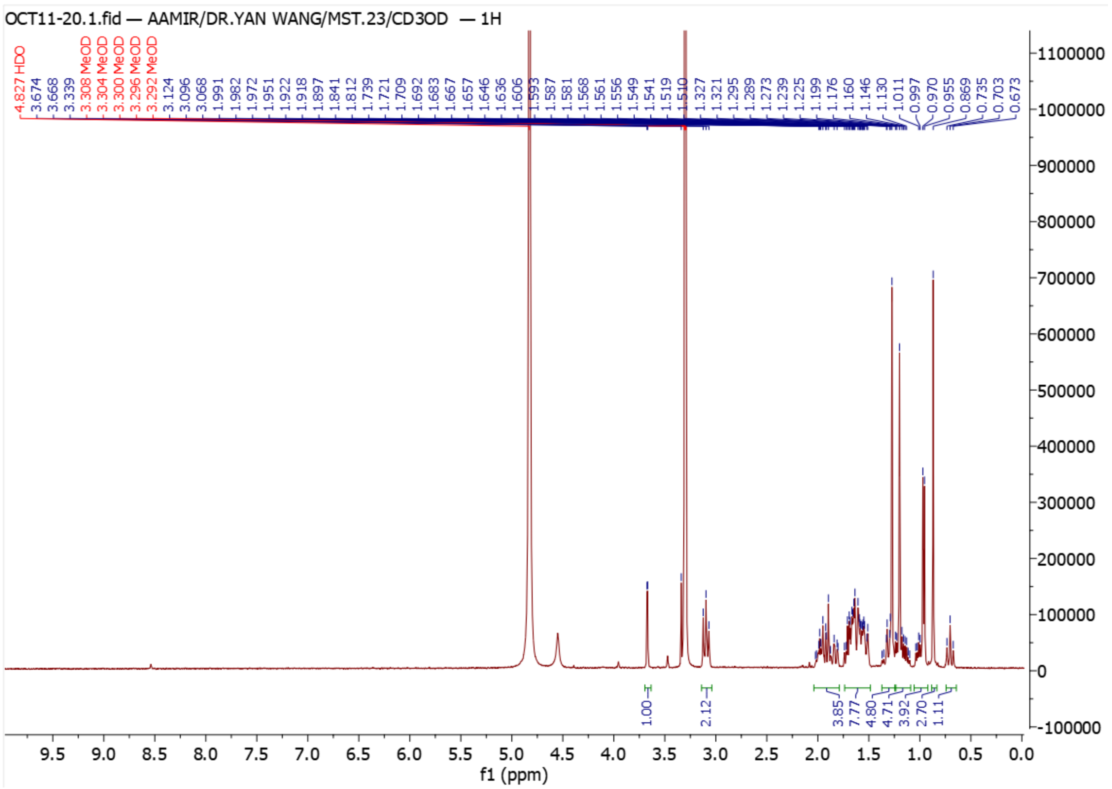

Figure S50. ^{1}H NMR spectrum-1 of compound **5** (400 MHz, CD3OD)

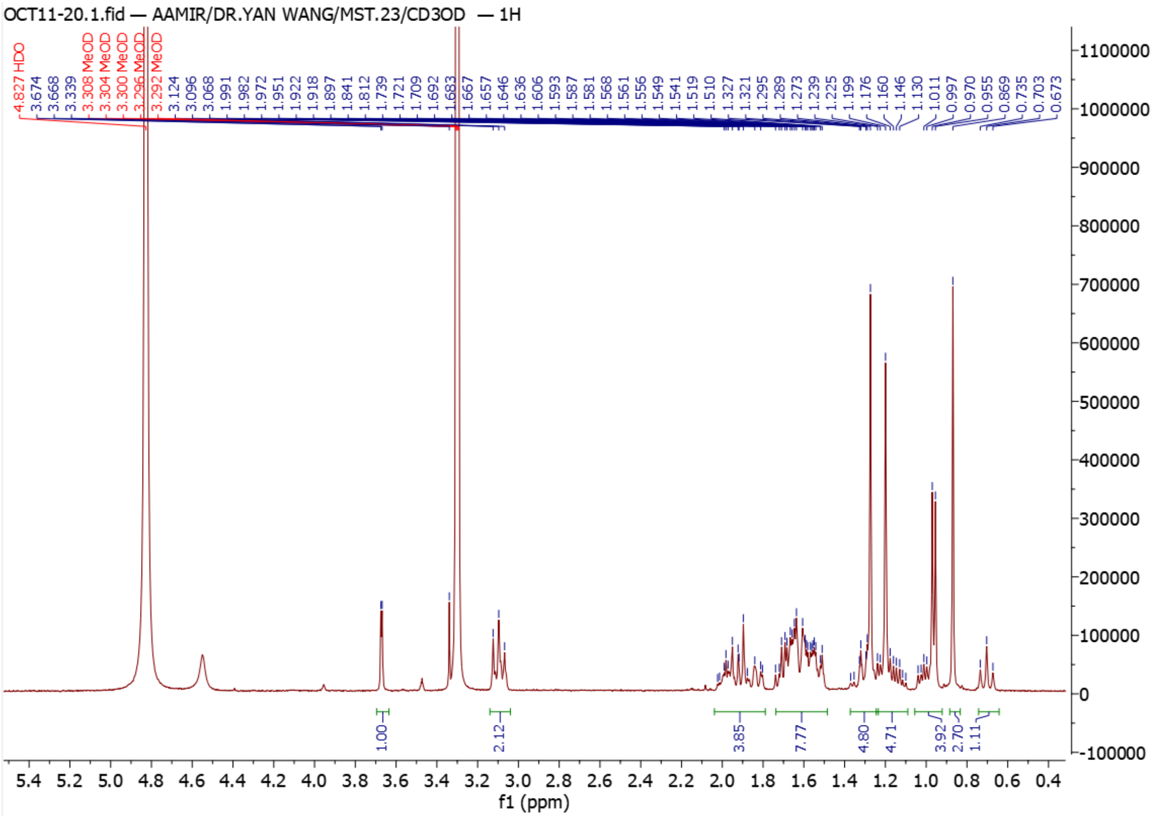

Figure S51. ^{1}H NMR spectrum-2 of compound **5** (400 MHz, CD3OD)

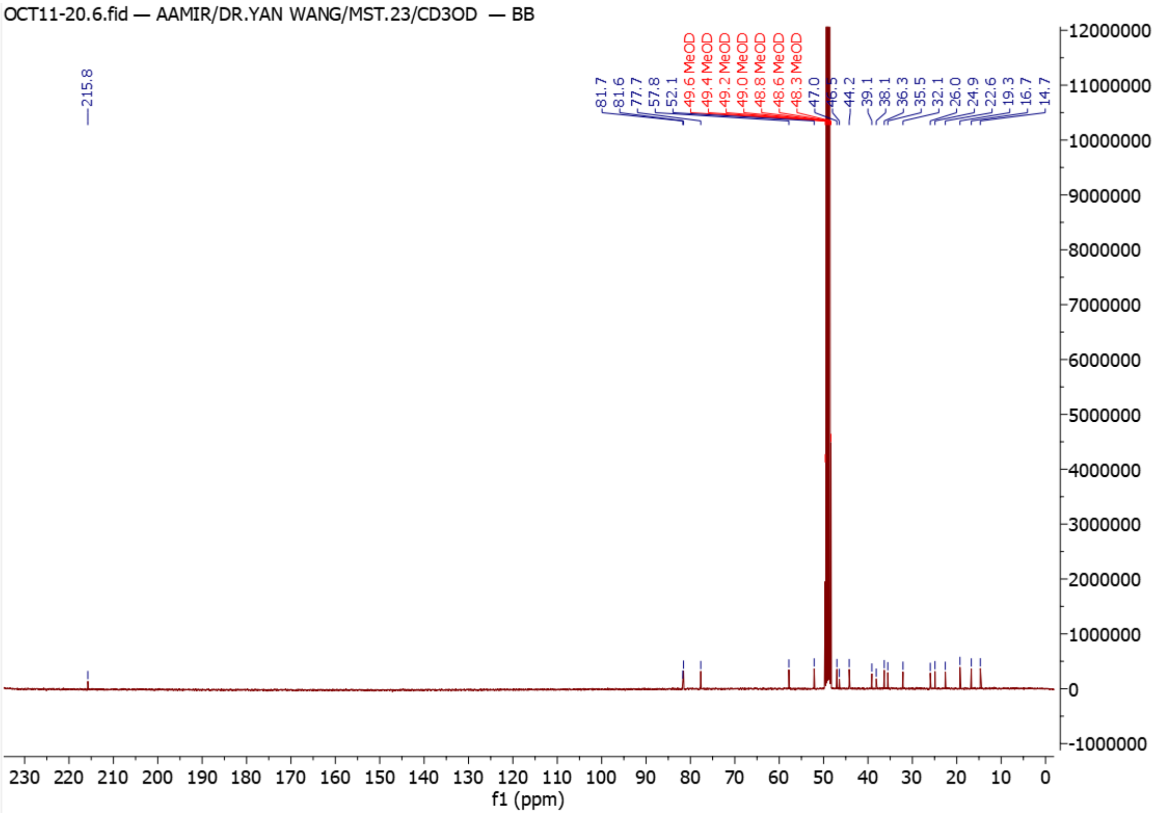

Figure S52. ^{13}C NMR spectrum-1 of compound **5** (100 MHz, CD3OD)

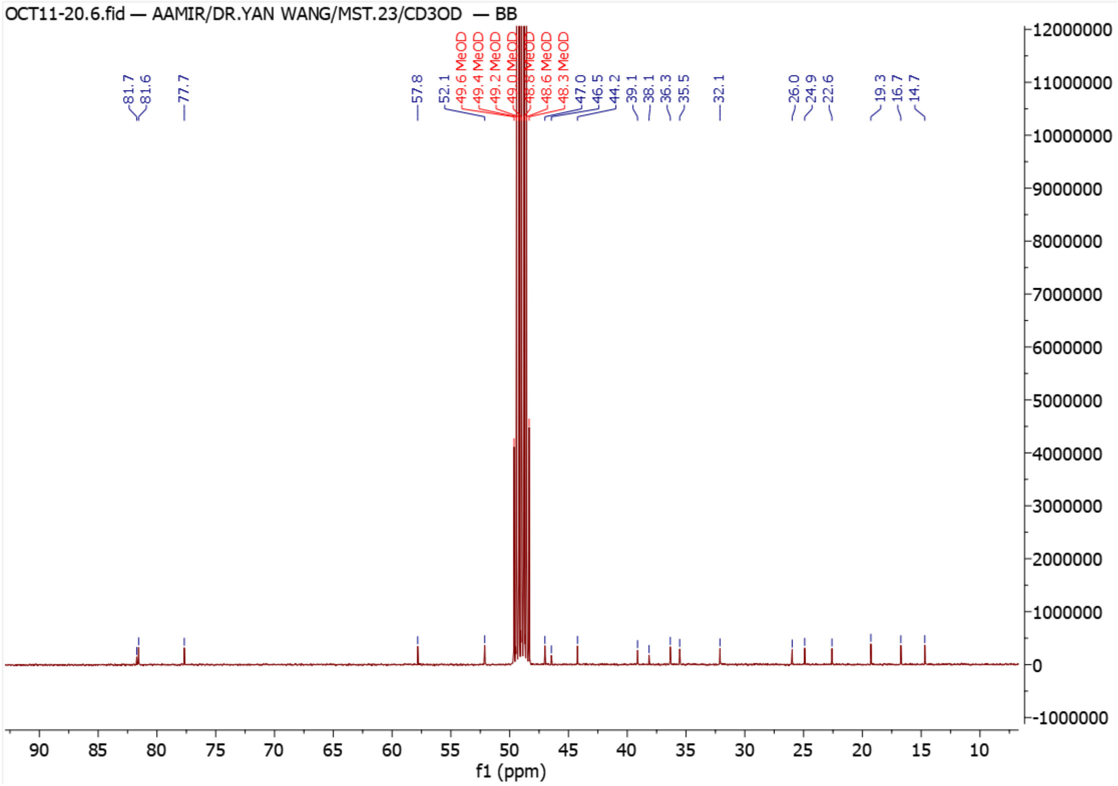

Figure S53. ^{13}C NMR spectrum-2 of compound **5** (100 MHz, CD3OD)

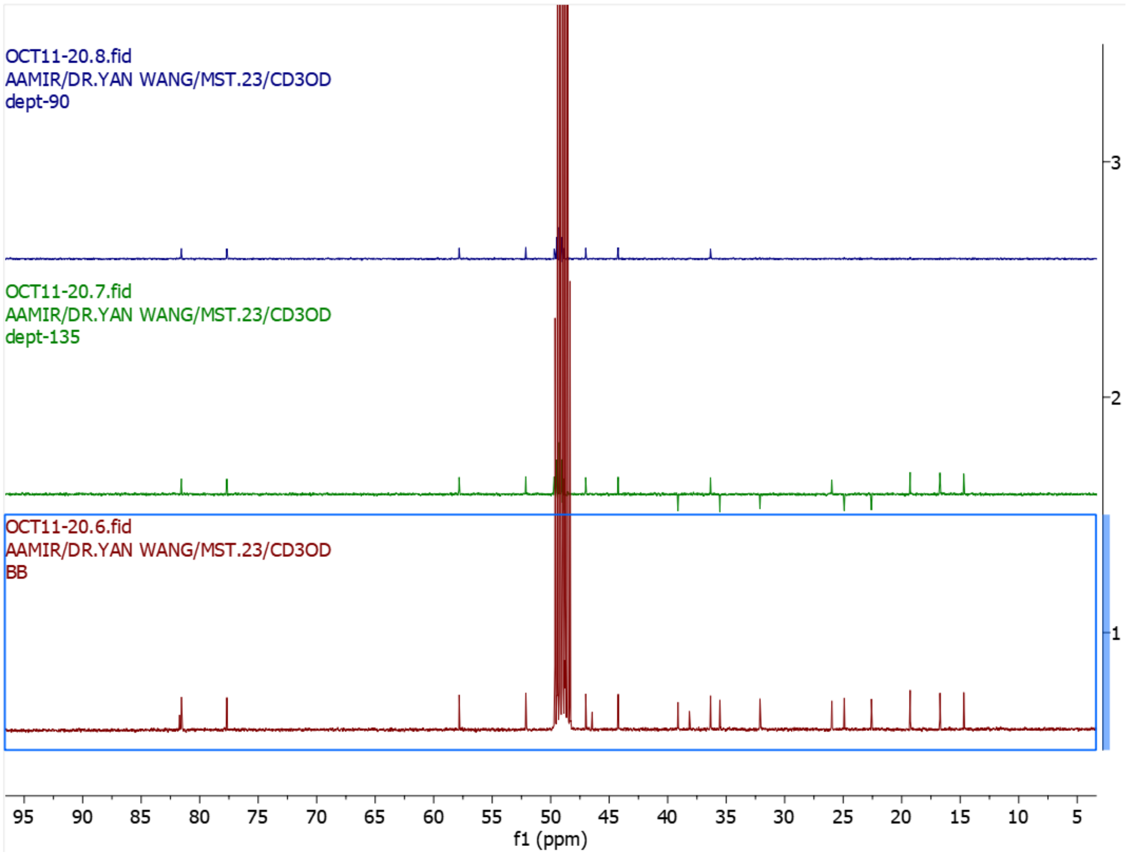

Figure S54. DEPT spectrum of compound **5**

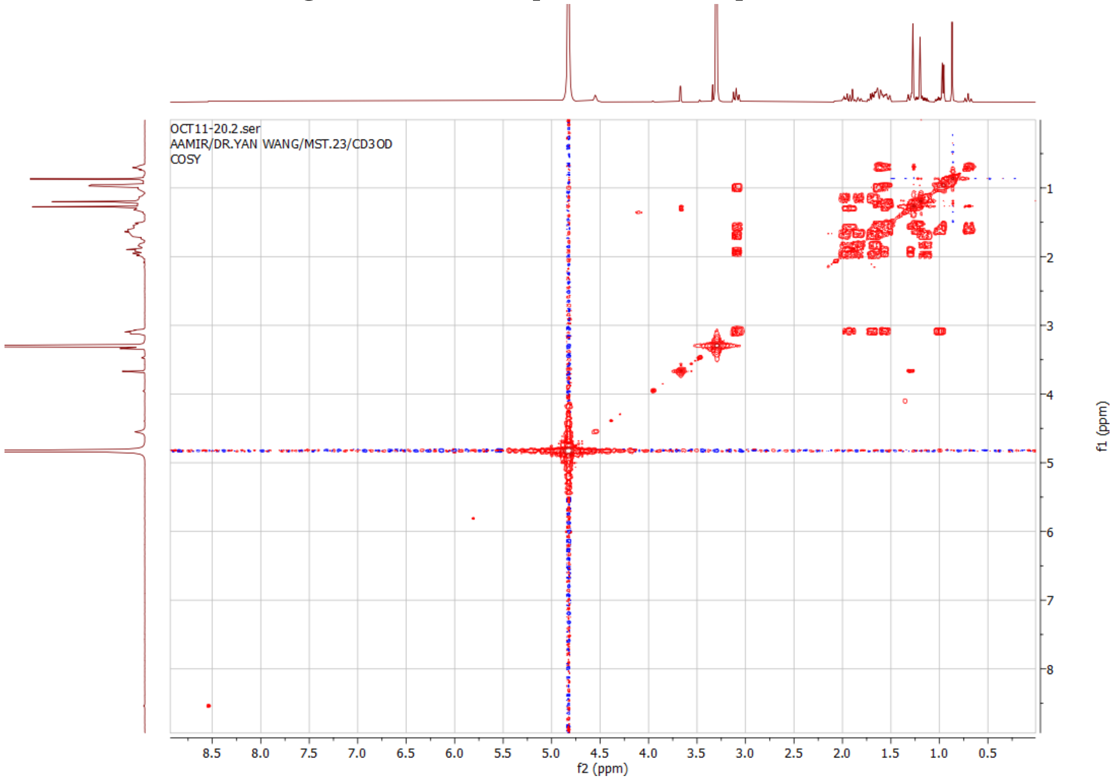

Figure S55. ^{1}H-^{1}H COSY spectrum-1 of compound **5**

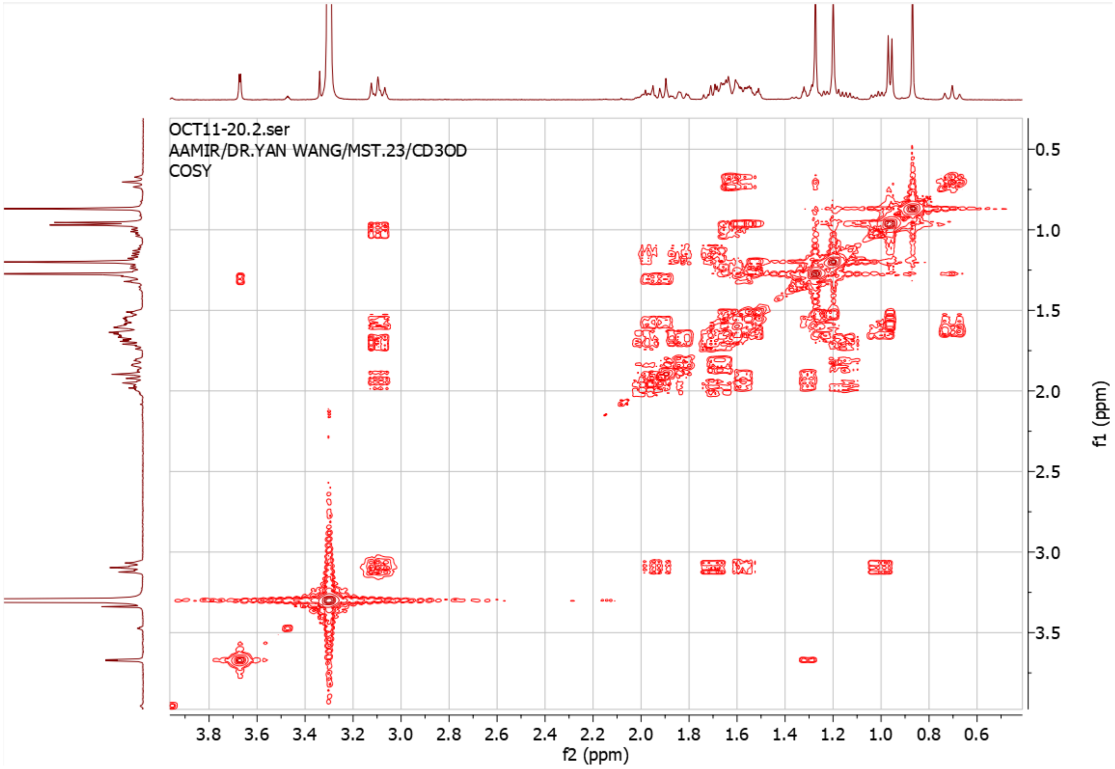

Figure S56. ^{1}H-^{1}H COSY spectrum-2 of compound **5**

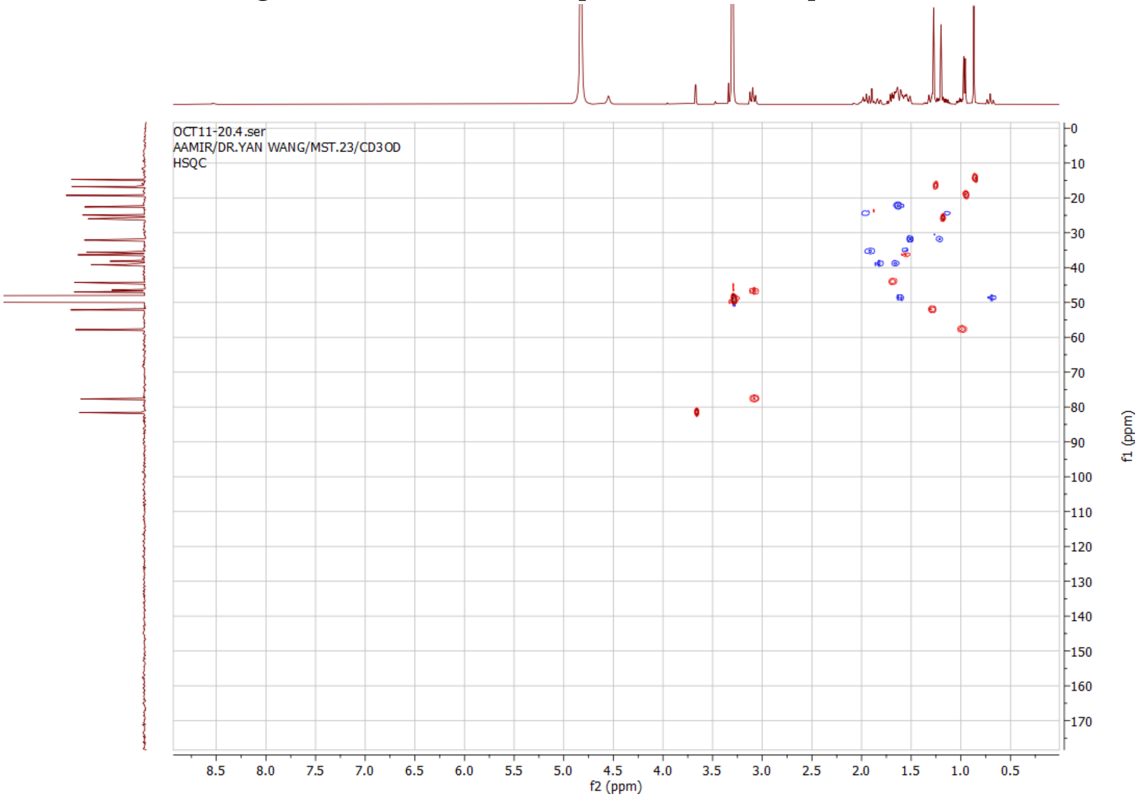

Figure S57. HSQC spectrum-1 of compound **5**

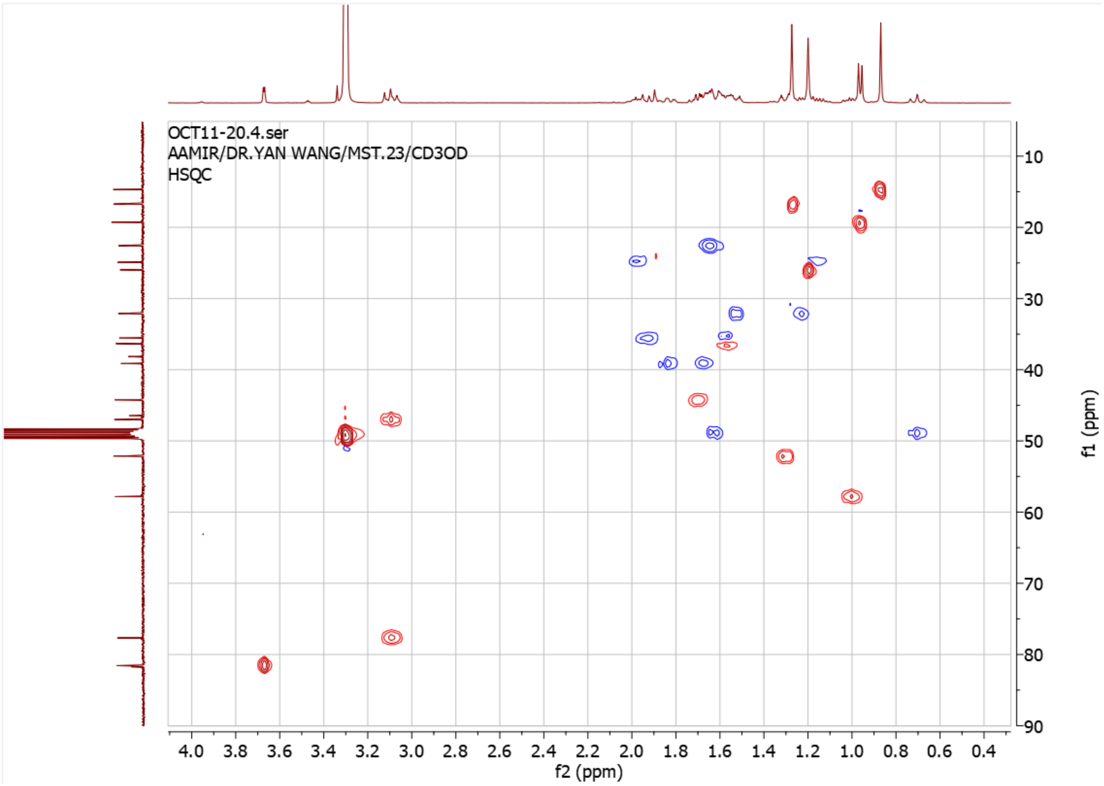

Figure S58. HSQC spectrum-2 of compound **5**

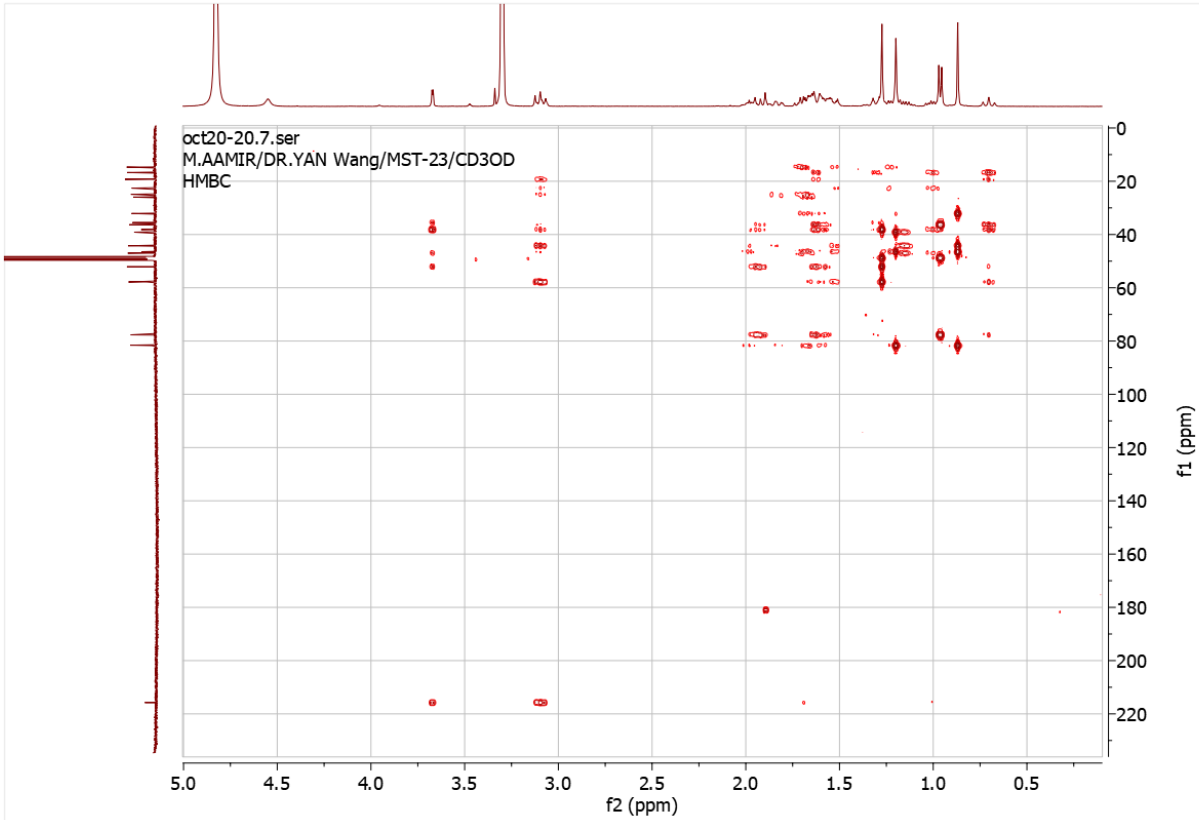

Figure S59. HMBC spectrum-1 of compound **5**

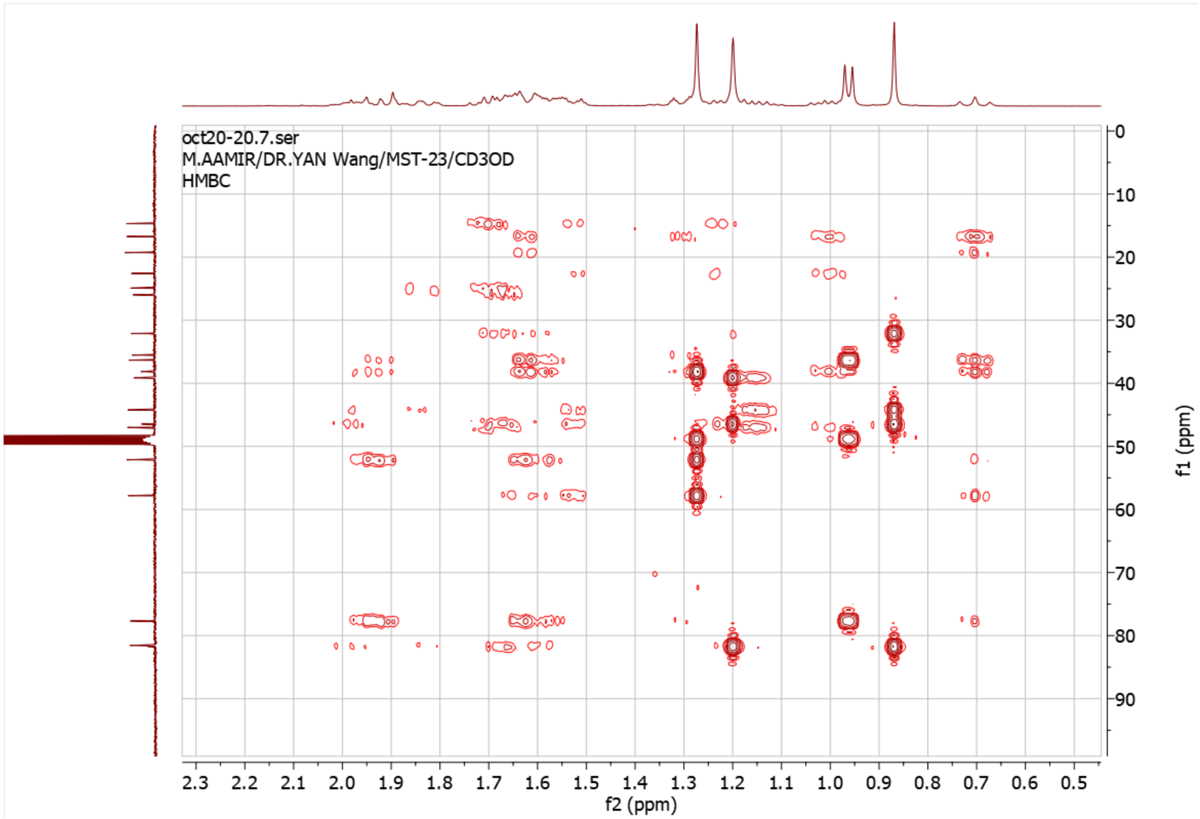

Figure S60. HMBC spectrum-2 of compound **5**

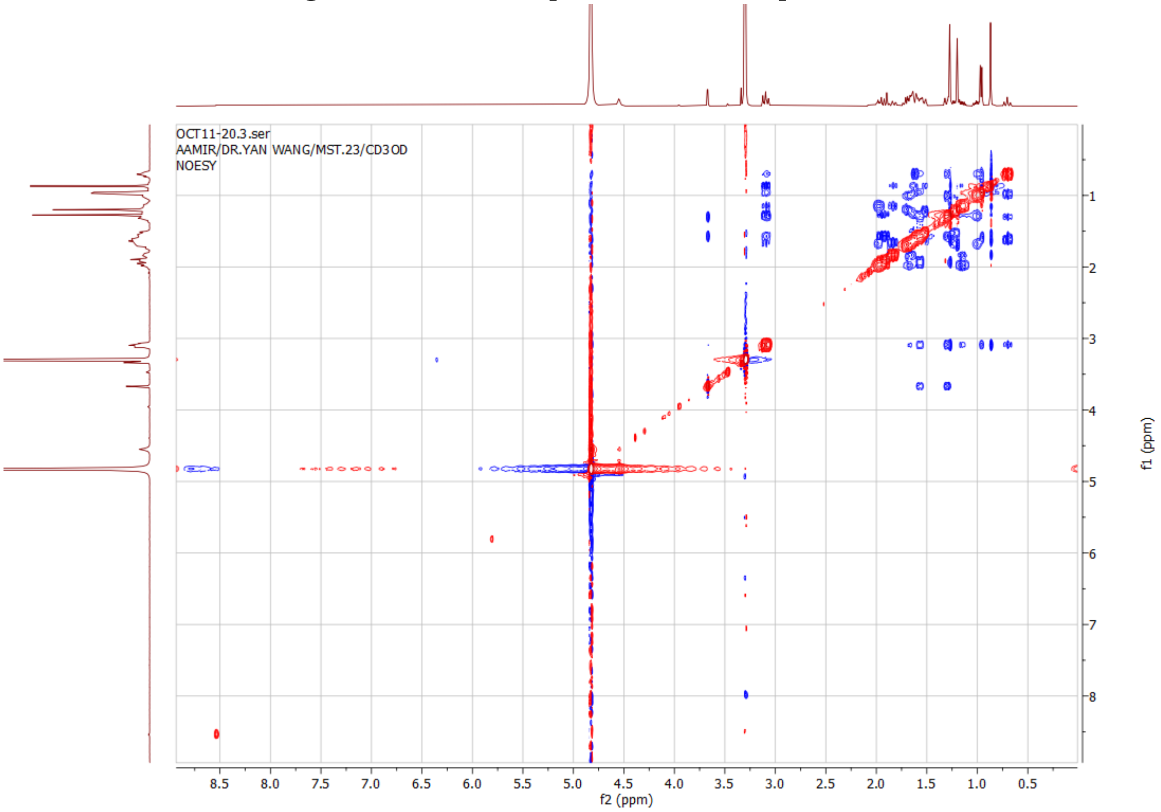

Figure S61. NOESY spectrum-1 of compound **5**

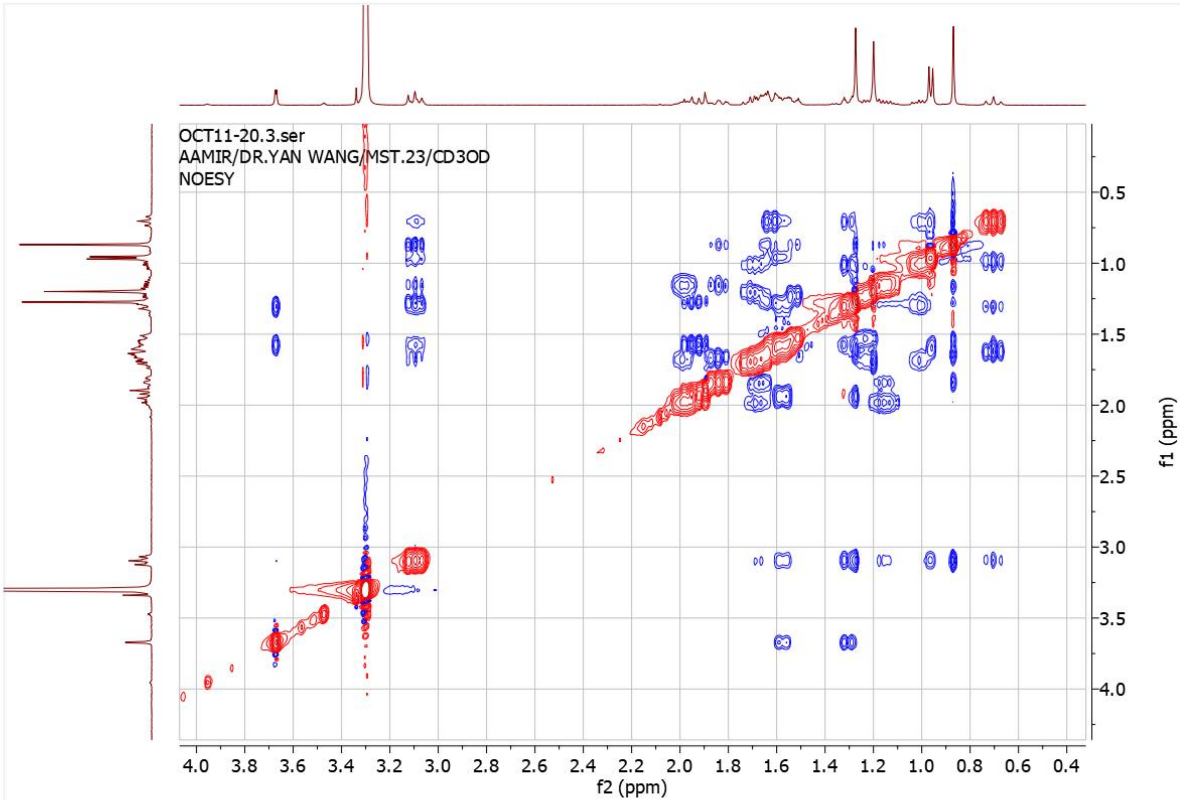

Figure S62. NOESY spectrum-2 of compound **5**

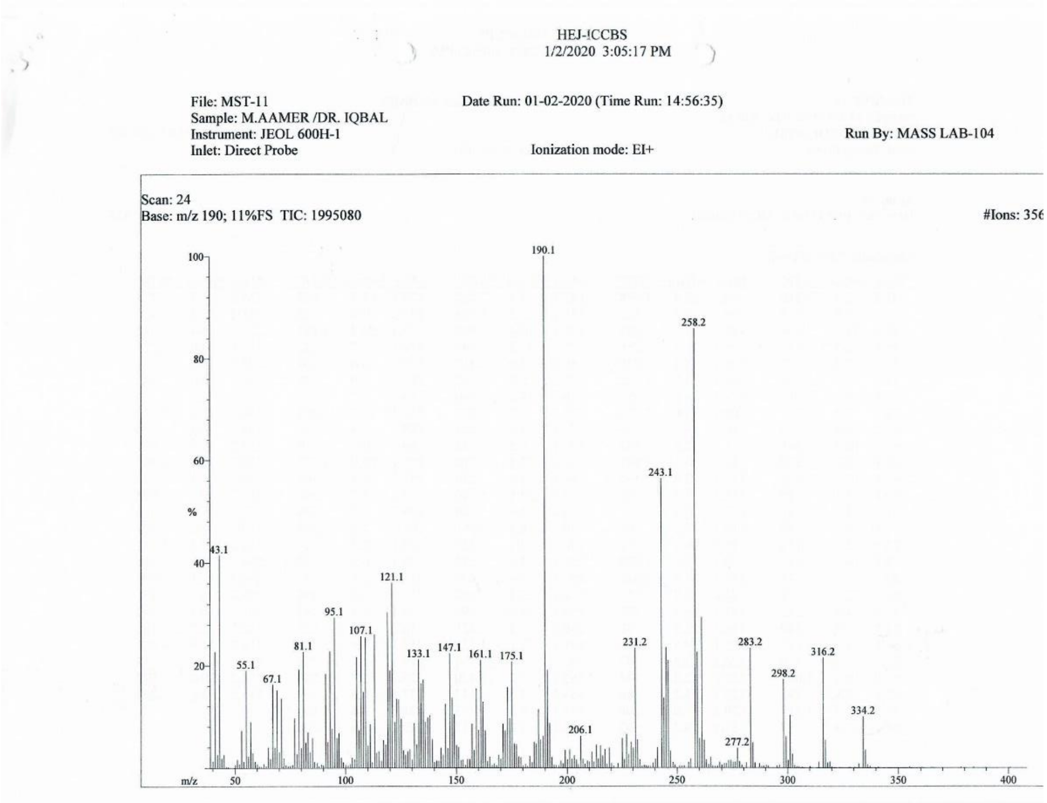

Figure S63. EI-MS spectrum of compound **6**

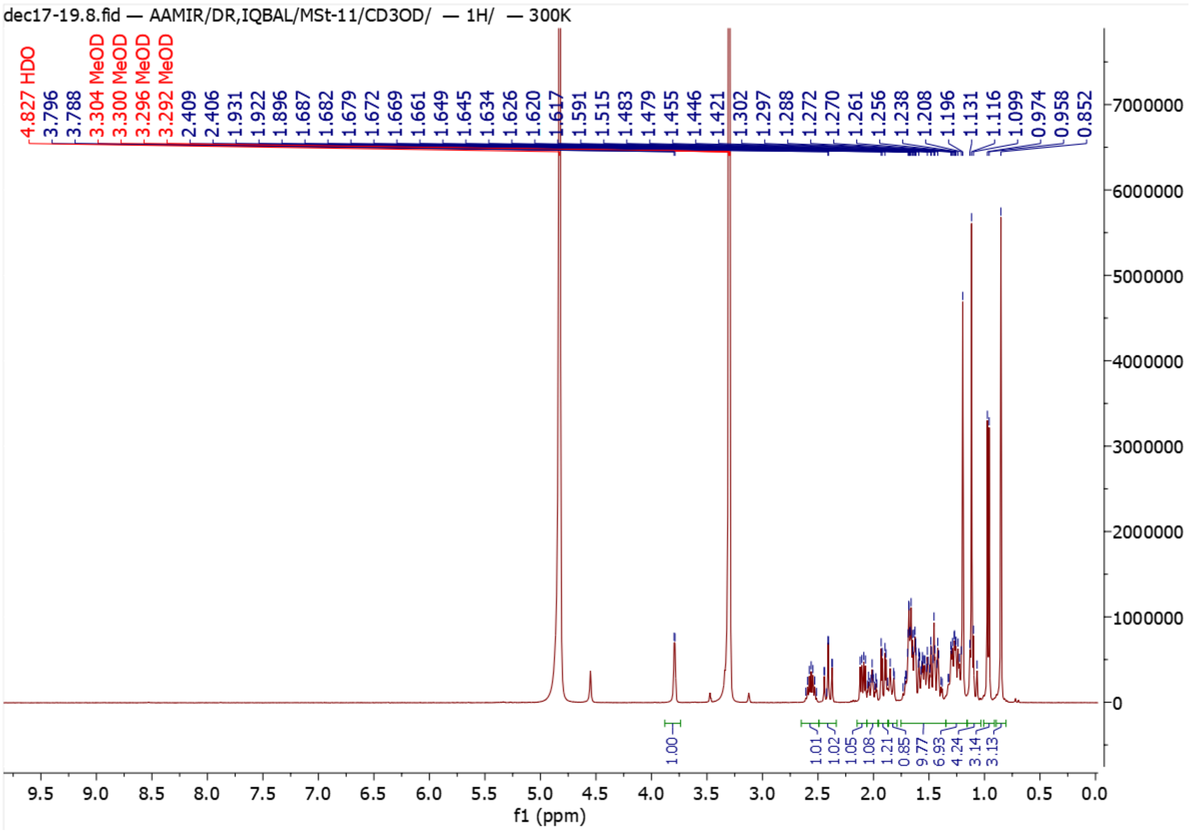

Figure S64. ^{1}H NMR spectrum-1 of compound **6** (400 MHz, CD_3OD)

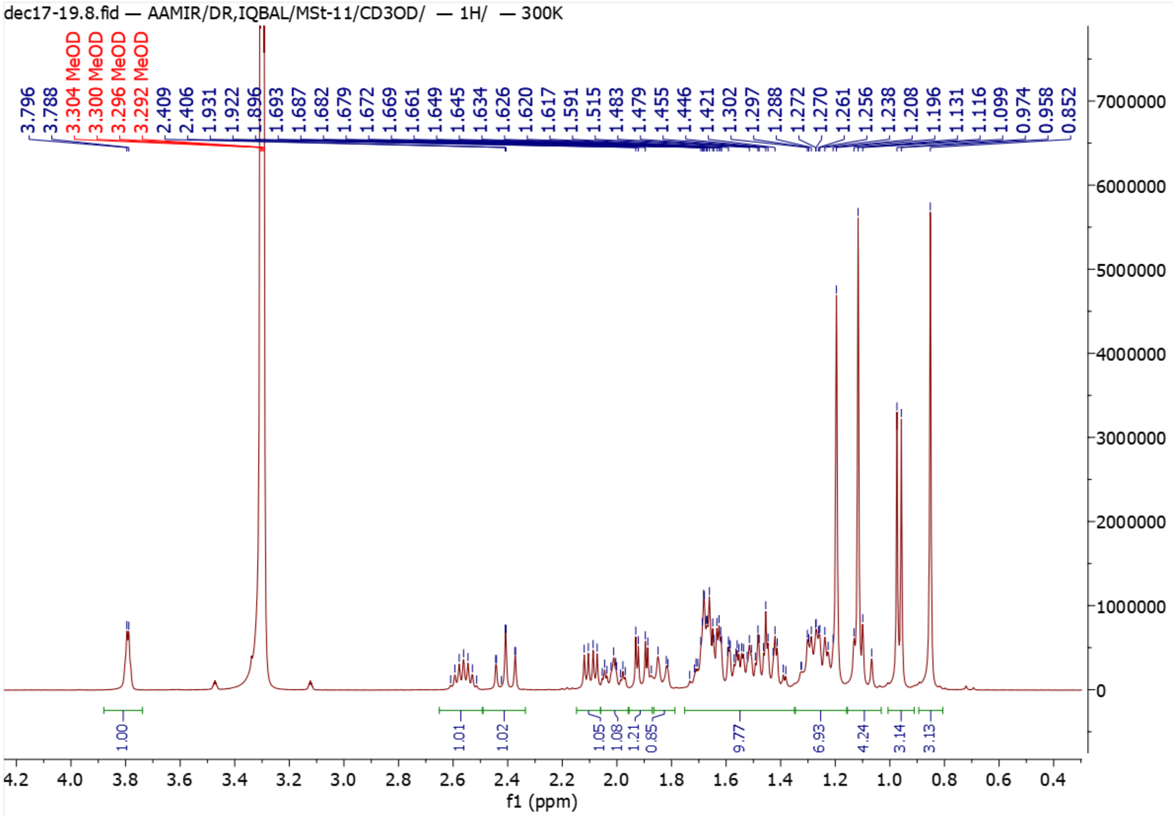

Figure S65. ^{1}H NMR spectrum-2 of compound **6** (400 MHz, CD_3OD)

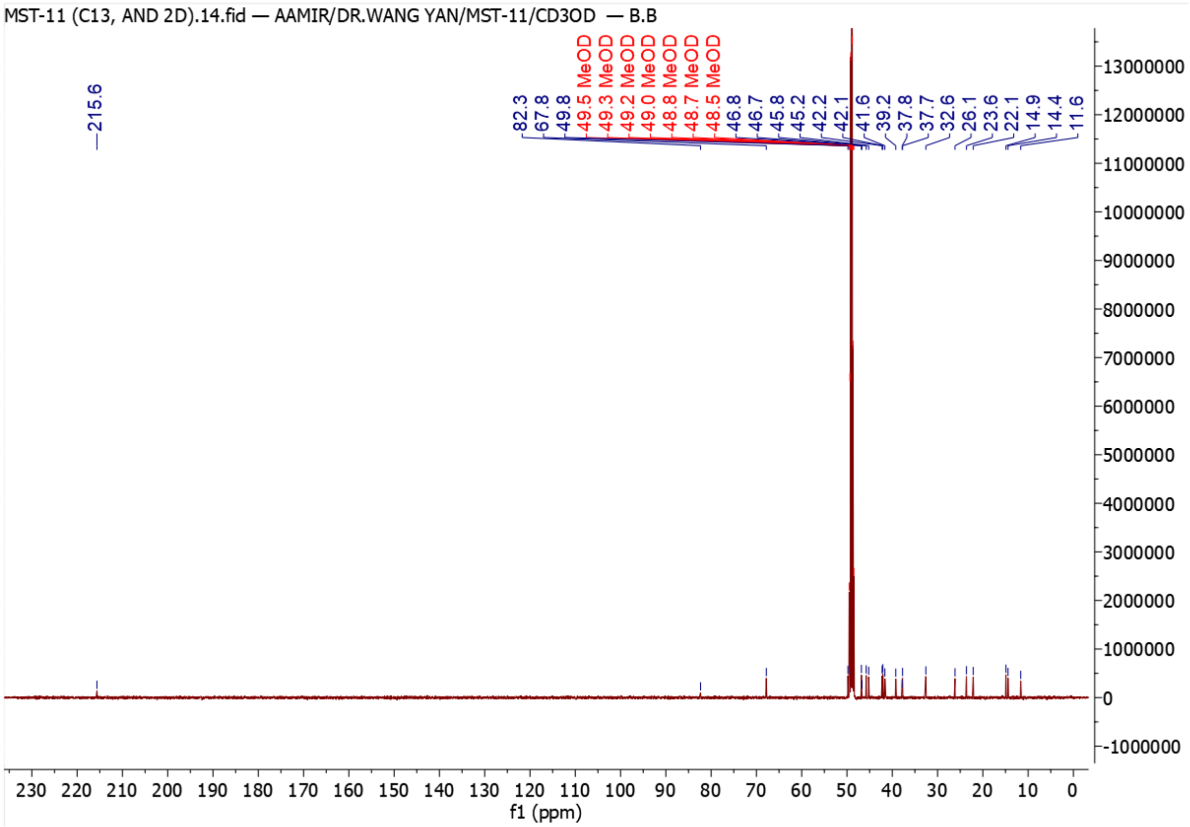

Figure S66. ^{13}C NMR spectrum-1 of compound **6** (125 MHz, CD_3OD)

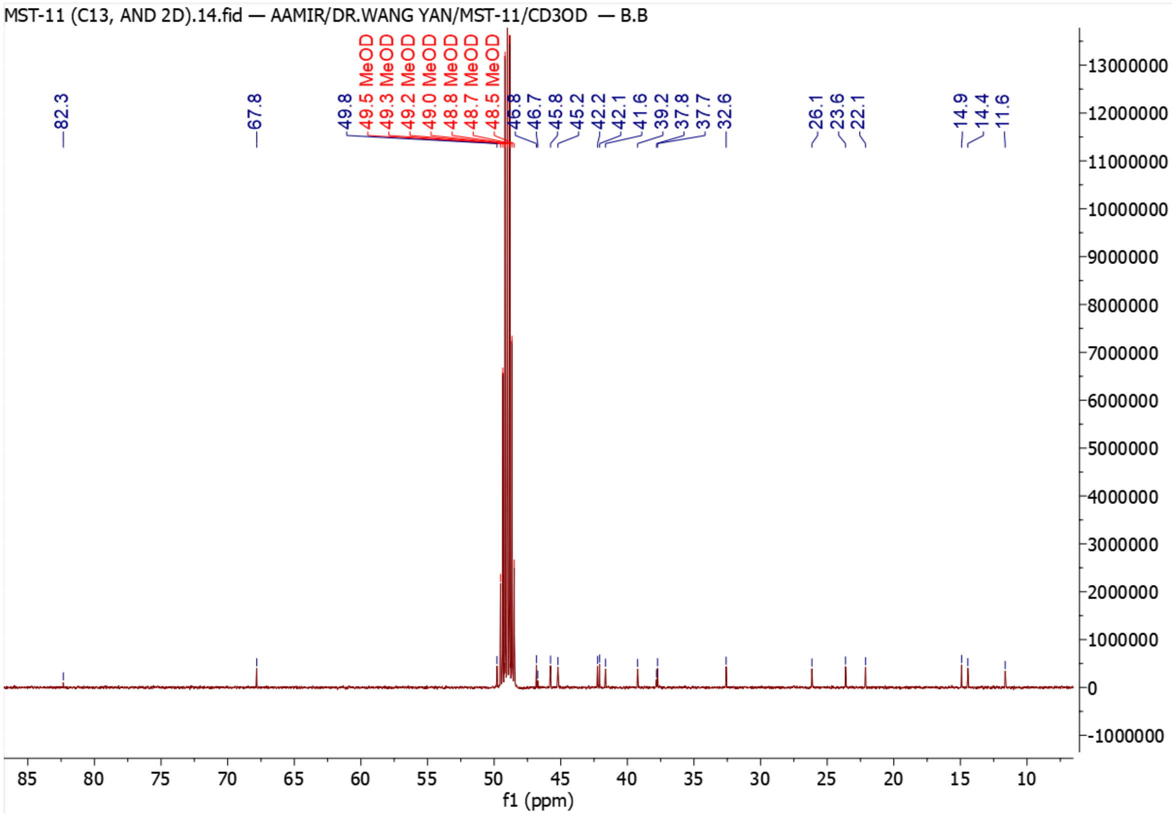

Figure S67. ^{13}C NMR spectrum-2 of compound **6** (125 MHz, CD_3OD)

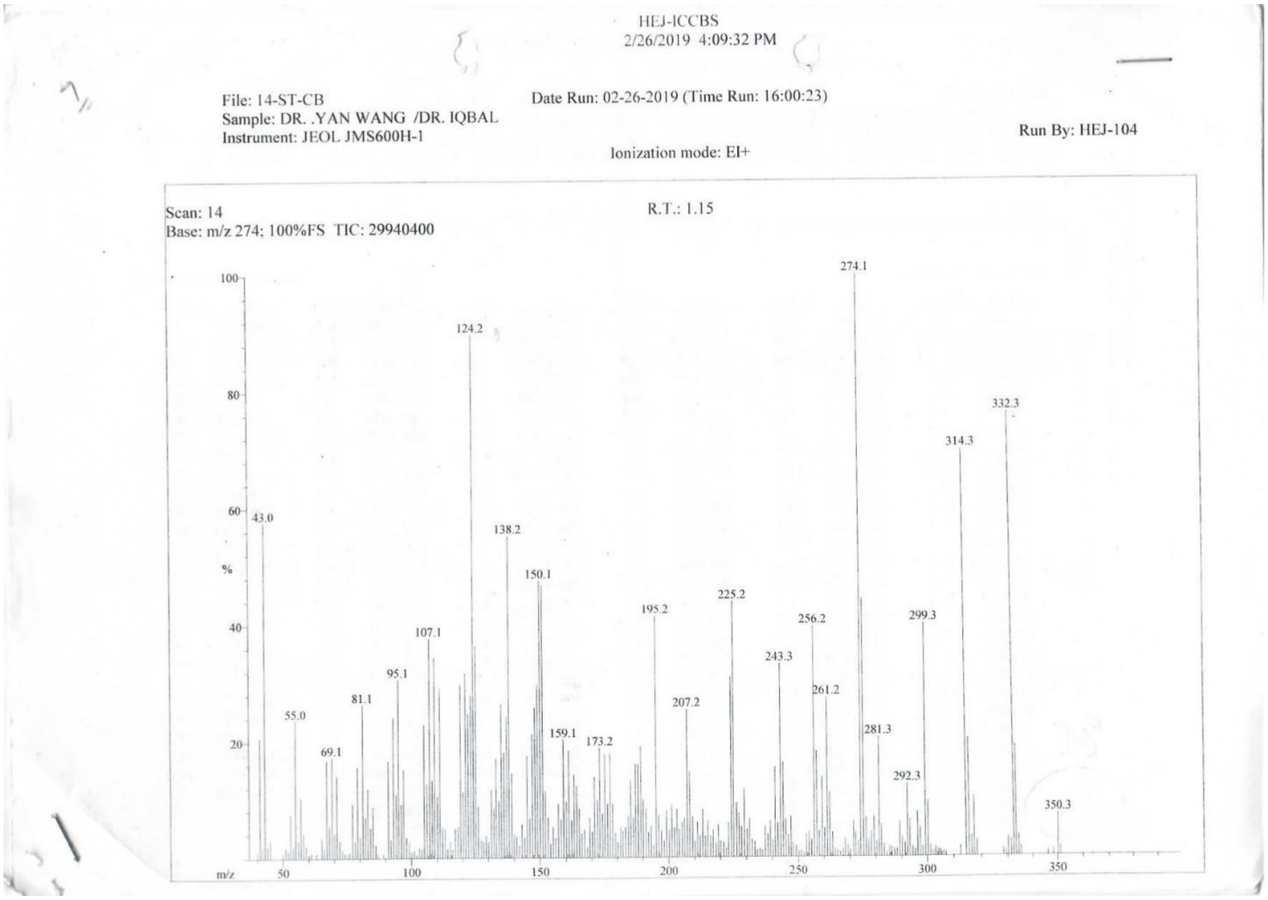

Figure S68. EI-MS spectrum of compound **7**

Mass	Relative Intensity	Theoretical Mass	Delta [ppm]	Delta [mmu]	RDB	Composition
		288.2089	-35.8	-10.3	6.0	$C_{19}H_{28}O_2$
		288.1878	37.5	10.8	11.0	$C_{22}H_{24}$
289.2036	2.8	289.2015	7.4	2.1	1.5	$C_{15}H_{29}O_5$
		289.1956	27.7	8.0	10.5	$C_{22}H_{25}$
290.1837	2.5	290.1882	-15.5	-4.5	6.0	$C_{18}H_{26}O_3$
		290.1729	37.1	10.8	2.0	$C_{14}H_{26}O_6$
291.1950	1.0	291.1960	-3.4	-1.0	5.5	$C_{18}H_{27}O_3$
292.1998	5.5	292.2038	-13.8	-4.0	5.0	$C_{18}H_{28}O_3$
		292.1886	38.5	11.2	1.0	$C_{14}H_{28}O_6$
293.2016	2.5	293.1964	17.7	5.2	0.5	$C_{14}H_{29}O_6$
		293.2117	-34.4	-10.1	4.5	$C_{18}H_{29}O_3$
		293.1905	37.7	11.1	9.5	$C_{21}H_{25}O_1$
294.1993	3.6	294.1984	3.3	1.0	9.0	$C_{21}H_{26}O_1$
		294.2042	-16.6	-4.9	0.0	$C_{14}H_{30}O_6$
295.1953	2.4	295.1909	14.9	4.4	4.5	$C_{17}H_{27}O_4$
		295.2062	-36.8	-10.9	8.5	$C_{21}H_{27}O_1$
296.2178	44.8	296.2140	12.9	3.8	8.0	$C_{21}H_{28}O_1$
297.2143	15.1	297.2218	-25.3	-7.5	7.5	$C_{21}H_{29}O_1$
		297.2066	26.0	7.7	3.5	$C_{17}H_{29}O_4$
298.2207	5.9	298.2144	21.2	6.3	3.0	$C_{17}H_{30}O_4$
		298.2297	-30.0	-8.9	7.0	$C_{21}H_{30}O_1$
299.2063	59.2	299.2011	17.4	5.2	7.5	$C_{20}H_{27}O_2$
300.2086	13.1	300.2089	-1.2	-0.4	7.0	$C_{20}H_{28}O_2$
301.2115	2.9	301.2168	-17.3	-5.2	6.5	$C_{20}H_{29}O_2$
		301.2015	33.3	10.0	2.5	$C_{16}H_{29}O_5$
302.2045	0.9	302.2035	3.5	1.1	11.0	$C_{23}H_{26}$
		302.2093	-15.9	-4.8	2.0	$C_{16}H_{30}O_5$
303.2086	1.1	303.2113	-8.9	-2.7	10.5	$C_{23}H_{27}$
		303.2171	-28.3	-8.6	1.5	$C_{16}H_{31}O_5$
310.1874	1.5	310.1933	-18.9	-5.9	9.0	$C_{21}H_{26}O_2$
		310.1780	30.3	9.4	5.0	$C_{17}H_{26}O_5$
		310.1992	-37.8	-11.7	0.0	$C_{14}H_{30}O_7$
311.2007	1.0	311.2011	-1.3	-0.4	8.5	$C_{21}H_{27}O_2$
312.2026	5.9	312.2089	-20.4	-6.4	8.0	$C_{21}H_{28}O_2$
		312.1937	28.5	8.9	4.0	$C_{17}H_{28}O_5$
313.2052	2.5	313.2015	11.7	3.7	3.5	$C_{17}H_{29}O_5$
		313.1956	30.5	9.5	12.5	$C_{24}H_{25}$
		313.2168	-37.0	-11.6	7.5	$C_{21}H_{29}O_2$
314.2219	75.0	314.2246	-8.4	-2.6	7.0	$C_{21}H_{30}O_2$
315.2170	19.4	315.2171	-0.6	-0.2	2.5	$C_{17}H_{31}O_5$
		315.2113	18.0	5.7	11.5	$C_{24}H_{27}$
316.2283	4.4	316.2250	10.4	3.3	2.0	$C_{17}H_{32}O_5$
		316.2191	29.0	9.2	11.0	$C_{24}H_{28}$
		316.2402	-37.8	-12.0	6.0	$C_{21}H_{32}O_2$
317.2079	8.1	317.2117	-12.0	-3.8	6.5	$C_{20}H_{29}O_3$
		317.1964	36.1	11.5	2.5	$C_{16}H_{29}O_6$
318.2078	1.6	318.2042	11.3	3.6	2.0	$C_{16}H_{30}O_6$
		318.1984	29.8	9.5	11.0	$C_{23}H_{26}O_1$
		318.2195	-36.6	-11.7	6.0	$C_{20}H_{30}O_3$
328.2041	1.9	328.2038	0.7	0.2	8.0	$C_{21}H_{28}O_3$
328.9966	0.9	328.9934	9.8	3.2	13.5	$C_{15}H_5O_9$
		329.0027	-18.7	-6.1	26.5	$C_{26}H_1O_1$
		328.9875	27.7	9.1	22.5	$C_{22}H_1O_4$
		329.0086	-36.5	-12.0	17.5	$C_{19}H_5O_6$
330.2260	3.4	330.2195	19.6	6.5	7.0	$C_{21}H_{30}O_3$
		330.2348	-26.6	-8.8	11.0	$C_{25}H_{30}$
331.2197	1.1	331.2121	23.0	7.6	2.5	$C_{17}H_{31}O_6$
		331.2273	-23.1	-7.6	6.5	$C_{21}H_{31}O_3$
332.2392	36.0	332.2351	12.2	4.1	6.0	$C_{21}H_{32}O_3$
		332.2504	-33.7	-11.2	10.0	$C_{25}H_{32}$
333.2412	8.6	333.2430	-5.3	-1.8	5.5	$C_{21}H_{33}O_3$
334.2512	1.4	334.2508	1.3	0.4	5.0	$C_{21}H_{34}O_3$
350.2434	2.1	350.2457	-6.7	-2.4	5.0	$C_{21}H_{34}O_4$

Figure S69. HREI-MS spectrum of compound **7**

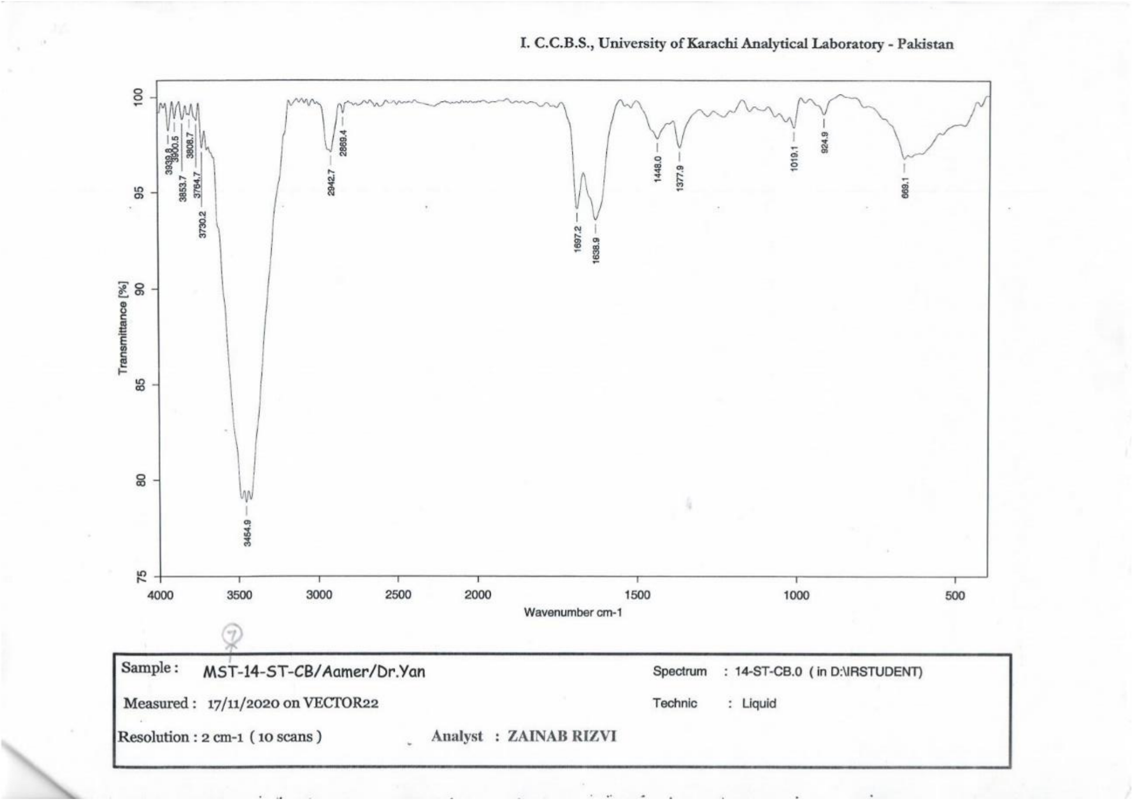

Figure S70. IR spectrum of compound **7**

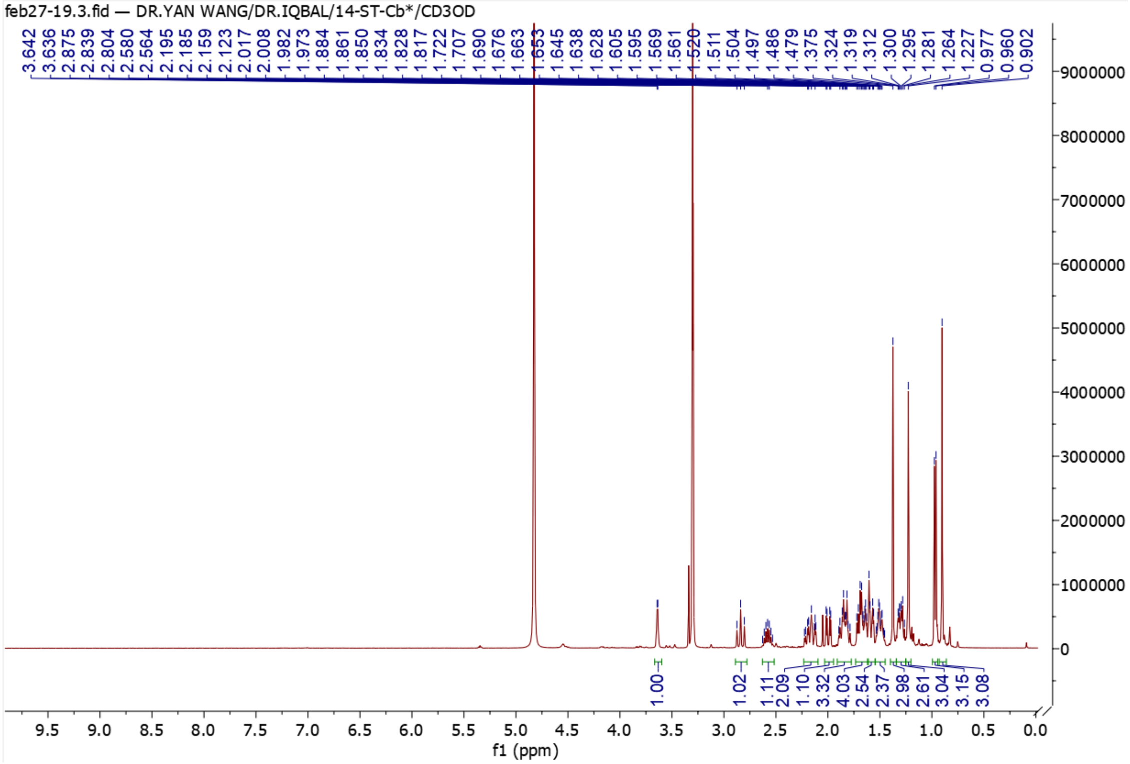

Figure S71. ^{1}H NMR spectrum-1 of compound **7** (400 MHz, CD3OD)

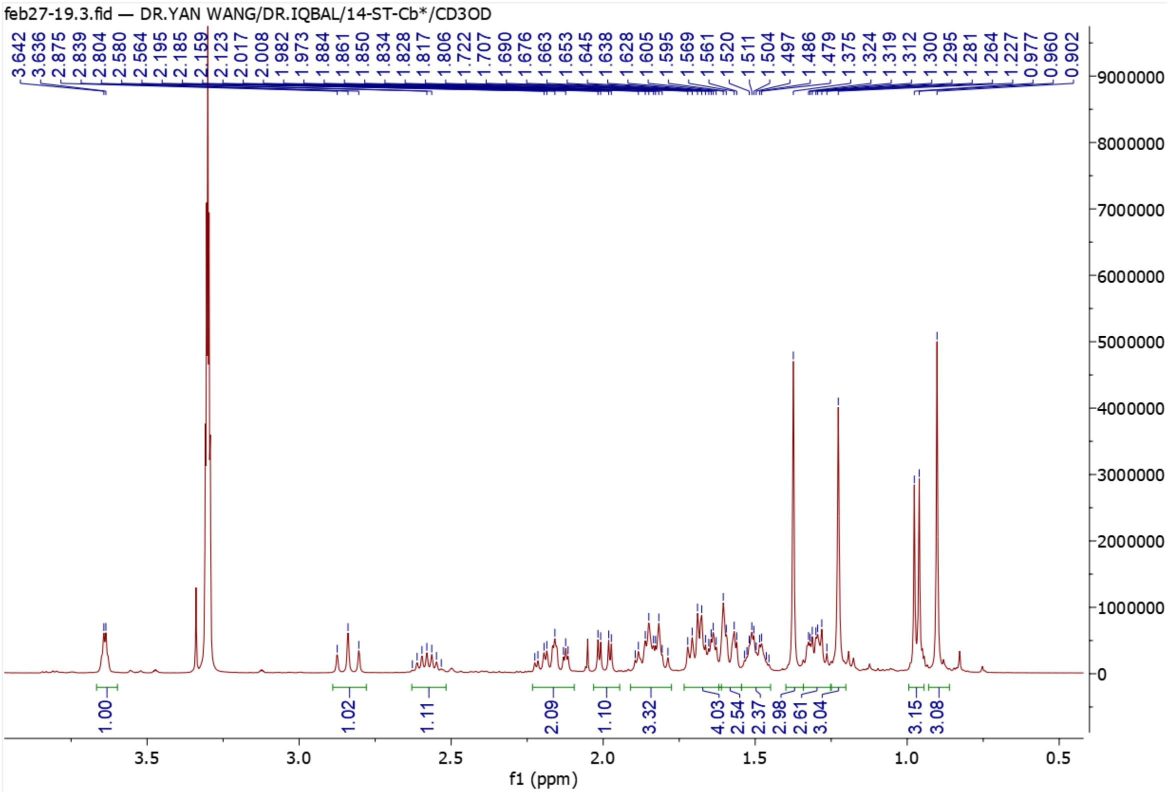

Figure S72. ^{1}H NMR spectrum-2 of compound **7** (400 MHz, CD3OD)

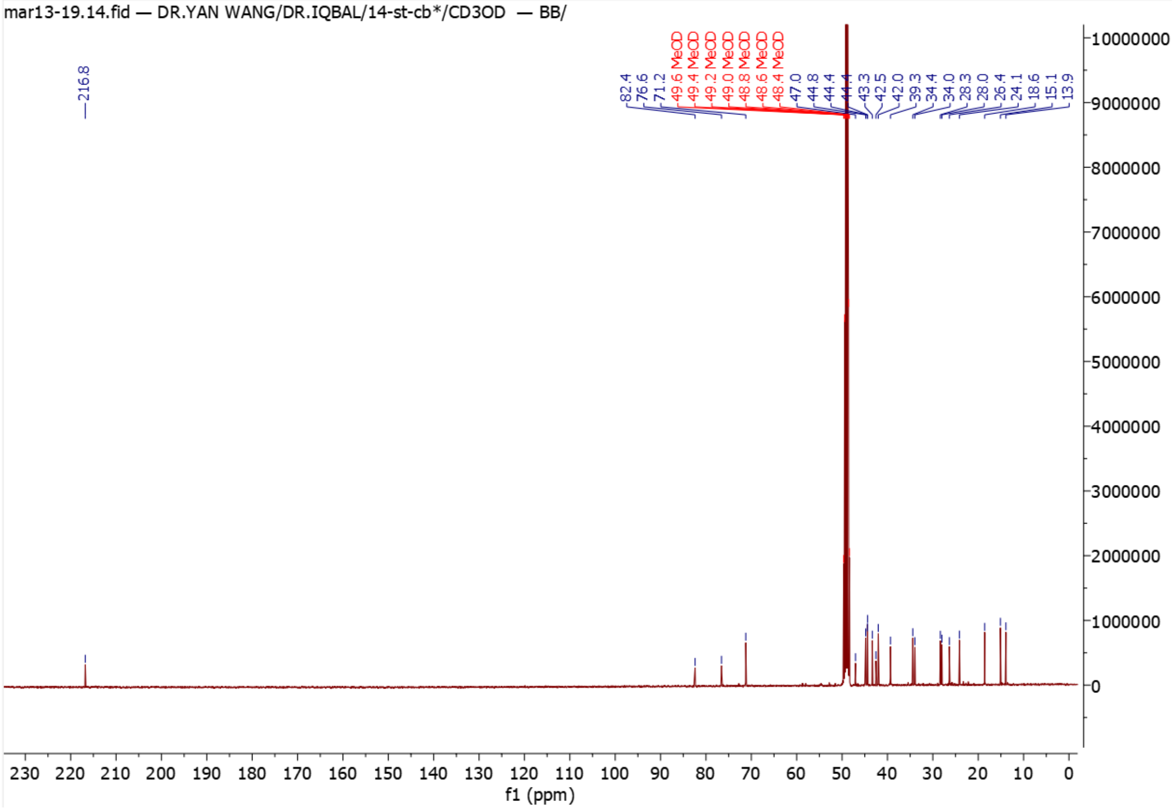

Figure S73. ^{13}C NMR spectrum-1 of compound **7** (100 MHz, CD3OD)

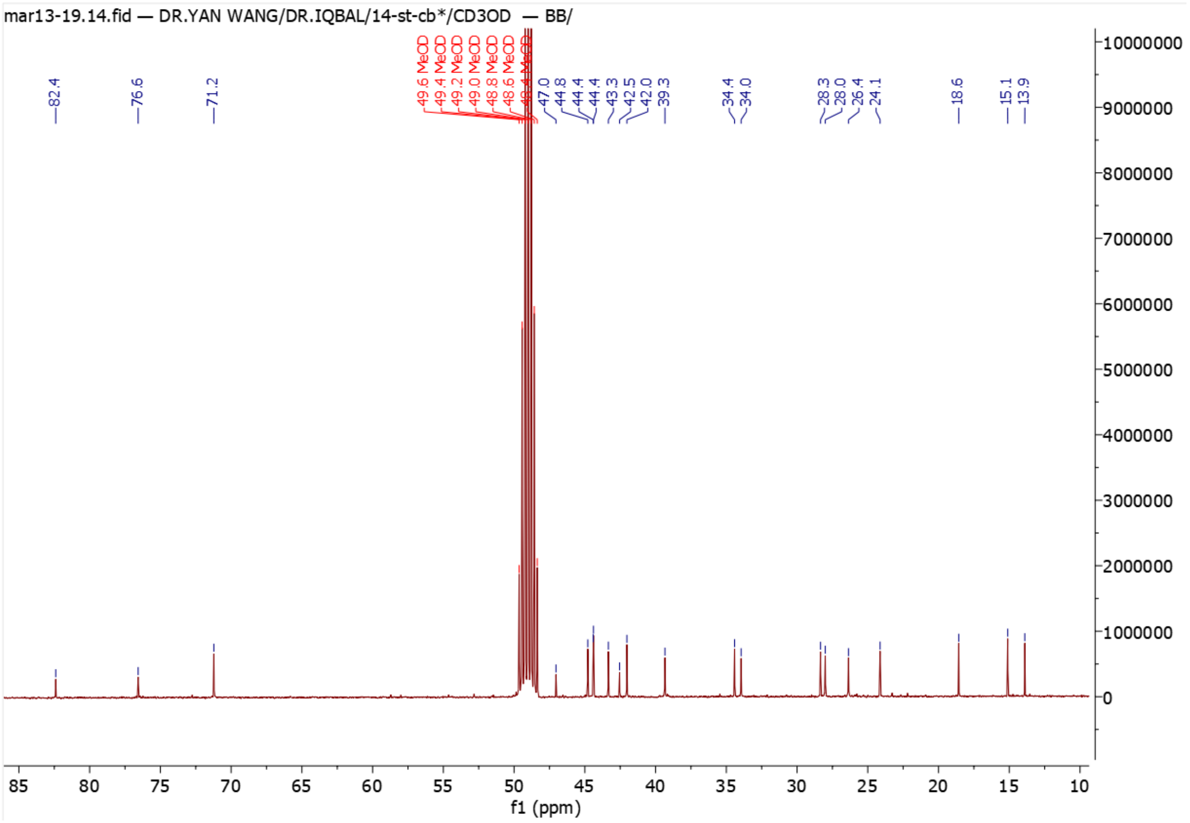

Figure S74. ^{13}C NMR spectrum-2 of compound **7** (100 MHz, CD3OD)

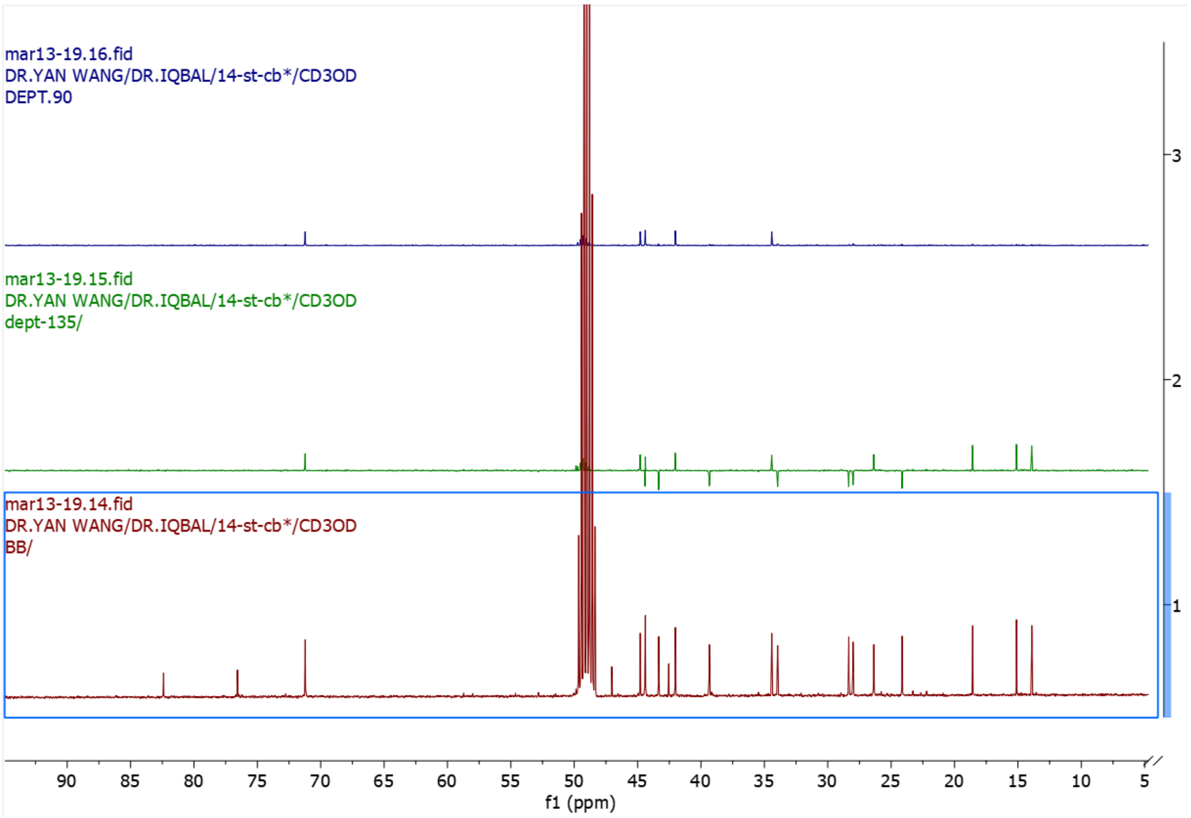

Figure S75. DEPT spectrum of compound **7**

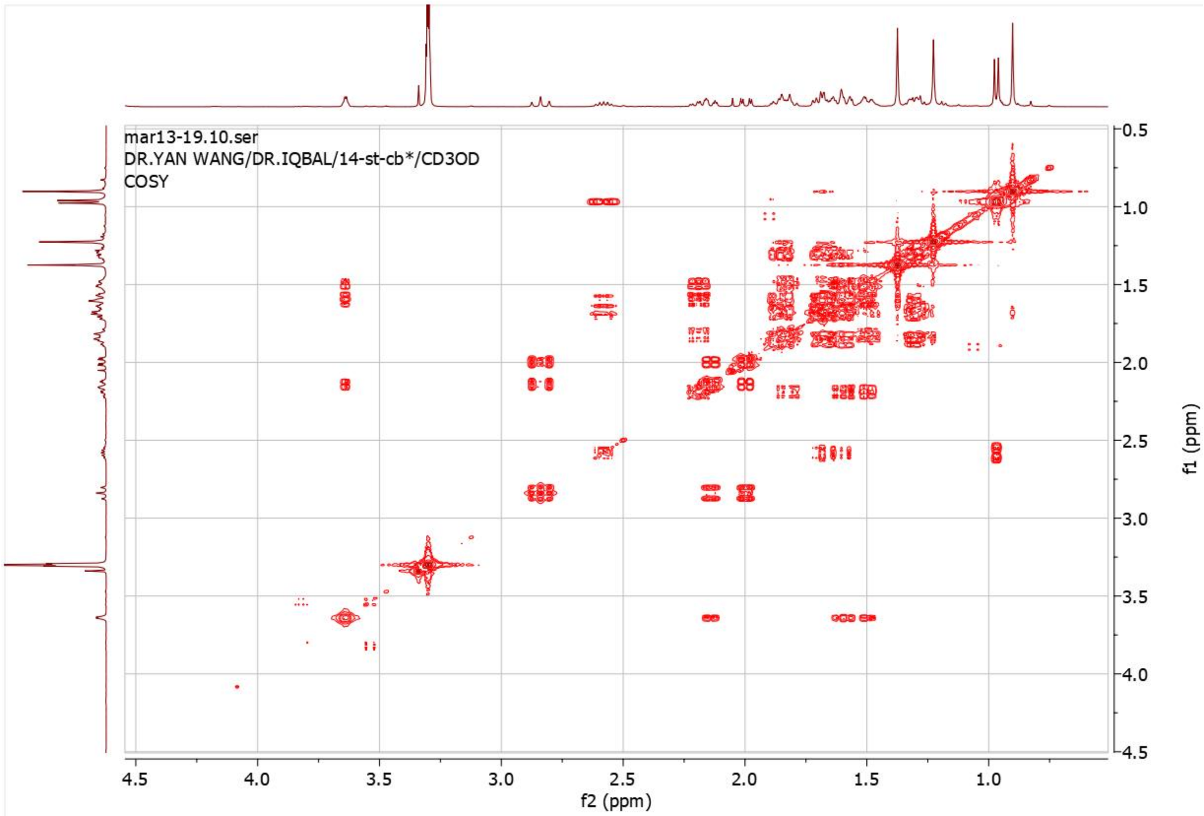

Figure S76. ^{1}H-^{1}H COSY spectrum of compound **7**

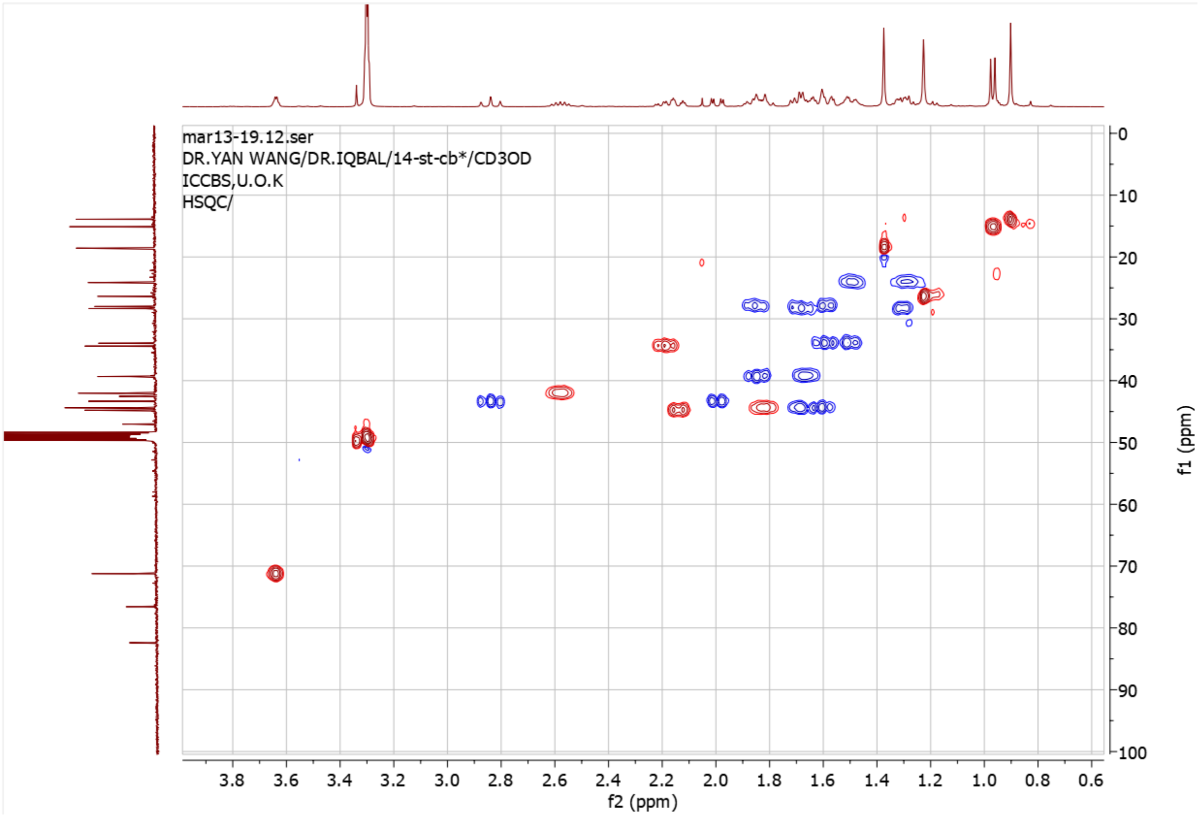

Figure S77. HSQC spectrum of compound **7**

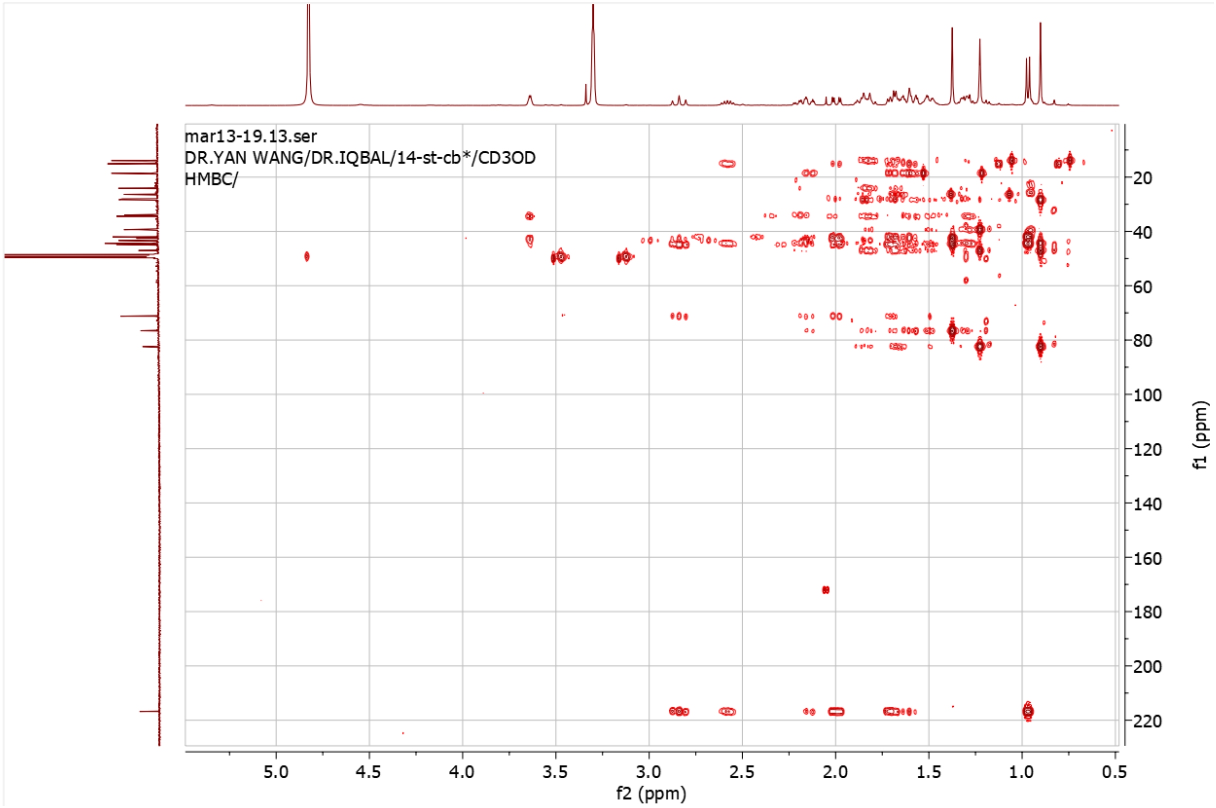

Figure S78. HMBC spectrum-1 of compound **7**

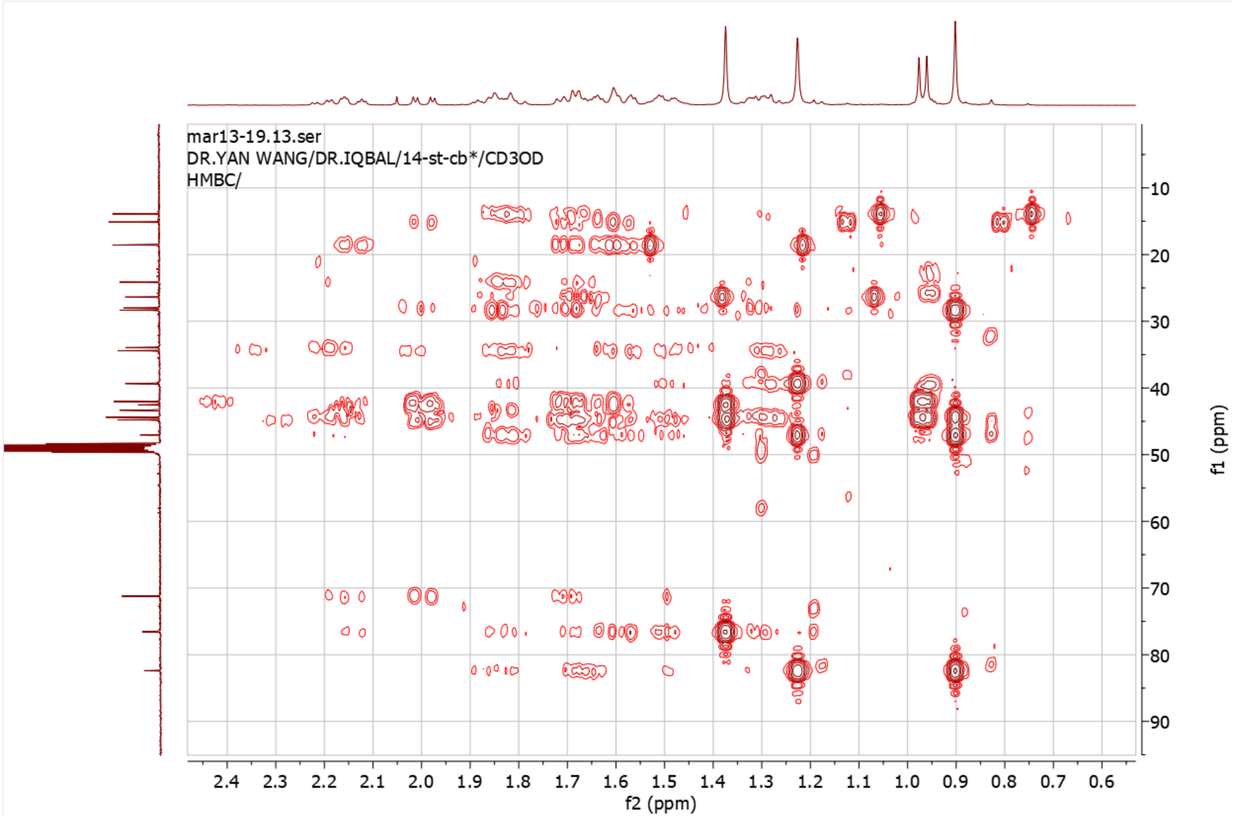

Figure S79. HMBC spectrum-2 of compound **7**

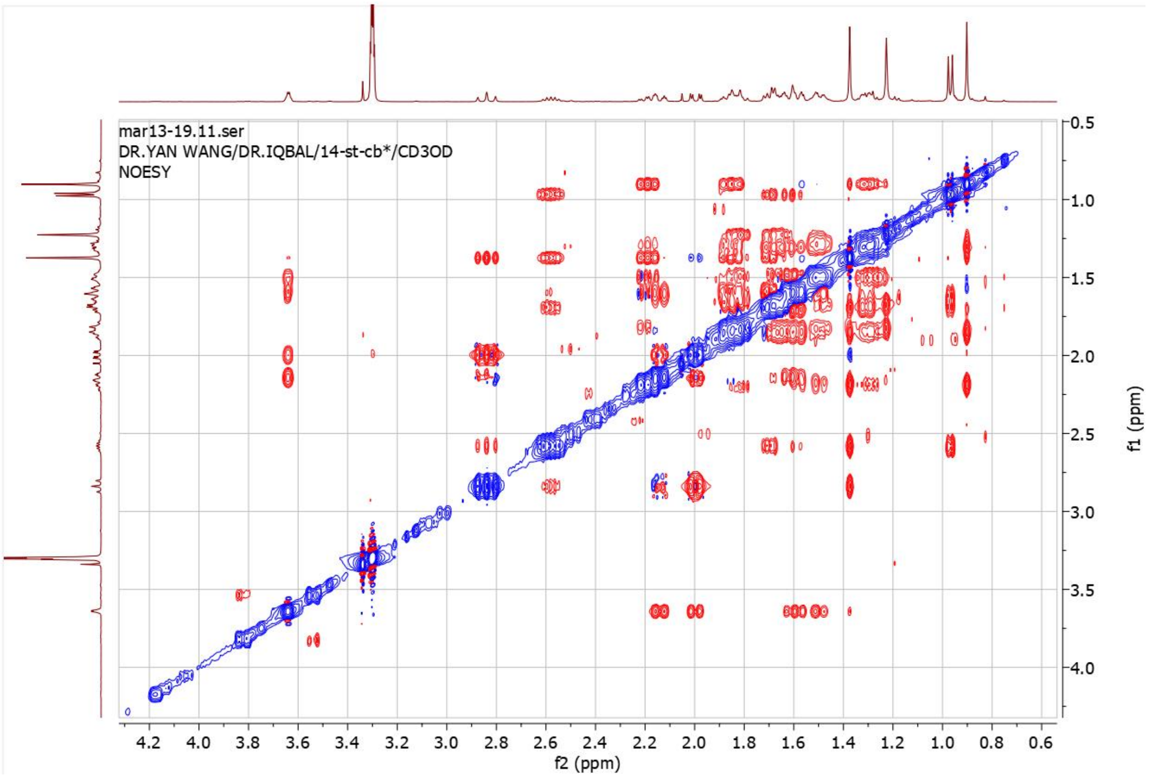

Figure S80. NOESY spectrum of compound **7**
